# Supplementary material for: Preferences for different diagnostic modalities to follow up abnormal colorectal cancer screening results: a hypothetical vignette study
Source: BMJ Open. 2020 Jul 26;10(7):e035264. doi: 10.1136/bmjopen-2019-035264 (PMC7383951; doi:10.1136/bmjopen-2019-035264)
Supplement: Supplementary data [file bmjopen-2019-035264supp001.pdf]

Investigating offers of alternative investigations after a positive bowel screening test

Welcome page

Thank you for agreeing to complete this survey.

It should take you no longer than 10 minutes to complete. You may, however, take as long as you want.

No identifiable information will be collected, meaning that your responses will be anonymous and it will not be possible for you to be identified as an individual. All information will be handled in accordance with the provisions of the Data Protection Act, 1998 and the General Data Protection Regulation (2018).

Please try to respond to **ALL** the questions – an incomplete questionnaire will make your responses less valid.

Information sheet

Title of Project: Investigating offers of alternative investigations after a positive bowel screening test

This study has been approved by the UCL Research Ethics Committee: Project ID number: 14687/001

We would like to invite you to take part in this study. You should only take part if you want to. Before you decide whether to take part, it is important for you to read the following information carefully and discuss it with others if you wish. Please ask us if anything is unclear or if you would like more information.

What is the purpose of the study?

This study is concerned with how individuals make choices related to bowel cancer screening. To explore this further, we have developed a survey to ask people about their preferences.

Why have I been invited?

We are inviting men and women aged between 60 and 74 years or older to take part in this survey.

Do I have to take part?

It is up to you to decide to join the study. If you decide to withdraw you will be asked what you wish to happen to the data you have provided up that point.

What will I have to do if I take part?

If you choose to take part we would ask you to complete an online survey which should take around 10 minutes to complete.

What are the possible disadvantages and risks of taking part?

This study carries very little risk for the participants. There are no right or wrong answers to the questions. As we are asking you about health issues that may be sensitive, there is a slight possibility that you will feel worried.

What are the possible benefits of taking part?

In addition to the remuneration you receive from SSI, this study will help us to better understand people's preferences for different bowel cancer screening tests.

What if there is a problem?

If you have a concern about any aspects of the study you should in the first instance contact Aradhna Kaushal (aradhna.kaushal.14@ucl.ac.uk) who will do her best to answer your questions. If you would like to discuss a problem with someone not directly involved in the project, you can contact the chairs of UCLs research ethics committees (ethics@ucl.ac.uk).

Will my taking part in this project be kept confidential?

Yes. All information which is collected about you during the course of the research is anonymous and not linked to any of your personal details. This means that you cannot be recognised from the information you provide. Our procedures for storage, processing, handling and destroying the information you give us are in line with Data Protection Act (1998) and the General Data Protection Regulation (2016). The survey will be conducted using SurveyMonkey which stores data collected on servers located in the United States.

Kaushal A, *et al. BMJ Open* 2020; 10:e035264. doi: 10.1136/bmjopen-2019-035264

What will happen to the results of the research project?

We hope to report the findings from the study in an academic journal. You will not be identified in any reports or publications from the study.

Who is organising and funding the research?

Dr Aradhna Kaushal in the Department of Behavioural Science and Health, Institute of Epidemiology and Healthcare, UCL. The project is funded by the Department of Health.

Contact for further information

Researcher: Dr Aradhna Kaushal | aradhna.kaushal.14@ucl.ac.uk | 020 7679 8254

Principal Researcher: Dr Christian von Wagner | c.wagner@ucl.ac.uk

UCL Data Protection Officer: Lee Shailer | l.shailer@ucl.ac.uk

Thank you for reading this information sheet and for considering to take part in this research study.

Kaushal A, *et al. BMJ Open* 2020; 10:e035264. doi: 10.1136/bmjopen-2019-035264

Consent form

Thank you for considering taking part in this research. If you have any questions arising from the Information explanation already given to you, please ask the researcher before you decide whether to join in.

I confirm that I understand that by ticking/initialling each box below I am consenting to this element of the study. I understand that it will be assumed that unticked/initialled boxes means that I DO NOT consent to that part of the study. I understand that by not giving consent for any one element that I may be deemed ineligible for the study.

	**Tick Box**
**1. I confirm that I have read and understood the information sheet for the study above. I have had an opportunity to consider the information and what will be expected of me. I have also had the opportunity to ask questions.**	
**2. I understand that my participation is voluntary and that I am free to withdraw at any time without giving a reason**	
**3. I understand that my data gathered in this study will be stored anonymously and securely. It will not be possible to identify me in any publications.**	
**4. I understand that the data will not be made available to any commercial organisations but is solely the responsibility of the researchers undertaking this study.**	
**5. I am aware of who I should contact if I wish to lodge a complaint.**	
**6. I voluntarily agree to take part in this study.**	
**7. I understand that information collected in this survey is stored on servers based in the USA.**	

Kaushal A, *et al. BMJ Open* 2020; 10:e035264. doi: 10.1136/bmjopen-2019-035264

Screening questions

First, we want to make sure you're a good fit for our study. Please answer the questions below so we can check.

1. ***Which of the following age groups are you in?***
   - 18-59 (exclude)
   - 60-64
   - 65-69
   - 70-74
   - 75+ (exclude)

2. ***Which of the following apply to you?***
   - I have been diagnosed with bowel cancer (exclude)
   - I have parts of my bowel been removed (exclude)
   - I have had a colonoscopy before (exclude)
   - None of the above

3. ***How many times have you been invited to take a faecal occult blood test (FOBt)? This is a screening test for bowel cancer which is sent in the post to men and women aged between 60 and 74.***
   - Never (Q6 + exclude)
   - 1
   - 2
   - 3 +

Image of FOBt test kit:

Redacted due to copyright restrictions

---NEXT PAGE---

4. ***How many times have you completed this test?***
   - 0 (Q7 + exclude)
   - 1
   - 2
   - 3 +
5. ***Have you ever had an abnormal test result i.e. you needed further tests?***
   - No
   - Yes (Q8 +exclude)
   - Don't remember

Kaushal A, *et al. BMJ Open* 2020; 10:e035264. doi: 10.1136/bmjopen-2019-035264

6. ***All adults aged between 60 and 75 should be invited for FOBt screening? Do you know why you have not been invited? (tick all that apply)***
    - I'm a new resident in the UK
    - I am not registered with a GP
    - I have recently moved home
    - I do not have a fixed address

    - Other (please specify)
7. ***What was the reason for not completing the test? (tick all that apply)***
    - I though completing a stool test is distasteful
    - I felt uncomfortable keeping samples of my stool on a test kit in the house
    - I felt uncomfortable sending a stool sample by post
    - I felt uncomfortable about someone looking at my stool sample
    - It is difficult to get round to doing the stool test
    - It was difficult to overcome the embarrassment related to the stool test
    - It was difficult to overcome the disgust related to the stool test

    - Other (please specify)

8. ***Can you remember what follow-up test you had?***
    - Colonoscopy
    - Felixible sigmoidoscopy
    - CT colonography

    - Other (please specify)

---RANDOMIZATION---

Test 1- Colonoscopy

Imagine that instead of a normal result, your test was abnormal. This means that traces of blood were found in your stool. In addition to your test result, you receive an invitation for a follow-up test called colonoscopy. This test looks for polyps and cancer in the large bowel and is performed by a healthcare professional at a hospital. The following table describes this test in detail. Please read this carefully.

Colonoscopy

Preparation	A healthcare professional will determine your eligibility to have a colonoscopy based on your health. Before a colonoscopy, you have to restrict your diet and take a strong laxative.
Test procedure	Before having a colonoscopy, you will be offered a sedative. This is usually given as an injection into a vein in your arm. A long flexible tube with a tiny camera on the end is inserted in your anus in order to examine your bowel. The procedure takes around 30 minutes to complete.<br>Illustration of colonoscopy procedure:<br>Redacted due to copyright restrictions
After the test	If you have the sedative, you will need to arrange for someone to take you home as you may feel drowsy. You will probably feel like resting, so you may want to book the whole day away from your commitments.
What if something is found?	Most polyps (small growths) that are found during the procedure can be removed straight away.
Risks	The procedure can be painful which is why you are offered a sedative. In a small number of cases the test can damage the bowel and cause bleeding (which may require surgery). The test can also miss cancer (about 5 out of 100).
What if I'm diagnosed with cancer	The main treatment for bowel cancer is surgery. In some cases, chemotherapy or radiotherapy may be offered. If the cancer is in a polyp that has been removed during colonoscopy, regular check-ups may be all that is needed. If bowel cancer is detected at the earliest stage, there is over 90% chance of survival.

--NEXT PAGE: COMPREHENSION CHECK—

Please read the following statements and indicate whether they are true or false.

Kaushal A, *et al. BMJ Open* 2020; 10:e035264. doi: 10.1136/bmjopen-2019-035264

9. ***Please read the following statements and select the one which best describes colonoscopy.***
   a) **A long flexible tube with a tiny camera tube is inserted in your anus in order to examine your bowel. Polyps are removed during the procedure. The procedure takes about 30 minutes.**
   b) A long flexible tube with a tiny camera tube is inserted in your anus in order to examine your bowel. If polyps are found, you will need a further examination. The procedure takes about 30 minutes.
   c) A long flexible tube with a tiny camera tube is inserted in your anus in order to examine your bowel. Polyps are removed during the procedure. The procedure takes about 60 minutes.

*If answer is incorrect (correct answers in bold), present information again and ask question again until answered correctly.

--NEXT PAGE--

10. ***a) Considering all the information presented above, would you take up the offer of this test?***
    - Definitely not [Send to page 13]
    - Probably not [Send to page 13]
    - Probably yes [Send to page 19]
    - Definitely yes [Send to page 19]

11. ***a) Please read each statement and select how strongly you agree or disagree with it (Strongly agree/Slightly agree/Slightly disagree/Strongly disagree)?***

    Emotional barriers
    - The preparation for the test (restricted diet and strong laxative) puts me off
    - The test looks like it would be uncomfortable
    - I would be embarrassed about taking the test
    - I would worry about the risks associated with the test
    - I would be afraid of getting an abnormal result
    - Doing the test would make me worry more about bowel cancer

    Practical barriers
    - I would not have time to do the test
    - I have other problems to worry about
    - It would be difficult to arrange transport to the hospital
    - I have other health problems that are more important
    - None of the above

    - Other (free text)

--NEXT PAGE--

Kaushal A, *et al. BMJ Open* 2020; 10:e035264. doi: 10.1136/bmjopen-2019-035264

Test 2

Imagine that instead of a normal result, your test was abnormal. This means that traces of blood were found in your stool. In addition to your test result, you receive an invitation for a follow-up test called capsule endoscopy. This test looks for polyps and cancer in the large bowel and is performed by a healthcare professional at a hospital. The following table describes this test in detail. Please read this carefully.

--INFORMATION ABOUT TEST—

Capsule endoscopy

Preparation	A healthcare professional will determine your eligibility to have a colon capsule based on your health. Before taking the colon capsule, you have to restrict your diet and take a strong laxative.
Test procedure	You swallow a capsule which contains a small camera. This allows for pictures to be taken of the inside of your bowel which are transmitted wirelessly to a data recorder worn around your waist. The camera is disposable and will pass naturally in your stool.<br>Image of capsule: Redacted due to copyright restrictions<br>Illustration of colon capsule in the bowel: Redacted due to copyright restrictions
After the test	It takes about 8 hours for the capsule to pass through your body. However, after taking the capsule you will be able to carry on with your normal activities.
What if something is found?	If polyps (small growths) are found during the procedure, you will be invited for a further examination, most likely colonoscopy.
Risks	In a small number of cases, the capsule may become stuck and may require surgical removal. This may cause abdominal pain, nausea and vomiting. The test can miss cancer (around 5 in 100).
What if I'm diagnosed with cancer	The main treatment for bowel cancer is surgery. In some cases, chemotherapy or radiotherapy may be offered. If the cancer is in a polyp that has been removed during colonoscopy, regular check-ups may be all that is needed. If bowel cancer is detected at the earliest stage, there is over 90% chance of survival.

--NEXT PAGE--

Please read the following statements and indicate whether they are true or false.

12. ***Please read the following statements and select the one which best describes capsule endoscopy.***
    a) The camera used in capsule endoscopy is disposable and will pass naturally in your stool after 8 hours. If polyps are found, they can be removed during the procedure.
    b) The camera used in capsule endoscopy is disposable and will pass naturally in your stool after 8 hours. If polyps are found during the procedure, you will be invited for a further examination, most likely colonoscopy.
    c) The camera used in capsule endoscopy is disposable and will pass naturally in your stool after 3 hours. If polyps are found during the procedure, you will be invited for a further examination, most likely colonoscopy.

    *If answer is incorrect (correct answers in bold), present information again and ask question again until answered correctly.

13. ***a) Considering all the information presented above, would you take up the offer of this test?***
    - Definitely not [Send to page 17]
    - Probably not [Send to page 17]
    - Probably yes [Send to page 19]
    - Definitely yes [Send to page 19]

14. ***a) Please read each statement and select how strongly you agree or disagree with it (Strongly agree/Slightly agree/Slightly disagree/Strongly disagree)?***

    Emotional barriers
    - The preparation for the test (restricted diet and strong laxative) puts me off
    - The test looks like it would be uncomfortable
    - I would be embarrassed about taking the test
    - I would worry about the risks associated with the test
    - I would be afraid of getting an abnormal result
    - Doing the test would make me worry more about bowel cancer

    Practical barriers
    - I would not have time to do the test
    - I have other problems to worry about
    - It would be difficult to arrange transport to the hospital
    - I have other health problems that are more important
    - None of the above

    - Other (free text)

--NEXT PAGE--

Kaushal A, *et al. BMJ Open* 2020; 10:e035264. doi: 10.1136/bmjopen-2019-035264

Test 3

Imagine that instead of a normal result, your test was abnormal. This means that traces of blood were found in your stool. In addition to your test result, you receive an invitation for a follow-up test called CT colonography. This test looks for polyps and cancer in the large bowel and is performed by a healthcare professional at a hospital. The following table describes this test in detail. Please read this carefully.

--INFORMATION ABOUT TEST—

CT colonography

Preparation	Before having the scan, you have to restrict your diet and take a laxative.
Test procedure	Before having a CT colonography, you may be given an injection of a medicine to relax the bowel. A radiographer then puts a small tube a few centimetres long into your back passage (rectum) to pump carbon dioxide or air inside. This opens the bowel, helping to get clear scans of the inside of your bowel. X-rays are used to make images of your bowel. You may be given an injection of a contrast medium (x-ray dye) to help collect more information from the scan. The procedure takes around 20 minutes to complete.<br>Image of CT scanner: Redacted due to copyright restrictions<br>Image of CT scans: Redacted due to copyright restrictions
After the test	You may wish to rest at home for the rest of the day, although most people are able to resume their normal daily activities.
What if something is found?	If polyps (small growths) are found during the procedure, you will be invited for a further examination, most likely colonoscopy.
Risks	The procedure may cause some discomfort and you may feel faint. In a very small number of cases the test can damage the bowel which may require surgery. There is also a very small risk that you will have a reaction to the injections. You will be exposed to a small amount of radiation. The test can miss cancer (around 5 in 100).
What if I'm diagnosed with cancer	The main treatment for bowel cancer is surgery. In some cases, chemotherapy or radiotherapy may be offered. If the cancer is in a polyp that has been removed during colonoscopy, regular check-ups may be all that is needed. If bowel cancer is detected at the earliest stage, there is over 90% chance of survival.

--NEXT PAGE--

Please read the following statements and indicate whether they are true or false.

Kaushal A, *et al. BMJ Open* 2020; 10:e035264. doi: 10.1136/bmjopen-2019-035264

15. ***Please read the following statements and select the one which best describes CT colonography.***
    a) X-rays are used to make images of your bowel. If polyps are found, they can be removed during the procedure. The procedure takes about 20 minutes to complete.
    b) X-rays are used to make images of your bowel. If polyps are found during the procedure, you will be invited for a further examination, most likely colonoscopy. The procedure takes about 20 minutes to complete.
    c) X-rays are used to make images of your bowel. If polyps are found during the procedure, you will be invited for a further examination, most likely colonoscopy. The procedure takes about 60 minutes to complete.

    *If answer is incorrect (correct answers in bold), present information again and ask question again until answered correctly.

16. ***a) Considering all the information presented above, would you take up the offer of this test?***
    - Definitely not [Send to page 15]
    - Probably not [Send to page 15]
    - Probably yes [Send to page 19]
    - Definitely yes [Send to page 19]

17. ***a) Please read each statement and select how strongly you agree or disagree with it (Strongly agree/Slightly agree/Slightly disagree/Strongly disagree)?***

    Emotional barriers
    - The preparation for the test (restricted diet and strong laxative) puts me off
    - The test looks like it would be uncomfortable
    - I would be embarrassed about taking the test
    - I would worry about the risks associated with the test
    - I would be afraid of getting an abnormal result
    - Doing the test would make me worry more about bowel cancer

    Practical barriers

    - I would not have time to do the test
    - I have other problems to worry about
    - It would be difficult to arrange transport to the hospital
    - I have other health problems that are more important
    - None of the above

    - Other (free text)

--NEXT PAGE--

Kaushal A, *et al. BMJ Open* 2020; 10:e035264. doi: 10.1136/bmjopen-2019-035264

Colonoscopy decliners

You said that you probably or definitely wouldn't have a colonoscopy. The following table describes two other tests that you could be invited to instead. Please read this carefully.

	CT colonography	***Capsule endoscopy***
Preparation	Before having the scan, you have to restrict your diet and take a laxative.	A healthcare professional will determine your eligibility to have a colon capsule based on your health. Before taking the colon capsule, you have to restrict your diet and take a strong laxative.
Test procedure	Before having a CT colonography, you may be given an injection of a medicine to relax the bowel. A radiographer then puts a small tube a few centimetres long into your back passage (rectum) to pump carbon dioxide or air inside. This opens the bowel, helping to get clear scans of the inside of your bowel. X-rays are used to make images of your bowel. You may be given an injection of a contrast medium (x-ray dye) to help collect more information from the scan. The procedure takes around 20 minutes to complete.<br>Image of CT scanner: Redacted due to copyright restrictions<br>Image of CT scans: Redacted due to copyright restrictions	You swallow a capsule which contains a small camera. This allows for pictures to be taken of the inside of your bowel which are transmitted wirelessly to a data recorder worn around your waist. The camera is disposable and will pass naturally in your stool.<br>Image of capsule: Redacted due to copyright restrictions<br>Illustration of colon capsule in the bowel: Redacted due to copyright restrictions
After the test	You may wish to rest at home for the rest of the day, although most people are able to resume their normal daily activities.	It takes about 8 hours for the capsule to pass through your body. However, after taking the capsule you will be able to carry on with your normal activities.
What if something is found?	If polyps (small growths) are found during the procedure, you will be invited for a further examination, most likely colonoscopy.	If polyps (small growths) are found during the procedure, you will be invited for a further examination, most likely colonoscopy.
Risks	The procedure may cause some discomfort and you may feel faint. In a very small number of cases the test can damage the bowel which may require surgery. There is also a very small risk that you will have a reaction to the injections. You will be exposed to a small amount of radiation. The test can miss cancer (around 5 in 100).	In a small number of cases, the capsule may become stuck and may require surgical removal. This may cause abdominal pain, nausea and vomiting. The test can miss cancer (around 5 in 100).
What if I'm diagnosed with cancer	The main treatment for bowel cancer is surgery. In some cases, chemotherapy or radiotherapy may be offered. If the cancer is in a polyp that has been removed during colonoscopy, regular check-ups may be all that is needed. If bowel cancer is detected at the earliest stage, there is over 90% chance of survival.	

Kaushal A, *et al. BMJ Open* 2020; 10:e035264. doi: 10.1136/bmjopen-2019-035264

18. ***Considering all the information presented above, would you take up the offer of CT colonography?***
    - Definitely not
    - Probably not
    - Probably yes
    - Probably yes
19. ***Considering all the information presented above, would you take up the offer of capsule endoscopy?***
    - Definitely not
    - Probably not
    - Probably yes
    - Probably yes
20. ***Which of the two tests would you prefer to have? (choose one)***
    - CT colonography
    - Capsule endoscopy

Kaushal A, *et al. BMJ Open* 2020; 10:e035264. doi: 10.1136/bmjopen-2019-035264

CT colonography decliners

You said that you probably or definitely wouldn't have a CT colonography. The following table describes two other tests that you could be invited to instead. Please read this carefully.

	**Colonoscopy**	**Capsule endoscopy**
Preparation	A healthcare professional will determine your eligibility to have a colonoscopy based on your health. Before a colonoscopy, you have to restrict your diet and take a strong laxative.	A healthcare professional will determine your eligibility to have a colon capsule based on your health. Before taking the colon capsule, you have to restrict your diet and take a strong laxative.
Test procedure	Before having a colonoscopy, you will be offered a sedative. This is usually given as an injection into a vein in your arm. A long flexible tube with a tiny camera on the end is inserted in your anus in order to examine your bowel. The procedure takes around 30 minutes to complete.<br><br>Illustration of colonoscopy procedure:<br>Redacted due to copyright restrictions	You swallow a capsule which contains a small camera. This allows for pictures to be taken of the inside of your bowel which are transmitted wirelessly to a data recorder worn around your waist. The camera is disposable and will pass naturally in your stool.<br><br>Image of capsule:<br>Redacted due to copyright restrictions<br><br>Illustration of colon capsule in the bowel:<br>Redacted due to copyright restrictions
After the test	If you have the sedative, you will need to arrange for someone to take you home as you may feel drowsy. You will probably feel like resting, so you may want to book the whole day away from your commitments.	It takes about 8 hours for the capsule to pass through your body. However, after taking the capsule you will be able to carry on with your normal activities.
What if something is found?	Most polyps (small growths) that are found during the procedure can be removed straight away.	If polyps (small growths) are found during the procedure, you will be invited for a further examination, most likely colonoscopy.
Risks	The procedure can be painful which is why you are offered a sedative. In a small number of cases the test can damage the bowel and cause bleeding (which may require surgery). The test can also miss cancer (about 5 out of 100).	In a small number of cases, the capsule may become stuck and may require surgical removal. This may cause abdominal pain, nausea and vomiting. The test can miss cancer (around 5 in 100).
What if I'm diagnosed with cancer	The main treatment for bowel cancer is surgery. In some cases, chemotherapy or radiotherapy may be offered. If the cancer is in a polyp that has been removed during colonoscopy, regular check-ups may be all that is needed. If bowel cancer is detected at the earliest stage, there is over 90% chance of survival.	

21. *Considering all the information presented above, would you take up the offer of colonoscopy?*

- Definitely not

- Probably not
- Probably yes
- Probably yes

22. Considering all the information presented above, would you take up the offer of capsule endoscopy?

- Definitely not
- Probably not
- Probably yes
- Probably yes

23. Which of the two tests would you prefer to have? (choose one)

- Colonoscopy
- Capsule endoscopy

Kaushal A, *et al. BMJ Open* 2020; 10:e035264. doi: 10.1136/bmjopen-2019-035264

Capsule endoscopy decliners

You said that you probably or definitely wouldn't have capsule endoscopy. The following table describes two other tests that you could be invited to instead. Please read this carefully.

	**Colonoscopy**	**CT colonography**
Preparation	A healthcare professional will determine your eligibility to have a colonoscopy based on your health. Before a colonoscopy, you have to restrict your diet and take a strong laxative.	Before having the scan, you have to restrict your diet and take a laxative.
Test procedure	Before having a colonoscopy, you will be offered a sedative. This is usually given as an injection into a vein in your arm. A long flexible tube with a tiny camera on the end is inserted in your anus in order to examine your bowel. The procedure takes around 30 minutes to complete.<br><br>Illustration of colonoscopy procedure:<br>Redacted due to copyright restrictions	Before having a CT colonography, you may be given an injection of a medicine to relax the bowel. A radiographer then puts a small tube a few centimetres long into your back passage (rectum) to pump carbon dioxide or air inside. This opens the bowel, helping to get clear scans of the inside of your bowel. X-rays are used to make images of your bowel. You may be given an injection of a contrast medium (x-ray dye) to help collect more information from the scan. The procedure takes around 20 minutes to complete.<br><br>Image of CT scanner:<br>Redacted due to copyright restrictions<br><br>Image of CT scans:<br>Redacted due to copyright restrictions
After the test	If you have the sedative, you will need to arrange for someone to take you home as you may feel drowsy. You will probably feel like resting, so you may want to book the whole day away from your commitments.	You may wish to rest at home for the rest of the day, although most people are able to resume their normal daily activities.
What if something is found?	Most polyps (small growths) that are found during the procedure can be removed straight away.	If polyps (small growths) are found during the procedure, you will be invited for a further examination, most likely colonoscopy.
Risks	The procedure can be painful which is why you are offered a sedative. In a small number of cases the test can damage the bowel and cause bleeding (which may require surgery). The test can also miss cancer (about 5 out of 100).	The procedure may cause some discomfort and you may feel faint. In a very small number of cases the test can damage the bowel which may require surgery. There is also a very small risk that you will have a reaction to the injections. You will be exposed to a small amount of radiation. The test can miss cancer (around 5 in 100).
What if I'm diagnosed with cancer	The main treatment for bowel cancer is surgery. In some cases, chemotherapy or radiotherapy may be offered. If the cancer is in a polyp that has been removed during colonoscopy, regular check-ups may be all that is needed. If bowel cancer is detected at the earliest stage, there is over 90% chance of survival.	

Kaushal A, *et al. BMJ Open* 2020; 10:e035264. doi: 10.1136/bmjopen-2019-035264

25. ***Considering all the information presented above, would you take up the offer of colonoscopy?***
    - Definitely not
    - Probably not
    - Probably yes
    - Probably yes
26. ***Considering all the information presented above, would you take up the offer of CT colonography?***
    - Definitely not
    - Probably not
    - Probably yes
    - Probably yes
27. ***Which of the two tests would you prefer to have? (choose one)***
    - Colonoscopy
    - CT colonography

Kaushal A, *et al. BMJ Open* 2020; 10:e035264. doi: 10.1136/bmjopen-2019-035264

Socio- demographic questions

28. Are you…?

- Male
- Female

29. Are you currently…?

- Employed full-time
- Employed part-time
- Self-employed
- Unemployed
- Full-time homemaker
- Retired
- Student
- Disabled or too ill to work

30. Does your household have a car of van?

- No
- Yes

31. Which best describes your living arrangement?

- Rent privately or from council, local authority or housing association
- Own your home / have a mortgage

32. Which of the following describes your ethnic group?

- White British
- Other White background
- Black background
- Asian background
- Mixed background
- Any other

33. What is your highest educational qualification?

- No qualifications
- GCSE/O Level, vocational level 2 and equivalents
- A levels, vocational level 3 and equivalents
- Higher Education & professional/vocational equivalents

34. Which of the following numbers represents the biggest risk of getting a disease?

- 1/10
- 1/100
- 1/1000
- I don't know

Kaushal A, *et al. BMJ Open* 2020; 10:e035264. doi: 10.1136/bmjopen-2019-035264
