# Supplementary material for: Preferences for different diagnostic modalities to follow up abnormal colorectal cancer screening results: a hypothetical vignette study
Source: BMJ Open. 2020 Jul 26;10(7):e035264. doi: 10.1136/bmjopen-2019-035264 (PMC7383951; doi:10.1136/bmjopen-2019-035264)
Supplement: Supplementary data [file bmjopen-2019-035264supp002.pdf]

Table 1. Intention to have the test for colonoscopy, CT colonography, and capsule endoscopy n (%).

N=953	Colonoscopy	Capsule endoscopy	CT colonography
Definitely not	4 (1.3)	6 (1.7)	2 (0.7)
Probably not	21 (6.9)	17 (4.9)	8 (2.7)
Yes, probably	114 (37.4)	123 (35.6)	115 (38.1)
Yes, definitely	166 (54.4)	200 (57.8)	177 (58.6)

Table 2.Emotional barriers to each test n(%)

N=953	Colonoscopy	Capsule endoscopy	CT colonography
**Off-putting**			
Strongly disagree	106 (34.8)	134 (38.7)	122 (40.4)
Slightly disagree	94 (30.8)	116 (33.5)	103 (34.1)
Slightly agree	83 (27.2)	84 (24.3)	67 (22.2)
Strongly agree	22 (7.2)	12 (3.5)	10 (3.3)
**Uncomfortable**			
Strongly disagree	14 (4.6)	87 (25.1)	27 (8.9)
Slightly disagree	32 (10.5)	127 (36.7)	51 (16.9)
Slightly agree	183 (60.0)	119 (34.4)	188 (62.3)
Strongly agree	76 (24.9)	13 (3.8)	36 (11.9)
**Embarrassing**			
Strongly disagree	115 (37.7)	201 (58.1)	135 (44.7)
Slightly disagree	75 (24.6)	89 (25.7)	77 (25.5)
Slightly agree	92 (30.2)	51 (14.7)	76 (25.2)
Strongly agree	23 (7.5)	5 (1.5)	14 (4.6)
**Worry about risks**			
Strongly disagree	82 (26.9)	110 (31.8)	103 (34.1)
Slightly disagree	95 (31.2)	121 (35.0)	95 (31.5)
Slightly agree	111 (36.4)	107 (30.9)	92 (30.5)
Strongly agree	17 (5.6)	8 (2.3)	12 (4.0)
**Afraid of results**			
Strongly disagree	64 (21.0)	87 (25.1)	68 (22.5)
Slightly disagree	54 (17.7)	73 (21.1)	55 (18.2)
Slightly agree	140 (45.9)	154 (44.5)	146 (48.3)
Strongly agree	47 (15.4)	32 (9.3)	33 (10.9)
**Worry about cancer**			
Strongly disagree	87 (28.5)	126 (36.4)	86 (28.5)
Slightly disagree	97 (31.8)	99 (28.6)	85 (28.2)
Slightly agree	94 (30.8)	108 (31.2)	112 (37.1)
Strongly agree	27 (8.9)	13 (3.8)	19 (6.3)

Table 3.Practical barriers to each test n(%)

N=953	Colonoscopy	Capsule endoscopy	CT colonography
**No time**			
Strongly disagree	226 (74.1)	285 (82.4)	246 (81.5)
Slightly disagree	61 (20.0)	55 (15.9)	48 (15.9)
Slightly agree	14 (4.6)	6 (1.7)	6 (2.0)
Strongly agree	4 (1.3)	0 (0.0)	2 (0.7)
**Other problems**			
Strongly disagree	140 (45.9)	182 (52.6)	144 (47.7)
Slightly disagree	88 (28.9)	94 (27.2)	94 (31.1)
Slightly agree	61 (20.0)	61 (17.6)	49 (16.2)
Strongly agree	16 (5.3)	9 (2.6)	15 (5.0)
**Difficulties with transport**			
Strongly disagree	188 (61.6)	239 (69.1)	210 (69.5)
Slightly disagree	67 (22.0)	63 (18.2)	58 (19.2)
Slightly agree	39 (12.8)	39 (11.3)	27 (8.9)
Strongly agree	11 (3.6)	5 (1.5)	7 (2.3)
**Health problems**			
Strongly disagree	183 (60.0)	218 (63.0)	209 (68.9)
Slightly disagree	85 (27.9)	96 (27.8)	71 (23.5)
Slightly agree	27 (8.9)	26 (7.5)	14 (4.6)
Strongly agree	10 (3.3)	6 (1.7)	9 (3.0)
