# Supplementary material for: Preferences for different diagnostic modalities to follow up abnormal colorectal cancer screening results: a hypothetical vignette study
Source: BMJ Open. 2020 Jul 26;10(7):e035264. doi: 10.1136/bmjopen-2019-035264 (PMC7383951; doi:10.1136/bmjopen-2019-035264)
Supplement: Supplementary data [file bmjopen-2019-035264supp003.pdf]

```
  Thursday September 5 11:12:06 2019   Page 1

                                              ___  ____  ____  ____  ____(R)
                                             /__    /   ____/   /   ____/
                                            ___/   /   /___/   /   /___/
                                              Statistics/Data Analysis

      name:  <unnamed>
       log:  S:\FPHS_BSH_CancerComm\QQ PROJECTS 2012-17\BOWEL SCREENING PROJECT\Alternatives to
  log type:  smcl
 opened on:   5 Sep 2019, 11:08:18

1 . use "S:\FPHS_BSH_CancerComm\QQ PROJECTS 2012-17\BOWEL SCREENING PROJECT\Alternatives to Colon

2 .
3 . //Intention and BAME interaction//
4 . logit int2 i.cond##i.ethnicity i.cage i.female i.idep i.paid i.num if comp==1, or

note: 1.cond#1.ethnicity != 0 predicts success perfectly
      1.cond#1.ethnicity dropped and 4 obs not used

note: 3.cond#1.ethnicity omitted because of collinearity
Iteration 0:   log likelihood = -218.29837
Iteration 1:   log likelihood = -215.64434
Iteration 2:   log likelihood = -206.51414
Iteration 3:   log likelihood = -199.21258
Iteration 4:   log likelihood = -197.68211
Iteration 5:   log likelihood = -197.37916
Iteration 6:   log likelihood = -197.37691
Iteration 7:   log likelihood = -197.37691

Logistic regression                             Number of obs     =        949
                                                LR chi2(11)       =      41.84
                                                Prob > chi2       =     0.0000
Log likelihood = -197.37691                     Pseudo R2         =     0.0958
```

int2	Odds Ratio	Std. Err.	z	P>|z|	[95% Conf.	Interval]
**cond**						
Capsule	1.440134	.4558544	1.15	0.249	.7744031	2.678175
CT colonography	3.164895	1.327974	2.75	0.006	1.390591	7.203097
**ethnicity**						
BAME	.0257632	.0289846	-3.25	0.001	.0028403	.2336873
**cond#ethnicity**						
Colonoscopy#BAME	1	(empty)				
Capsule#BAME	1.525905	2.263561	0.28	0.776	.0833368	27.93948
CT colonography#BAME	1	(omitted)				
**cage**						
65-69	.7060353	.2438926	-1.01	0.314	.3587465	1.389521
70-74	1.402867	.5943763	0.80	0.424	.6114676	3.218544
**female**						
Yes	1.06484	.3075379	0.22	0.828	.6045717	1.875517
**idep**						
1 marker	.4684608	.148785	-2.39	0.017	.2513786	.8730079
2-3 markers	.3456216	.1480694	-2.48	0.013	.1492566	.8003283
**paid**						
Yes	.7472369	.2478844	-0.88	0.380	.3900185	1.431632
**num**						
Correct	1.487324	.4351085	1.36	0.175	.8382839	2.638882
_cons	14.26229	6.135926	6.18	0.000	6.137408	33.14312

Note: **_cons** estimates baseline odds.

```
  Thursday September 5 11:12:08 2019   Page 2

 5 .
 6 . //Intention and deprivation interaction//
 7 . logit int2 i.cond##i.idep  i.cage i.female i.ethnicity i.paid i.num if comp==1, or

  Iteration 0:   log likelihood = -218.55008
  Iteration 1:   log likelihood = -203.06951
  Iteration 2:   log likelihood = -199.19849
  Iteration 3:   log likelihood = -199.10343
  Iteration 4:   log likelihood = -199.10307
  Iteration 5:   log likelihood = -199.10307

  Logistic regression                             Number of obs     =        953
                                                  LR chi2(14)       =      38.89
                                                  Prob > chi2       =     0.0004
  Log likelihood = -199.10307                     Pseudo R2         =     0.0890
```

int2	Odds Ratio	Std. Err.	z	P>|z|	[95% Conf.	Interval]
cond						
Capsule	1.740958	.7370709	1.31	0.190	.7593019	3.99174
CT colonography	3.305797	1.748944	2.26	0.024	1.172046	9.324114
idep						
1 marker	.5428541	.2653282	-1.25	0.211	.208279	1.414884
2-3 markers	.8346543	.6618163	-0.23	0.820	.1764289	3.948604
cond#idep						
Capsule#1 marker	.7549797	.5310209	-0.40	0.689	.1902111	2.99664
Capsule#2-3 markers	.1872543	.1874193	-1.67	0.094	.0263318	1.33163
CT colonography#1 marker	.6383686	.5497961	-0.52	0.602	.1180246	3.452792
CT colonography#2-3 markers	.4718537	.6540561	-0.54	0.588	.0311833	7.139919
cage						
65-69	.6643002	.2293366	-1.18	0.236	.3376788	1.306848
70-74	1.385225	.5810532	0.78	0.437	.6087953	3.151879
female						
Yes	1.135806	.3270864	0.44	0.658	.6459178	1.997243
ethnicity						
BAME	.0868887	.0536264	-3.96	0.000	.0259188	.2912809
paid						
Yes	.8567131	.2863368	-0.46	0.644	.4449803	1.649415
num						
Correct	1.533339	.44838	1.46	0.144	.8644292	2.719864
_cons	12.76679	5.550371	5.86	0.000	5.44529	29.93245

```
  Note: _cons estimates baseline odds.

 8 .
 9 . //Off-putting and gender interaction//
10 . logit datt1 i.cond##i.female i.cage i.ethnicity i.idep i.paid i.num if comp==1, or

  Iteration 0:   log likelihood = -575.64795
  Iteration 1:   log likelihood = -567.00146
  Iteration 2:   log likelihood = -566.95168
  Iteration 3:   log likelihood = -566.95168

  Logistic regression                             Number of obs     =        954
                                                  LR chi2(12)       =      17.39
                                                  Prob > chi2       =     0.1354
  Log likelihood = -566.95168                     Pseudo R2         =     0.0151
```

Thursday September 5 11:12:08 2019 Page 3

datt1	Odds Ratio	Std. Err.	z	P>|z|	[95% Conf.	Interval]
cond						
Capsule	.5994014	.1453158	-2.11	0.035	.3726978	.9640035
CT colonography	.602084	.1528489	-2.00	0.046	.3660713	.9902584
female						
Yes	1.147654	.2795688	0.57	0.572	.7119644	1.849967
cond#female						
Capsule#Yes	1.561034	.5367613	1.30	0.195	.7956584	3.062653
CT colonography#Yes	1.193689	.4305356	0.49	0.624	.5886849	2.42047
cage						
65-69	.9965692	.1806268	-0.02	0.985	.6985996	1.42163
70-74	1.047513	.2039252	0.24	0.812	.7152387	1.534149
ethnicity						
BAME	2.271933	1.290318	1.44	0.148	.7463898	6.91553
idep						
1 marker	1.061183	.1891052	0.33	0.739	.7483492	1.504792
2-3 markers	1.403437	.3834878	1.24	0.215	.8214909	2.397634
paid						
Yes	.9865023	.1755985	-0.08	0.939	.6959585	1.39834
num						
Correct	1.039599	.1512154	0.27	0.789	.7817247	1.38254
_cons	.4533808	.1097497	-3.27	0.001	.2821064	.7286403

Note: **_cons** estimates baseline odds.

```
11 .
12 . //Embarrasing and gender interaction//
13 . logit datt3 i.cond##i.female i.cage i.ethnicity i.idep i.paid i.num if comp==1, or

Iteration 0:   log likelihood = -559.79953
Iteration 1:   log likelihood = -526.78936
Iteration 2:   log likelihood = -526.08874
Iteration 3:   log likelihood = -526.08791
Iteration 4:   log likelihood = -526.08791

Logistic regression                             Number of obs     =        954
                                                LR chi2(12)       =      67.42
                                                Prob > chi2       =     0.0000
Log likelihood = -526.08791                     Pseudo R2         =     0.0602
```

datt3	Odds Ratio	Std. Err.	z	P>|z|	[95% Conf.	Interval]
cond						
Capsule	.393843	.1049377	-3.50	0.000	.2336277	.6639295
CT colonography	.6117832	.158551	-1.90	0.058	.3681276	1.016709
female						
Yes	1.802571	.4375261	2.43	0.015	1.120175	2.900674
cond#female						
Capsule#Yes	.6950697	.2652284	-0.95	0.340	.32902	1.468366
CT colonography#Yes	1.382337	.4910231	0.91	0.362	.6890595	2.773136
cage						
65-69	.8078209	.1503881	-1.15	0.252	.5608556	1.163534
70-74	.889448	.1795823	-0.58	0.562	.5987705	1.321237
ethnicity						
BAME	1.155396	.7401264	0.23	0.822	.3292044	4.055045
idep						
1 marker	.9886713	.1860287	-0.06	0.952	.6837378	1.429599

Thursday September 5 11:12:08 2019 Page 4

2-3 markers	**1.281844**	**.3718801**	**0.86**	**0.392**	**.7259228**	**2.263498**
paid						
Yes	**1.439032**	**.2589492**	**2.02**	**0.043**	**1.011346**	**2.047583**
num						
Correct	**1.112985**	**.1693064**	**0.70**	**0.482**	**.8260481**	**1.499593**
_cons	**.420774**	**.1040708**	**-3.50**	**0.000**	**.2591312**	**.6832476**

Note: **_cons** estimates baseline odds.

```
14 .
15 . //Worry about test risk and gender interaction//
16 . logit datt4 i.cond##i.female i.cage i.ethnicity i.idep i.paid i.num if comp==1, or

Iteration 0:   log likelihood = -625.38047
Iteration 1:   log likelihood = -610.79631
Iteration 2:   log likelihood = -610.75012
Iteration 3:   log likelihood = -610.75012

Logistic regression                             Number of obs     =        954
                                                LR chi2(12)       =      29.26
                                                Prob > chi2       =     0.0036
Log likelihood = -610.75012                     Pseudo R2         =     0.0234
```

datt4	Odds Ratio	Std. Err.	z	P>|z|	[95% Conf.	Interval]
cond						
Capsule	**.8625152**	**.1965071**	**-0.65**	**0.516**	**.5518706**	**1.34802**
CT colonography	**.6402238**	**.1565886**	**-1.82**	**0.068**	**.396407**	**1.034004**
female						
Yes	**1.574237**	**.372587**	**1.92**	**0.055**	**.9899417**	**2.503402**
cond#female						
Capsule#Yes	**.626443**	**.2078761**	**-1.41**	**0.159**	**.3269057**	**1.20044**
CT colonography#Yes	**1.312932**	**.4499751**	**0.79**	**0.427**	**.6706774**	**2.570221**
cage						
65-69	**.8532505**	**.1461122**	**-0.93**	**0.354**	**.6099795**	**1.193542**
70-74	**.8931162**	**.1645699**	**-0.61**	**0.540**	**.6223884**	**1.281606**
ethnicity						
BAME	**1.94679**	**1.10931**	**1.17**	**0.242**	**.6372233**	**5.947667**
idep						
1 marker	**1.494679**	**.2483308**	**2.42**	**0.016**	**1.079261**	**2.069995**
2-3 markers	**1.271042**	**.3415635**	**0.89**	**0.372**	**.7506177**	**2.15229**
paid						
Yes	**1.121261**	**.1875352**	**0.68**	**0.494**	**.8078669**	**1.556228**
num						
Correct	**1.065082**	**.1471823**	**0.46**	**0.648**	**.8123747**	**1.396398**
_cons	**.532697**	**.1239152**	**-2.71**	**0.007**	**.3376564**	**.8403991**

Note: **_cons** estimates baseline odds.

17 .

```
  Thursday September 5 11:12:09 2019   Page 5

18 . //Worry about test risk and deprivation interaction//
19 . logit datt4 i.cond##i.idep i.cage i.female i.ethnicity i.paid i.num if comp==1, or

   Iteration 0:   log likelihood = -625.38047
   Iteration 1:   log likelihood = -609.00743
   Iteration 2:   log likelihood = -608.97148
   Iteration 3:   log likelihood = -608.97148

   Logistic regression                             Number of obs     =        954
                                                   LR chi2(14)       =      32.82
                                                   Prob > chi2       =     0.0031
   Log likelihood = -608.97148                     Pseudo R2         =     0.0262
```

datt4	Odds Ratio	Std. Err.	z	P>|z|	[95% Conf.	Interval]
**cond**						
Capsule	.8545529	.1688906	-0.80	0.426	.5801105	1.25883
CT colonography	.8738928	.1757055	-0.67	0.503	.5892709	1.295989
**idep**						
1 marker	2.773085	.8485567	3.33	0.001	1.522291	5.051596
2-3 markers	1.35632	.5985531	0.69	0.490	.5711117	3.221092
**cond#idep**						
Capsule#1 marker	.3593903	.1484572	-2.48	0.013	.159938	.8075719
Capsule#2-3 markers	1.287068	.7999347	0.41	0.685	.3806846	4.351488
CT colonography#1 marker	.4862063	.2070739	-1.69	0.090	.2110067	1.120327
CT colonography#2-3 markers	.5373902	.3794284	-0.88	0.379	.1346767	2.144307
**cage**						
65-69	.8475212	.1459977	-0.96	0.337	.6046707	1.187906
70-74	.8790054	.1626208	-0.70	0.486	.6116663	1.263189
**female**						
Yes	1.454996	.2032422	2.68	0.007	1.106525	1.91321
**ethnicity**						
BAME	2.084474	1.191957	1.28	0.199	.6796058	6.39346
**paid**						
Yes	1.107485	.1857581	0.61	0.543	.7971979	1.538543
**num**						
Correct	1.074747	.1491767	0.52	0.604	.8187623	1.410764
_cons	.4918955	.1081869	-3.23	0.001	.3196396	.7569813

Note: **_cons** estimates baseline odds.

```
20 .
21 . //Afraid of results risk and age group interaction//
22 . logit datt5 i.cond##i.cage i.female i.ethnicity i.idep i.paid i.num if comp==1, or

   Iteration 0:   log likelihood = -649.10164
   Iteration 1:   log likelihood =   -626.968
   Iteration 2:   log likelihood = -626.80181
   Iteration 3:   log likelihood = -626.80112
   Iteration 4:   log likelihood = -626.80112

   Logistic regression                             Number of obs     =        954
                                                   LR chi2(14)       =      44.60
                                                   Prob > chi2       =     0.0000
   Log likelihood = -626.80112                     Pseudo R2         =     0.0344
```

Thursday September 5 11:12:09 2019 Page 6

datt5	Odds Ratio	Std. Err.	z	P>|z|	[95% Conf.	Interval]
cond						
Capsule	.9487913	.2971592	-0.17	0.867	.5135425	1.752932
CT colonography	.8040911	.2395326	-0.73	0.464	.4484756	1.441689
cage						
65-69	.6737809	.1962447	-1.36	0.175	.3807139	1.192446
70-74	.7619691	.2441478	-0.85	0.396	.4066287	1.427831
cond#cage						
Capsule#65-69	.8464529	.3448479	-0.41	0.682	.3809082	1.880985
Capsule#70-74	.618933	.2636364	-1.13	0.260	.2685788	1.426315
CT colonography#65-69	1.124773	.4530285	0.29	0.770	.5107682	2.476887
CT colonography#70-74	1.360613	.5960542	0.70	0.482	.5765568	3.210902
female						
Yes	1.579452	.217962	3.31	0.001	1.205152	2.070003
ethnicity						
BAME	3.903997	3.066424	1.73	0.083	.8373884	18.20087
idep						
1 marker	1.369841	.2341368	1.84	0.066	.9798976	1.91496
2-3 markers	.6881451	.1825012	-1.41	0.159	.4091994	1.157244
paid						
Yes	1.1609	.1946665	0.89	0.374	.835719	1.61261
num						
Correct	1.318004	.1794388	2.03	0.043	1.009323	1.721087
_cons	1.307052	.3353875	1.04	0.297	.7904531	2.161274

Note: **_cons** estimates baseline odds.

```
23 .
24 . //Afraid of results risk and gender interaction//
25 . logit datt5 i.cond##i.female i.cage i.ethnicity i.idep i.paid i.num if comp==1, or

Iteration 0:   log likelihood = -649.10164
Iteration 1:   log likelihood = -628.77429
Iteration 2:   log likelihood = -628.62076
Iteration 3:   log likelihood = -628.62007
Iteration 4:   log likelihood = -628.62007

Logistic regression                             Number of obs     =        954
                                                LR chi2(12)       =      40.96
                                                Prob > chi2       =     0.0000
Log likelihood = -628.62007                     Pseudo R2         =     0.0316
```

datt5	Odds Ratio	Std. Err.	z	P>|z|	[95% Conf.	Interval]
cond						
Capsule	.7984076	.1756849	-1.02	0.306	.5187078	1.228928
CT colonography	.9635103	.2206762	-0.16	0.871	.6150389	1.50942
female						
Yes	1.706078	.413164	2.21	0.027	1.061358	2.742432
cond#female						
Capsule#Yes	.8715473	.286892	-0.42	0.676	.4571896	1.661444
CT colonography#Yes	.8956024	.3066038	-0.32	0.747	.457838	1.751938
cage						
65-69	.6722562	.1145439	-2.33	0.020	.4813943	.9387906
70-74	.7060271	.1287507	-1.91	0.056	.4938519	1.00936
ethnicity						
BAME	3.943815	3.079102	1.76	0.079	.8537837	18.21735

Thursday September 5 11:12:10 2019 Page 7

idep						
1 marker	**1.355455**	**.2308021**	**1.79**	**0.074**	**.9708356**	**1.892452**
2-3 markers	**.6852833**	**.1808773**	**-1.43**	**0.152**	**.408507**	**1.149584**
paid						
Yes	**1.188009**	**.1987038**	**1.03**	**0.303**	**.8559524**	**1.648883**
num						
Correct	**1.321628**	**.1794319**	**2.05**	**0.040**	**1.01285**	**1.72454**
_cons	**1.28226**	**.2930399**	**1.09**	**0.277**	**.8193087**	**2.006802**

Note: **_cons** estimates baseline odds.

```
26 .
27 . //Worry about cancer and gender interaction//
28 . logit datt6 i.cond##i.female i.cage i.ethnicity i.idep i.paid i.num if comp==1, or

Iteration 0:   log likelihood = -638.84517
Iteration 1:   log likelihood = -629.17662
Iteration 2:   log likelihood = -629.16658
Iteration 3:   log likelihood = -629.16658

Logistic regression                             Number of obs     =        954
                                                LR chi2(12)       =      19.36
                                                Prob > chi2       =     0.0803
Log likelihood = -629.16658                     Pseudo R2         =     0.0152
```

datt6	Odds Ratio	Std. Err.	z	P>|z|	[95% Conf.	Interval]
cond						
Capsule	**.8870318**	**.1995902**	**-0.53**	**0.594**	**.5707042**	**1.378692**
CT colonography	**1.139612**	**.2635305**	**0.57**	**0.572**	**.7243027**	**1.793056**
female						
Yes	**1.116424**	**.2651117**	**0.46**	**0.643**	**.7009684**	**1.778115**
cond#female						
Capsule#Yes	**.8280574**	**.2740884**	**-0.57**	**0.569**	**.4328239**	**1.584198**
CT colonography#Yes	**1.021262**	**.3416495**	**0.06**	**0.950**	**.5301261**	**1.967412**
cage						
65-69	**.6718341**	**.1127177**	**-2.37**	**0.018**	**.4835605**	**.9334119**
70-74	**.7439943**	**.1335862**	**-1.65**	**0.100**	**.5232798**	**1.057804**
ethnicity						
BAME	**1.742306**	**.9845806**	**0.98**	**0.326**	**.5755841**	**5.274**
idep						
1 marker	**1.33407**	**.2194894**	**1.75**	**0.080**	**.9663482**	**1.84172**
2-3 markers	**.9924304**	**.269328**	**-0.03**	**0.978**	**.583041**	**1.689278**
paid						
Yes	**1.015197**	**.1667701**	**0.09**	**0.927**	**.7357331**	**1.400813**
num						
Correct	**1.215624**	**.1644366**	**1.44**	**0.149**	**.9325193**	**1.584677**
_cons	**.6736522**	**.153843**	**-1.73**	**0.084**	**.4305717**	**1.053964**

Note: **_cons** estimates baseline odds.

```
  Thursday September 5 11:12:10 2019   Page 8

29 .
30 . //No time about BAME interaction//
31 . logit datt7 i.cond##i.ethnicity i.cage i.female i.idep i.paid i.num if comp==1, or

   note: 1.cond#1.ethnicity != 0 predicts failure perfectly
         1.cond#1.ethnicity dropped and 4 obs not used

   note: 3.cond#1.ethnicity omitted because of collinearity
   Iteration 0:   log likelihood = -139.95813
   Iteration 1:   log likelihood = -131.43249
   Iteration 2:   log likelihood =    -126.68
   Iteration 3:   log likelihood = -123.90107
   Iteration 4:   log likelihood =  -123.2927
   Iteration 5:   log likelihood = -123.26786
   Iteration 6:   log likelihood = -123.26785

   Logistic regression                             Number of obs     =        950
                                                   LR chi2(11)       =      33.38
                                                   Prob > chi2       =     0.0005
   Log likelihood = -123.26785                     Pseudo R2         =     0.1193
```

datt7	Odds Ratio	Std. Err.	z	P>|z|	[95% Conf.	Interval]
cond						
Capsule	.225937	.1167816	-2.88	0.004	.0820391	.6222342
CT colonography	.3317475	.1608767	-2.28	0.023	.1282414	.858197
ethnicity						
BAME	71.17544	79.73479	3.81	0.000	7.920736	639.5798
cond#ethnicity						
Colonoscopy#BAME	1	(empty)				
Capsule#BAME	.2360667	.3916317	-0.87	0.384	.0091391	6.097719
CT colonography#BAME	1	(omitted)				
cage						
65-69	.9844457	.4644365	-0.03	0.973	.3904959	2.481801
70-74	1.270358	.6398335	0.48	0.635	.4733764	3.409147
female						
Yes	.7283684	.280484	-0.82	0.410	.3424253	1.549303
idep						
1 marker	1.327865	.5964158	0.63	0.528	.5505965	3.202392
2-3 markers	2.433038	1.419385	1.52	0.127	.7754885	7.633479
paid						
Yes	3.022953	1.249684	2.68	0.007	1.344455	6.796991
num						
Correct	.6374225	.2473719	-1.16	0.246	.2979147	1.363839
_cons	.0482849	.0262249	-5.58	0.000	.0166533	.1399981

Note: **_cons** estimates baseline odds.

```
32 .
33 . //No time about employment interaction//
34 . logit datt7 i.cond##i.paid i.ethnicity i.cage i.female i.idep i.num if comp==1, or

   Iteration 0:   log likelihood =  -140.0949
   Iteration 1:   log likelihood = -129.40018
   Iteration 2:   log likelihood = -126.37467
   Iteration 3:   log likelihood =  -126.3263
   Iteration 4:   log likelihood = -126.32608
   Iteration 5:   log likelihood = -126.32608

   Logistic regression                             Number of obs     =        954
                                                   LR chi2(12)       =      27.54
                                                   Prob > chi2       =     0.0065
   Log likelihood = -126.32608                     Pseudo R2         =     0.0983
```

Thursday September 5 11:12:10 2019 Page 9

datt7	Odds Ratio	Std. Err.	z	P>|z|	[95% Conf.	Interval]
cond						
Capsule	.4443503	.2622262	-1.37	0.169	.1397664	1.412695
CT colonography	.5453833	.3208585	-1.03	0.303	.1721571	1.727741
paid						
Yes	3.409665	1.811371	2.31	0.021	1.203689	9.65849
cond#paid						
Capsule#Yes	.2084142	.2547593	-1.28	0.200	.0189865	2.287761
CT colonography#Yes	.6946165	.6397408	-0.40	0.692	.1142325	4.223773
ethnicity						
BAME	8.265565	6.210024	2.81	0.005	1.895632	36.04052
cage						
65-69	1.03754	.4843493	0.08	0.937	.4155685	2.5904
70-74	1.229042	.6137913	0.41	0.680	.4618182	3.270861
female						
Yes	.7356454	.2819938	-0.80	0.423	.3470394	1.559403
idep						
1 marker	1.407705	.6223427	0.77	0.439	.591831	3.34831
2-3 markers	2.431126	1.412986	1.53	0.126	.7781867	7.595062
num						
Correct	.5895268	.2287447	-1.36	0.173	.2755658	1.261194
_cons	.0408934	.023251	-5.62	0.000	.0134178	.1246311

Note: **_cons** estimates baseline odds.

```
35 .
36 . //Difficulty with transport and deprivation interaction//
37 . logit datt9 i.cond##i.idep i.cage i.female i.ethnicity i.paid i.num if comp==1, or

Iteration 0:   log likelihood = -376.10599
Iteration 1:   log likelihood = -361.04686
Iteration 2:   log likelihood = -358.60263
Iteration 3:   log likelihood = -358.59978
Iteration 4:   log likelihood = -358.59978

Logistic regression                             Number of obs     =        954
                                                LR chi2(14)       =      35.01
                                                Prob > chi2       =     0.0015
Log likelihood = -358.59978                     Pseudo R2         =     0.0465
```

datt9	Odds Ratio	Std. Err.	z	P>|z|	[95% Conf.	Interval]
cond						
Capsule	.6689234	.1965032	-1.37	0.171	.3761206	1.189668
CT colonography	.5796323	.1791188	-1.76	0.078	.3163101	1.062165
idep						
1 marker	1.551238	.5872859	1.16	0.246	.738619	3.257889
2-3 markers	2.259239	1.1653	1.58	0.114	.8220878	6.208779
cond#idep						
Capsule#1 marker	1.046429	.5651208	0.08	0.933	.3630955	3.015773
Capsule#2-3 markers	2.351092	1.660775	1.21	0.226	.5888354	9.387398
CT colonography#1 marker	1.206273	.6866612	0.33	0.742	.3952813	3.681165
CT colonography#2-3 markers	1.584024	1.237581	0.59	0.556	.3425522	7.324815
cage						
65-69	.7956792	.189795	-0.96	0.338	.498539	1.269922
70-74	.6638411	.1776491	-1.53	0.126	.3928945	1.121637
female						
Yes	1.310302	.259888	1.36	0.173	.8882644	1.93286

Thursday September 5 11:12:11 2019 Page 10

ethnicity						
BAME	3.29872	2.040796	1.93	0.054	.9811572	11.09053
paid						
Yes	.8836746	.2128248	-0.51	0.608	.5511736	1.41676
num						
Correct	.7531362	.1499798	-1.42	0.155	.5097591	1.11271
_cons	.2011304	.0606306	-5.32	0.000	.1113996	.363138

Note: **_cons** estimates baseline odds.

```
38 .
39 . //Health problems and gender interaction//
40 . logit datt10 i.cond##i.female i.cage i.ethnicity i.idep i.paid i.num if comp==1, or

Iteration 0:   log likelihood = -302.59055
Iteration 1:   log likelihood = -293.95422
Iteration 2:   log likelihood = -289.70446
Iteration 3:   log likelihood = -289.54772
Iteration 4:   log likelihood = -289.54738
Iteration 5:   log likelihood = -289.54738

Logistic regression                             Number of obs     =        954
                                                LR chi2(12)       =      26.09
                                                Prob > chi2       =     0.0104
Log likelihood = -289.54738                     Pseudo R2         =     0.0431
```

datt10	Odds Ratio	Std. Err.	z	P>|z|	[95% Conf.	Interval]
cond						
Capsule	.8579749	.2803729	-0.47	0.639	.4521839	1.627924
CT colonography	.6854997	.245683	-1.05	0.292	.3395759	1.383814
female						
Yes	.7217563	.2606936	-0.90	0.367	.355584	1.465004
cond#female						
Capsule#Yes	.5703324	.3195649	-1.00	0.316	.1901903	1.710282
CT colonography#Yes	.6423506	.3828119	-0.74	0.458	.1997535	2.065617
cage						
65-69	.9063112	.2517804	-0.35	0.723	.5257834	1.56224
70-74	.8010558	.2442216	-0.73	0.467	.4407101	1.456037
ethnicity						
BAME	6.379531	3.809814	3.10	0.002	1.97905	20.56462
idep						
1 marker	1.348598	.3652431	1.10	0.269	.7931407	2.293057
2-3 markers	2.626326	.9337083	2.72	0.007	1.308369	5.271895
paid						
Yes	.9490002	.2590525	-0.19	0.848	.5557895	1.6204
num						
Correct	.9549643	.2155266	-0.20	0.838	.6135909	1.486262
_cons	.1505126	.0531107	-5.37	0.000	.0753726	.3005608

Note: **_cons** estimates baseline odds.

```
  Thursday September 5 11:12:11 2019   Page 11

41 .
42 . //Health problems and BAME interaction//
43 . logit datt10 i.cond##i.ethnicity i.cage i.female i.idep i.paid i.num if comp==1, or

   Iteration 0:   log likelihood = -302.59055
   Iteration 1:   log likelihood = -294.25034
   Iteration 2:   log likelihood = -292.74312
   Iteration 3:   log likelihood = -290.23031
   Iteration 4:   log likelihood = -289.55165
   Iteration 5:   log likelihood = -289.50732
   Iteration 6:   log likelihood = -289.50731

   Logistic regression                             Number of obs     =        954
                                                   LR chi2(12)       =      26.17
                                                   Prob > chi2       =     0.0102
   Log likelihood = -289.50731                     Pseudo R2         =     0.0432
```

datt10	Odds Ratio	Std. Err.	z	P>|z|	[95% Conf.	Interval]
cond						
Capsule	.6875368	.1819878	-1.42	0.157	.4092482	1.155061
CT colonography	.5434485	.158273	-2.09	0.036	.3070831	.9617471
ethnicity						
BAME	2.668504	3.201338	0.82	0.413	.2541573	28.01774
cond#ethnicity						
Capsule#BAME	2.369605	3.631279	0.56	0.573	.1175603	47.76299
CT colonography#BAME	5.661841	9.042489	1.09	0.278	.2474625	129.5406
cage						
65-69	.9066002	.2523885	-0.35	0.725	.5253512	1.564523
70-74	.7970324	.2438844	-0.74	0.458	.4375385	1.451896
female						
Yes	.5380308	.1284885	-2.60	0.009	.3369222	.859181
idep						
1 marker	1.332997	.3621348	1.06	0.290	.782679	2.270256
2-3 markers	2.5314	.8957882	2.62	0.009	1.26516	5.06496
paid						
Yes	.993958	.2734556	-0.02	0.982	.5796786	1.704311
num						
Correct	.962673	.2180531	-0.17	0.867	.6175538	1.500662
_cons	.1727472	.0568114	-5.34	0.000	.0906727	.3291136

```
   Note: _cons estimates baseline odds.

44 .
45 . //Health problems and deprivation interaction//
46 . logit datt10 i.cond##i.dep i.ethnicity i.cage i.female i.paid i.num if comp==1, or

   note: 3.cond#3.depr != 0 predicts failure perfectly
         3.cond#3.depr dropped and 1 obs not used

   note: 1.cond#3.depr identifies no observations in the sample
   note: 2.cond#3.depr omitted because of collinearity
   Iteration 0:   log likelihood = -302.48909
   Iteration 1:   log likelihood = -294.18407
   Iteration 2:   log likelihood = -289.98635
   Iteration 3:   log likelihood = -289.83164
   Iteration 4:   log likelihood = -289.83128
   Iteration 5:   log likelihood = -289.83128

   Logistic regression                             Number of obs     =        953
                                                   LR chi2(15)       =      25.32
                                                   Prob > chi2       =     0.0459
   Log likelihood = -289.83128                     Pseudo R2         =     0.0418
```

Thursday September 5 11:12:11 2019   Page 12

datt10	Odds Ratio	Std. Err.	z	P>|z|	[95% Conf.	Interval]
cond						
Capsule	.7088625	.2289895	-1.07	0.287	.3763497	1.335157
CT colonography	.5338427	.1891195	-1.77	0.076	.2666036	1.068958
depr						
1	1.375126	.612255	0.72	0.474	.5745892	3.290999
2	2.160367	1.200865	1.39	0.166	.7267371	6.422109
3	2.412483	2.730994	0.78	0.437	.2623504	22.18435
cond#depr						
Colonoscopy#3	1	(empty)				
Capsule#1	.8794388	.5557166	-0.20	0.839	.2548779	3.034444
Capsule#2	1.193254	1.049022	0.20	0.841	.2130191	6.684163
Capsule#3	1	(omitted)				
CT colonography#1	1.116122	.7644453	0.16	0.873	.2915502	4.272773
CT colonography#2	1.624588	1.464046	0.54	0.590	.2777523	9.502306
CT colonography#3	1	(empty)				
ethnicity						
BAME	6.434172	3.838078	3.12	0.002	1.998658	20.71319
cage						
65-69	.936774	.2627153	-0.23	0.816	.5406523	1.623124
70-74	.8234025	.2533449	-0.63	0.528	.4505199	1.504909
female						
Yes	.53614	.128135	-2.61	0.009	.3356178	.8564686
paid						
Yes	.9559571	.2622917	-0.16	0.870	.5583276	1.63677
num						
Correct	.9552938	.2165329	-0.20	0.840	.61263	1.489621
_cons	.1700755	.0577478	-5.22	0.000	.0874225	.3308722

Note: **_cons** estimates baseline odds.

```
47 .
48 . log close
      name:  <unnamed>
       log:  S:\FPHS_BSH_CancerComm\QQ PROJECTS 2012-17\BOWEL SCREENING PROJECT\Alternatives to
  log type:  smcl
 closed on:   5 Sep 2019, 11:08:22
```
