# Supplementary material for: Preferences for different diagnostic modalities to follow up abnormal colorectal cancer screening results: a hypothetical vignette study
Source: BMJ Open. 2020 Jul 26;10(7):e035264. doi: 10.1136/bmjopen-2019-035264 (PMC7383951; doi:10.1136/bmjopen-2019-035264)
Supplement: Author's manuscript [file bmjopen-2019-035264.draft_revisions.pdf]

BMJ Open is committed to open peer review. As part of this commitment we make the peer review history of every article we publish publicly available.

When an article is published we post the peer reviewers' comments and the authors' responses online. We also post the versions of the paper that were used during peer review. These are the versions that the peer review comments apply to.

The versions of the paper that follow are the versions that were submitted during the peer review process. They are not the versions of record or the final published versions. They should not be cited or distributed as the published version of this manuscript.

BMJ Open is an open access journal and the full, final, typeset and author-corrected version of record of the manuscript is available on our site with no access controls, subscription charges or pay-per-view fees (http://bmjopen.bmj.com).

If you have any questions on BMJ Open's open peer review process please email info.bmjopen@bmj.com

BMJ Open

Preferences for different diagnostic modalities to follow up abnormal colorectal cancer screening results: a hypothetical vignette study

Journal:	*BMJ Open*
Manuscript ID	bmjopen-2019-035264
Article Type:	Original research
Date Submitted by the Author:	30-Oct-2019
Complete List of Authors:	Kaushal, Aradhna; University College London, Behavioural Science and Health<br>Stoffel , Sandro; University College London, Business School; University of Aberdeen<br>Kerrison, Robert; University College London, Epidemiology and Public Health<br>von Wagner, Christian; UCL, Department of Epidemiology and Public Health
Keywords:	PREVENTIVE MEDICINE, PUBLIC HEALTH, Endoscopy < GASTROENTEROLOGY

SCHOLARONE™
Manuscripts

I, the Submitting Author has the right to grant and does grant on behalf of all authors of the Work (as defined in the below author licence), an exclusive licence and/or a non-exclusive licence for contributions from authors who are: i) UK Crown employees; ii) where BMJ has agreed a CC-BY licence shall apply, and/or iii) in accordance with the terms applicable for US Federal Government officers or employees acting as part of their official duties; on a worldwide, perpetual, irrevocable, royalty-free basis to BMJ Publishing Group Ltd ("BMJ") its licensees and where the relevant Journal is co-owned by BMJ to the co-owners of the Journal, to publish the Work in this journal and any other BMJ products and to exploit all rights, as set out in our licence.

The Submitting Author accepts and understands that any supply made under these terms is made by BMJ to the Submitting Author unless you are acting as an employee on behalf of your employer or a postgraduate student of an affiliated institution which is paying any applicable article publishing charge ("APC") for Open Access articles. Where the Submitting Author wishes to make the Work available on an Open Access basis (and intends to pay the relevant APC), the terms of reuse of such Open Access shall be governed by a Creative Commons licence – details of these licences and which Creative Commons licence will apply to this Work are set out in our licence referred to above.

Other than as permitted in any relevant BMJ Author's Self Archiving Policies, I confirm this Work has not been accepted for publication elsewhere, is not being considered for publication elsewhere and does not duplicate material already published. I confirm all authors consent to publication of this Work and authorise the granting of this licence.

For peer review only

Preferences for different diagnostic modalities to follow up abnormal colorectal cancer screening results: a hypothetical vignette study

Aradhna Kaushal[1], Sandro T. Stoffel[1,2], Robert Kerrison[1], and Christian von Wagner[1]

[1] Research Department of Behavioural Science and Health, University College London

[2] Business School, University of Aberdeen

Corresponding author:

Christian von Wagner, Research Department of Behavioural Science and Health, University College London, 1-19 Torrington Place, London, WC1E 7HB

Email: c.wagner@ucl.ac.uk

Word Count: 3160/3000

Tables and Figures: 5/5

10/24/2019

For peer review only - http://bmjopen.bmj.com/site/about/guidelines.xhtml

Abstract

Objectives

In England, a significant proportion of people who take part in the national bowel cancer screening programme (BCSP) and have a positive faecal occult blood test (FOBt) result, do not attend follow-up colonoscopy (CC). The aim of this study was to investigate differences in intended participation in a follow-up investigation by diagnostic modality offered including (CC), computed tomography colonography (CTC), or capsule endoscopy (CE).

Setting

We performed a randomised online experiment with individuals who had previously completed a FOBt as part of the English BCSP.

Methods

Participants (n=953) were randomly allocated to receive one of three online vignettes asking participants to imagine they had received an abnormal FOBt result, and that they had been invited for a follow-up test. The follow-up test offered was either: CC (n=346), CTC (n=302), or CE (n=305). Participants were then asked how likely they were to have their allocated test or if they refused either of the other tests. Respondents were also asked to cite possible emotional and practical barriers to follow up testing. Multi-variable logistic regression models were used to investigate intentions.

Results

Intention to have the test was higher in the CTC group (96.7%) compared with the CC group (91.8%; Odds Ratio [OR]: 2.64; 95% Confidence Intervals [CI]: 1.22-5.73). CTC was considered less “off-putting” (OR: 0.66, 95% CI: 0.46-0.94) and less uncomfortable

For peer review only - http://bmjopen.bmj.com/site/about/guidelines.xhtml

compared to CC (OR: 0.51, 95%; CI: 0.34-0.77). For those who did not intend the test they were offered, CE (39.7%) or no investigation (34.5%) was preferable to CC (8.6%) or CTC (17.2%).

Conclusions

Alternative tests have the potential to increase attendance at diagnostic follow-up appointments.

Word count: 267/250

For peer review only

For peer review only - http://bmjopen.bmj.com/site/about/guidelines.xhtml

Introduction

Colorectal cancer (CRC) is the 4th most common cancer in the United Kingdom (UK), accounting for 12% of all cancer diagnosis, and is the second leading cause of cancer death. [1, 2] Screening can reduce mortality and improve survival by detecting CRC at an earlier stage, when treatment is more likely to be successful. [3] In England, the national bowel cancer screening programme (BCSP) sends a screening test to all men and women aged 60 to 74 to be completed at home. The test is offered once every two years, and can detect small amounts of blood in the stool, which may be indicative of CRC. This test until recently was the 'faecal occult blood test' (FOBt) and was replaced with the more sensitive 'feacal immunochemical test' (FIT) in June 2019.

Around 2% of people who complete a FOBt have a positive test result and are invited for further investigation, usually CC. Despite being at increased risk, approximately 14% of those with an abnormal test result fail to complete their diagnostic follow up. [4-7] There are a number of reasons why a CC in a small proportion of these patients would not be appropriate, such as clinical decisions based on frailty, or having had a recent CC outside the BSCP. [8, 9]

Previous research point towards a range of psychological and practical factors which may influence decisions to have colorectal cancer screening. [9, 11, 12] Plumb et al (2017) reviewed the medical records of patients who 170 patients to identify patient factors associated with non-attendance at CC after a positive FOBt and found the most cited reason was "unwillingness to complete the test". [9] For some patients, this was due to anxiety about pain, and risks but in the majority of cases, the reason was unspecified. This is supported by a recent systematic review on incomplete diagnostic follow-up after an abnormal CRC screening result which identified perception of pain as the most commonly cited reason in addition to 'embarrassment' and 'being too busy'. [13] Consequently, for a vast majority of this

For peer review only - http://bmjopen.bmj.com/site/about/guidelines.xhtml

group of patients there is a strong need to develop patient or system level interventions to remove modifiable barriers to investigating the cause of their FOB test result. [10]

Some of these barriers may be directly or indirectly related to the nature of CC (CC). CC involves inserting a long flexible tube with a tiny camera on the end into the anus in order to examine the bowel. If polyps (small, potentially cancerous growths) are found, they are removed during the procedure. The test requires the patient to restrict their diet, take a strong laxative, and arrange for someone to take them home afterwards due to drowsiness caused by a sedative. After the procedure, most people will need to rest for the remainder of the day. [14]

Considering the practical and emotional barriers which may inhibit someone from accepting the offer of further investigation via CC, it has been suggested that offering alternative tests, such as CTC or capsule endoscopy, may be more acceptable to patients. [15-17] These tests differ in terms of preparation, time in hospital and level of perceived invasiveness. Both of these tests, as with CC, require the patient to restrict their diet and take a laxative. In CTC, a small tube is used to inflate the bowel after which x-rays are used to take images of the bowel after which most people are able to resume their daily activities. [18] In capsule endoscopy, the patient ingests a capsule containing a small camera which takes photos inside the bowel and transmits them wirelessly to a receiver worn by the patient. [19] Patients are able to carry on with their normal activity and the capsule is passed through the body after eight hours. For both of these tests, a CC is usually recommended to remove any polyps if they are found.

CTC and CE could be offered individuals several follow-up tests to choose from, in the hope that providing individuals with a choice increases their feeling of autonomy and intrinsic motivation. [20-22] While offering choice seems promising, there exists no evidence that offering more than one screening test influences adherence and patient satisfaction. [23]

For peer review only - http://bmjopen.bmj.com/site/about/guidelines.xhtml

The present study aimed to evaluate whether offering different screening tests, such as CTC, or CE, increases intentions to attend for further investigation, when compared to offering a CC.

Methods

Design

We performed a randomised online experiment with a survey company called: 'Dynata' (formerly Survey Sampling International and Research Now). Potentially eligible men and women were invited to participate in the online experiment by Dynata, who invited them from their panel if they were: 1) aged 60-74 years and 2) lived in England. Potentially eligible men and women were informed that they would be rewarded with 'Dynata points', which they could exchange for money, or a donation to a charity, if they complete the survey.

Individuals who agreed to participate in the survey were asked about their screening history, which was used to filter ineligible adults. Only individuals who had completed a FOBt previously, and received a normal result, were eligible to participate in the survey. We excluded participants who had previously had a CC, a previous diagnosis of colorectal cancer or had parts of their bowel removed. Once eligibility to participate had been established using these criteria, participants were then randomly allocated to one of three experimental conditions in which they were asked to read a hypothetical vignette (see Supplementary Appendix 1).

Depending on the condition individuals were allocated to, the vignette asked them to imagine that their next FOBt result was abnormal, and they were being invited for a follow-up test consisting of either CC, a CTC, or a CE. Each vignette included a some images of the test, a short description about what happens before, during and after the test, and any risks

For peer review only - http://bmjopen.bmj.com/site/about/guidelines.xhtml

associated with the offered test. After reading the information, participants were required to successfully answer a 'comprehension check question' about the test (what happens during the test, how long it takes, and what happens afterwards), to ensure they had understood the information. If answered incorrectly, the information about the test was presented again and the participant is provided with another opportunity to answer the question. Once the participant answered the comprehension check question correctly, they were asked about their intention to have the follow-up test. Details of the comprehension check questions can be found in the Supplementary Appendix 1.

Measures

Intention to have follow-up test: On answering the comprehension check question correctly, participants were asked to indicate their intention to book an appointment (*"Considering all the information presented above, would you take up the offer of this test?"*), using a four-point Likert scale, with response options: '*definitely not*', '*probably not*', '*probably, yes*' and '*definitely, yes*'. [24, 25] These responses were dichotomised ('*probably, yes*' or '*definitely, yes*' vs. '*probably not*' or '*definitely not*') due to the relative lack of 'probably not' and 'definitely not' responses (see Supplementary Appendix 2).

Barriers for follow-up test: After indicating their intentions, participants were then asked to indicate how they felt about potential barriers for not wanting the tests ("*Please read each statement and select how strongly you agree or disagree with it?*"), using a four-point Likert scale, with response options: *'strongly agree', 'slightly agree', 'slightly disagree'* and *'strongly disagree'*. The barriers featured six emotional and four practical items, and were derived from previous surveys. [9, 26] The six emotional barrier items were: "*The preparation for the test (restricted diet and strong laxative) puts me off", "The test looks like it would be uncomfortable", "I would be embarrassed about taking the test", "I would worry about the*

For peer review only - http://bmjopen.bmj.com/site/about/guidelines.xhtml

risks associated with the test", "I would be afraid of getting an abnormal result" and "Doing the test would make me worry more about bowel cancer". The four practical barrier items were: *"I would not have time to do the test", "I have other problems to worry about", "It would be difficult to arrange transport to the hospital"* and *"I have other health problems that are more important"*. These responses were dichotomised ('*strongly agree'* or *'slightly agree'* vs '*slightly disagree'* or *'strongly disagree'*) due to the relative lack of 'strongly agree' responses (see Supplementary Appendix 2).

Preference of disinclined study participants. Study participants who indicated that they would not (probably or definitely) have the offered follow-up test were presented with information about the other two tests and asked which of the three tests they would prefer ("*Which of the two tests would you prefer to have*?"), with response options *"colonoscopy", "capsule", "CT colonography"* and *"none of them"*.

Numeracy skills. Numeracy skills were assessed by the question: 'Which of the following numbers represents the biggest risk of getting a disease?', with answer options '1/10', '1/100', '1/1000' and 'I don't know'. This measure was adapted from Lipkus and colleagues, who previously validated the question as a measure for numeracy skills. [15]

Socio-demographic variables. Details of the participant's age, gender, ethnicity, employment status, education, car and home ownership were collected at the end of the survey. Ethnicity data were recoded as 'White' (White British or Other White Background) and "BAME" (Black, Asian and minority ethnic) due to the small numbers in each group. The three variables on education, car and home ownership were used to calculate a proxy measure for socio-economic deprivation. One point was given to a participant if their household did not own a car or van, if they had no formal qualifications and if they did not

For peer review only - http://bmjopen.bmj.com/site/about/guidelines.xhtml

own their own home. [27-29] Scores, therefore, ranged from 0 to 3, with high scores indicating higher levels of deprivation.

Sample size calculation

Sample size of this study was calculated prior to data collection based on the results of a soft launch. We calculated that we needed approximately 300 participants per condition to detect differences of at least 5 percentage points in proportion of intenders effect size between conditions, with a power of 80% and an alpha value of 0.05. [30]

Statistical analysis

Our primary outcome was intention to have the offered follow-up test after exposure to the allocated vignette. Our secondary outcomes were the responses to the perceived emotional and practical barrier questions. Descriptive statistics were used to report the socio-demographic characteristics of the study population. Differences between the socio-demographic characteristics of trial arms were compared using the Chi-Square test of independence. Uni-variable and multi-variable logistic regression models were used to investigate the effect of offering alternative follow-up tests on participants' intentions to have further investigations, as well as their perceived emotional and practical barriers of the test offered. Covariates that were included in the regression models were age, gender, ethnicity, deprivation score, employment status and numeracy. All statistical analysis was conducted using Stata/SE (version 15.1). [31] The survey, data and Stata codes for the experiment are available via Open Science Framework:

https://osf.io/fx69t/?view_only=46ba2185c26940a89c19bde28da40a9c.

For peer review only - http://bmjopen.bmj.com/site/about/guidelines.xhtml

Results

Participants

In total, 1,926 adults responded to the online invitation. Of these, 481 (25.0%) were excluded due to their age or medical history (i.e. previous bowel cancer diagnosis or removal of part of the bowel). A further 372 (19.3%) were excluded as they had never been invited for, or completed, a FOBt, and an additional 46 (2.4%) were excluded as they had previously received an abnormal FOBt result. Of the remaining 1,027 adults who were eligible for inclusion, 953 (92.8%) completed the survey (see Figure 1 for an overview of participants through the study).

- Figure 1 here -

Around half of participants were male (54.5%), of a White ethnic background (98.6%), not in paid employment (75.1%), had a formal education (62.7%), owned car (87.1%), and owned a house (87.4%).

- Table 1 here -

Intention to have the allocated test

Table 1 shows that there a large majority (93.7%) would intend to have the allocated test, although this was graded by ethnicity (61.5% vs 94.4%) and deprivation (2-3 markers: 87.9% vs. 0 markers: 95.5%). The proportion of intenders was highest among those who were offered CTC, followed by those who were offered capsule, and finally those who were offered CC (96.7% vs. 93.4% vs. 91.8% respectively, $\chi2$=(2, N=953) =6.64, p=0.036). In the multi-variable model, participants remained more likely to accept further investigation if the offered test was CTC compared to CC, after adjusting for co-variates (Table 2; OR: 2.64, 95% CI: 1.22–5.73, p=0.014). Offering CE did not significantly increase intentions compared

For peer review only - http://bmjopen.bmj.com/site/about/guidelines.xhtml

to CC (OR: 1.28, 95% CI: 0.69–2.35, p=0.432). The multi-variable analysis also revealed that intention to undergo follow-up tests was lower among BAME groups than White ethnic groups (OR: 0.09, 95% CI: 0.03–0.29, p<0.001), as well as more deprived individuals (1 marker: OR: 0.45, 95% CI: 0.25–0.84, p=0.012; 2-3 markers: OR: 0.34, 95% CI: 0.15–0.79, p=0.012).

Preferences of disinclined study participants

In total, 58 (6.1%) study participants did not intend to have their offered follow-up test. Independently from the initial offer, most disinclined study participants would either choose CE (N=23, 39.7%) or none of the tests (N=20, 34.5%). A Fisher's exact test demonstrated that preferences were not influenced by the initial random allocation (p=0.806).

- Table 2 here -

Perceived emotional barriers

Those who were randomised to CTC were less likely to agree that the test is off-putting (OR: 0.66, 95% CI: 0.46–0.94, p=0.022) or uncomfortable (OR: 0.51, 95% CI: 0.34–0.77, p=0.001) than those who were offered a CC. Participants who were offered CE as a follow-up test were less likely to perceive the test as uncomfortable (OR: 0.11: 95% CI: 0.07–0.16, p<0.001), embarrassing (OR: 0.33, 95% CI: 0. 23–0.48, p<0.001), or creating worries about the risks associated with the test (OR: 0.70, 95% CI: 0.51–0.97, p=0.031) than those who were offered a CC (Table 3).

The table also shows that women were more likely to perceive the offered tests as off-putting (OR: 1.41, 95% CI: 1.06–1.88, p=0.018), embarrassing (OR: 1.83, 95% CI: 1.36–2.47, p<0.001) and causing worries about the risks (OR: 1.46, 95% CI: 1.11–1.91, p=0.007) and results of the test (OR: 1.57, 95% CI: 1.20–2.05, p=0.001).

For peer review only - http://bmjopen.bmj.com/site/about/guidelines.xhtml

Higher deprivation was associated with worrying about the risks associated with the test (OR: 1.50, 95% CI: 1.08-2.08, p=0.014) and numeracy was positively associated with being afraid of the results (OR: 1.32, 95% CI: 1.01-1.72, p=0.39). Sub-group analysis did not reveal any significant interactions between these demographic variables and test allocation (Supplementary Appendix 3).

- Table 3 here -

Perceived practical barriers

Study participants who were offered CE, instead of CC, were less likely to think that they wouldn't have the time to do the test (OR: 0.27, 95% CI: 0.10–0.70, p=0.007). There were no other differences in practical barriers by test offered (See table 4). Health problems were cited as a barrier by participants with a higher number of deprivation markers (OR: 2.54, 95% CI: 1.27-5.07 , p=0.009), those with a BAME background (OR: 6.45, 95% CI:2.01-20.73 , p=0.002) and by men (OR: 1.85, 95% CI: 2.94-1.16, p=0.009). Those from BAME groups (OR:9.37, 95% CI:2.23-39.36, p=0.002), and those who were in paid in employment (OR:2.42, 95% CI:1.09-5.38 , p=0.03) were more likely to report not having enough time as a barrier to a follow-up investigation. Participants with a higher number of deprivation markers were also more likely to cite difficulties with transport (OR: 3.52 , 95% CI:1.96-6.31, p<0.001) as a barrier to having a follow-up investigation. Sub-group analysis did not reveal any significant interactions between these demographic variables and test allocation (Supplementary Appendix 3) .

- Table 4 here -

For peer review only - http://bmjopen.bmj.com/site/about/guidelines.xhtml

Discussion

This study investigated whether offering alternative follow-up tests increases intention to undergo further investigation in the context of CRC screening. The results of our study show that, while the vast majority of individuals would accept any test, offering CTC instead of CC may increase acceptance of follow-up tests. Findings from our study suggest that this is due to individuals perceiving CTC as less 'off-putting' and 'uncomfortable' than CC. No differences were found in intention between those randomised to CE compared to CC despite CE appearing less uncomfortable, less embarrassing, causing less worry about the risks associated with the test, and taking test time. However, for the small number of participants who did not intend to take the offered test, CE appeared to be the preferred test when presented with all options.

The findings from this study are supported by a trial in France by Pioche et al. (2018) which randomised patients who had an abnormal FOBt result, but who refused CC, to be invited for either CE or CTC. [32] They found that CTC was more appealing than CE (7.4% vs. 5.0% having the investigation) but no differences were found in clinical outcomes. The authors concluded that those who refuse CC are difficult to recruit into the screening programme and that simply offering a different test is not effective, however the study is limited by the small sample size.

Our study also observed a lower likelihood of ethnic minorities to accept any follow-up test. This is supported by a recent systematic review of characteristics of incomplete diagnostic follow-up after a positive bowel screening test identified non-White participants as less likely to attend in 9 out of 10 studies. [13] Analysis of barriers suggest that other health problems and not having enough time may be additional contributing factors to intention. Previous research

For peer review only - http://bmjopen.bmj.com/site/about/guidelines.xhtml

has identified embarrassment as a potential barrier to colorectal cancer screening, although this was not found in this study. [33]

We also found that not having enough time, and difficulties arranging transport are barriers to screening for people in employment, those with a higher level of deprivation and those from a BAME background. This is consistent with evidence from an American study, which showed that the location of the CC appointment and the availability of evening and weekend appointments are important factors limiting uptake. [34]

While our study provides support that individuals perceive follow-up tests differently, we only offered one test at random. Future research should analyse whether offering individuals the choice between different tests increases intentions. [23] This could also include investigation of other diagnostic tests such as stool DNA tests. [35]

Strengths and limitations

Our study has several limitations. Firstly, our study used hypothetical scenarios and non-representative online study samples. Furthermore, the experiments lacked behavioural validation, in that they only measured intentions and choice in a hypothetical setting. The next step would be to test the effect of offering alternative follow-up tests under more ecologically valid conditions in a randomised controlled trial. A strength of our study is that we used comprehension checks to ensure that all study participants sufficiently engaged with the provided information about the follow-up test.

Conclusions

While the results from this randomised online experiment show a high acceptance of the current practice of the NHS Bowel Screening Programme to invite individuals who have an abnormal

For peer review only - http://bmjopen.bmj.com/site/about/guidelines.xhtml

FOBt result for a follow-up CC, our study also provides some evidence that offering CTC, instead of CC, may increase the total number of participants undertaking a follow-up test.

For peer review only

For peer review only - http://bmjopen.bmj.com/site/about/guidelines.xhtml

Abbreviations

CRC: colorectal cancer; FOBt: faecal occult blood test; CC: Colonoscopy; CTC: CT Colonography; CE: Capsule Endoscopy; OR: Odds Ratio; 95% CI: 95% Confidence Interval

Declarations

Ethics and consent to participate

This research project was approved by UCL Research ethics committee (approval number 14687/001). All participants were asked to give explicit written consent for their data to be used and published as part of this research project before taking part in this study.

Consent for publication

Not applicable.

Competing interests

The authors have declared that no competing interests exist.

Supporting information

Supporting file contains demographic characteristics of the study samples, additional description of results and all outcome measures of the experimental studies.

Patient and Public Involvement

The design of this study followed a direct question posed by representatives of Public Health England and was informed by previous research on this topic and close collaboration with experts involved in administering relevant tests (see acknowledgments).

Availability of data and materials

All data files and materials are publicly available via the Open Science Framework and can be accessed at https://osf.io/fx69t/?view_only=46ba2185c26940a89c19bde28da40a9c.

For peer review only - http://bmjopen.bmj.com/site/about/guidelines.xhtml

Funding

This report presents independent research commissioned and funded by the National Institute for Health Research (NIHR) Policy Research Programme, conducted through the Policy Research Unit in Cancer Awareness, Screening and Early Diagnosis, PR-PRU-1217-21601. The views expressed are those of the authors and not necessarily those of the NIHR, the Department of Health and Social Care or its arm's length bodies, or other Government Departments.

Authors' contributions

AK, STS and CW developed the study concept. All authors contributed to the study design. AK, STS and CW performed the data analysis and interpretation. AK and STS drafted the manuscript, RK and CW provided critical revisions. All authors approved the final version of the manuscript for submission.

Acknowledgements

We would like to acknowledge the contribution to the study materials by Professor Owen Epstein, Tim Rayne and Dr. Andrew Plumb.

For peer review only

For peer review only - http://bmjopen.bmj.com/site/about/guidelines.xhtml

References

1. Cancer Research UK. Bowel cancer incidence statistics, https://www.cancerresearchuk.org/health-professional/cancer-statistics/statistics-by-cancer-type/bowel-cancer/incidence (2018, accessed 24th October 2018).

2. Cancer Research UK. Bowel cancer mortality statistics, https://www.cancerresearchuk.org/health-professional/cancer-statistics/statistics-by-cancer-type/bowel-cancer/mortality#ref- (2018, accessed 29th Aug 2019).

3. Logan RF, Patnick J, Nickerson C, et al. Outcomes of the Bowel Cancer Screening Programme (BCSP) in England after the first 1 million tests. *Gut* 2012; 61: 1439-1446.

4. Hirst Y, Stoffel S, Baio G, et al. Uptake of the English Bowel (Colorectal) Cancer Screening Programme: an update 5 years after the full roll-out. *European Journal of Cancer* 2018; 103: 267-273.

5. Kearns B, Whyte S, Seaman HE, et al. Factors associated with completion of bowel cancer screening and the potential effects of simplifying the screening test algorithm. 2016; 114: 327.

6. Beshara A, Ahoroni M, Comanester D, et al. Association Between Time to Colonoscopy After a Positive Guaiac Fecal Test Result and Risk of Colorectal Cancer and Advanced Stage Disease at Diagnosis. *International journal of cancer* 2019.

7. Kaalby L, Rasmussen M, Zimmermann-Nielsen E, et al. Time to colonoscopy, cancer probability, and precursor lesions in the Danish colorectal cancer screening program. *Clinical Epidemiology* 2019; 11: 659.

8. Paynter S. A pilot study exploring primary care involvement in re-engaging patients with the Bowel Cancer Screening Programme who have tested positive on screening but failed to complete colonoscopy. Poster, Public Health England,, 2015.

9. Plumb AA, Ghanouni A, Rainbow S, et al. Patient factors associated with non-attendance at colonoscopy after a positive screening faecal occult blood test. *Journal of medical screening* 2017; 24: 12-19.

10. Selby K, Baumgartner C, Levin TR, et al. Interventions to improve follow-up of positive results on fecal blood tests: a systematic review. *Annals of internal medicine* 2017; 167: 565-575.

11. Honein-AbouHaidar GN, Kastner M, Vuong V, et al. Systematic review and meta-study synthesis of qualitative studies evaluating facilitators and barriers to participation in colorectal cancer screening. *Cancer Epidemiol Biomarkers Prev*. 2016; 25: 907-917.

12. Power E, Miles A, Von Wagner C, et al. Uptake of colorectal cancer screening: system, provider and individual factors and strategies to improve participation. 2009; 5: 1371-1388.

For peer review only - http://bmjopen.bmj.com/site/about/guidelines.xhtml

13. Dalton AJJoPH. Incomplete diagnostic follow-up after a positive colorectal cancer screening test: a systematic review. *J Public Health (Oxf)*. 2017; 40: e46-e58.

14. Carlson CM, Kirby KA, Casadei MA, et al. Lack of follow-up after fecal occult blood testing in older adults: inappropriate screening or failure to follow up? *Archives of internal medicine* 2011; 171: 249-256.

15. Groth S, Krause H, Behrendt R, et al. Capsule colonoscopy increases uptake of colorectal cancer screening. *BMC gastroenterology* 2012; 12: 80.

16. Rex DK and Lieberman DA. A survey of potential adherence to capsule colonoscopy in patients who have accepted or declined conventional colonoscopy. *Journal of clinical gastroenterology* 2012; 46: 691-695.

17. Pooler BD, Baumel MJ, Cash BD, et al. Screening CT colonography: multicenter survey of patient experience, preference, and potential impact on adherence. *American journal of roentgenology* 2012; 198: 1361-1366.

18. Kim DH, Pickhardt PJ, Taylor AJ, et al. CT colonography versus colonoscopy for the detection of advanced neoplasia. *New England journal of medicine* 2007; 357: 1403-1412.

19. Iddan G, Meron G, Glukhovsky A, et al. Wireless capsule endoscopy. *Nature* 2000; 405: 417.

20. Katz I and Assor A. When choice motivates and when it does not. *Educational Psychology Review* 2007; 19: 429.

21. Patall EA, Cooper H and Robinson JC. The effects of choice on intrinsic motivation and related outcomes: a meta-analysis of research findings. *Psychological bulletin* 2008; 134: 270.

22. Zuckerman M, Porac J, Lathin D, et al. On the importance of self-determination for intrinsically-motivated behavior. *Personality and social psychology bulletin* 1978; 4: 443-446.

23. Partin MR, Powell AA, Burgess DJ, et al. Bringing an organizational perspective to the optimal number of colorectal cancer screening options debate. *Journal of general internal medicine* 2012; 27: 376-380.

24. Stoffel ST, Goodwin M, Sieverding M, et al. Testing verbal quantifiers for social norms messages in cancer screening: evidence from an online experiment. *BMC public health* 2019; 19: 658.

25. Stoffel ST, Hirst Y, Ghanouni A, et al. Testing active choice for screening practitioner's gender in endoscopy among disinclined women: An online experiment. *Journal of medical screening* 2019; 26: 98-103.

26. Smith S, McGregor L, Raine R, et al. Inequalities in cancer screening participation: examining differences in perceived benefits and barriers. *Psycho-Oncology* 2016; 25: 1168-1174.

For peer review only - http://bmjopen.bmj.com/site/about/guidelines.xhtml

27. McGregor LM, von Wagner C, Vart G, et al. The impact of supplementary narrative-based information on colorectal cancer screening beliefs and intention. *BMC cancer* 2015; 15: 162.

28. Von Wagner C, Bonello B, Stoffel S, et al. Barriers to bowel scope (flexible sigmoidoscopy) screening: a comparison of non-responders, active decliners and non-attenders. *BMC public health* 2018; 18: 1161.

29. Wardle J, McCaffery K, Nadel M, et al. Socioeconomic differences in cancer screening participation: comparing cognitive and psychosocial explanations. *Social science & medicine* 2004; 59: 249-261.

30. Cohen J. *Statistical power analysis for the behavioral sciences*. Routledge, 2013.

31. StataCorp. Stata Statistical Software: Release 16. College Station, TX: StataCorp LLC, 2019.

32. Pioche M, Ganne C, Gincul R, et al. Colon capsule versus computed tomography colonography for colorectal cancer screening in patients with positive fecal occult blood test who refuse colonoscopy: a randomized trial. *Endoscopy* 2018; 50: 761-769.

33. Robb KA, Solarin I, Power E, et al. Attitudes to colorectal cancer screening among ethnic minority groups in the UK. *BMC public health* 2008; 8: 34.

34. O'Malley A, Beaton E, Yabroff K, et al. Patient and provider barriers to colorectal cancer screening in the primary care safety-net. 2004; 39: 56-63.

35. Imperiale TF, Ransohoff DF, Itzkowitz SH, et al. Multitarget stool DNA testing for colorectal-cancer screening. *New England Journal of Medicine* 2014; 370: 1287-1297.

For peer review only

For peer review only - http://bmjopen.bmj.com/site/about/guidelines.xhtml

Tables and Figures

Figure 1. Participant flow diagram

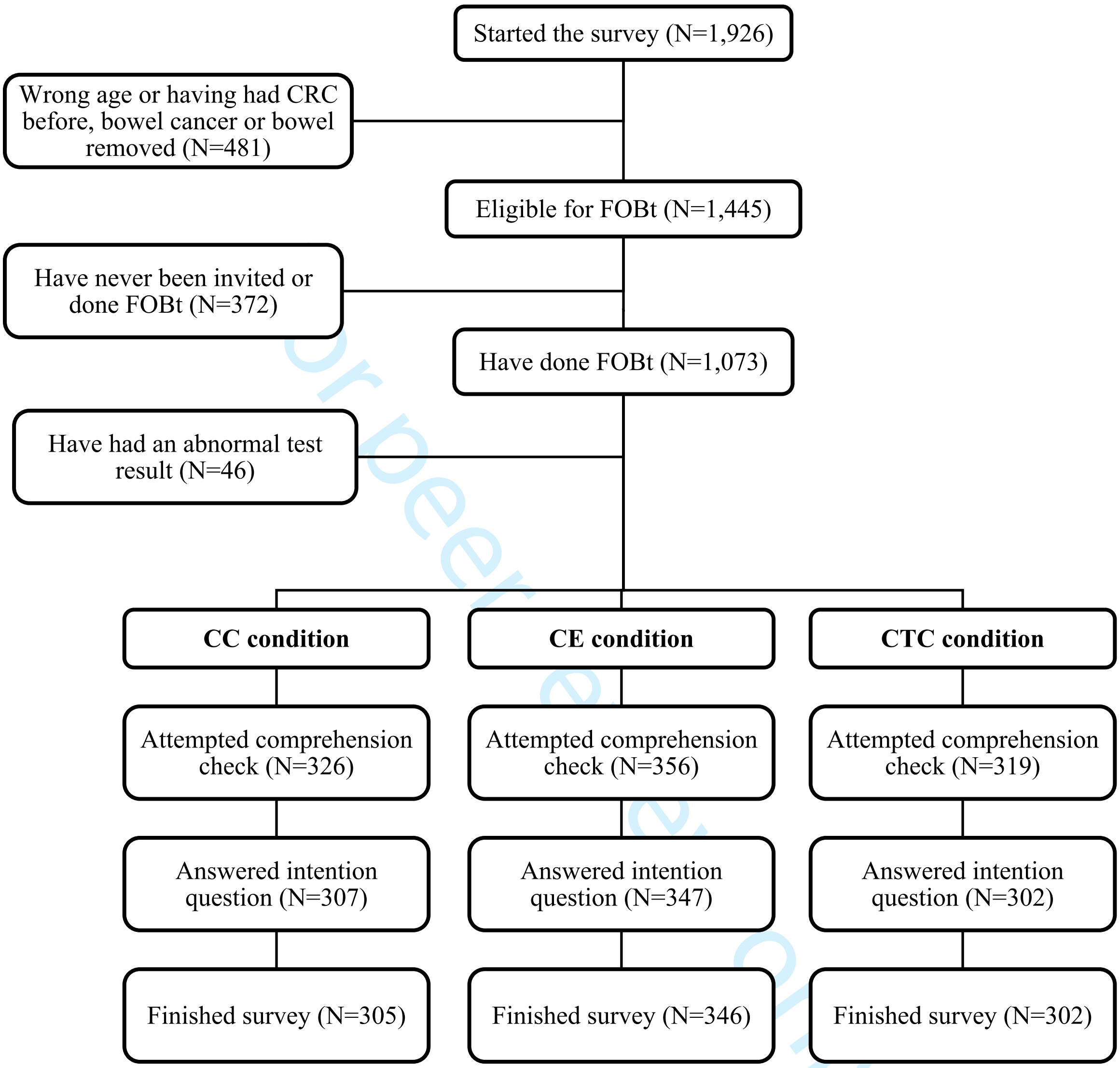

For peer review only - http://bmjopen.bmj.com/site/about/guidelines.xhtml

Table 1. Socio-demographic characteristics of the study population (N=953)

	Colonoscopy condition (N=305)		Capsule endoscopy condition (N=346)		CT Colonography condition (N=302)		Overall (N=953)		p-value*
**Age**									
60-64	98	(32.1%)	94	(27.2%)	109	(36.1%)	301	(31.6%)	
65-69	123	(40.3%)	125	(36.1%)	109	(36.1%)	357	(37.5%)	0.028
70-74	84	(27.5%)	127	(36.7%)	84	(27.8%)	295	(31.0%)	
**Gender**									
Male	151	(49.5%)	201	(58.1%)	167	(55.3%)	519	(54.5%)	0.085
Female	154	(50.5%)	145	(41.9%)	135	(44.7%)	434	(45.5%)	
**Ethnicity**									
White	301	(98.7%)	341	(98.6%)	298	(98.7%)	940	(98.6%)	0.987
BMAE	4	(1.3%)	5	(1.4%)	4	(1.3%)	13	(1.4%)	
**Paid employment**									
No	221	(72.5%)	260	(75.1%)	235	(77.8%)	716	(75.1%)	0.312
Yes	84	(27.5%)	86	(24.9%)	67	(22.2%)	237	(24.9%)	
**Education**									
No A levels	119	(39.0%)	125	(36.1%)	112	(37.4%)	356	(37.4%)	0.744
A levels or higher	186	(61.0%)	221	(63.9%)	190	(62.6%)	597	(62.6%)	
**Car ownership**									
No	36	(11.8%)	46	(13.3%)	41	(13.6%)	123	(12.9%)	0.780
Yes	269	(88.2%)	300	(86.7%)	261	(86.4%)	830	(87.1%)	
**House ownership**									
No	44	(14.4%)	46	(13.3%)	30	(9.9%)	120	(12.6%)	0.220
Yes	261	(85.6%)	300	(86.7%)	272	(90.1%)	833	(87.4%)	
**Individual social deprivation**									
0 markers	223	(73.1%)	242	(69.9%)	221	(73.2%)	686	(72.0%)	
1 marker	58	(19.0%)	80	(23.1%)	63	(20.8%)	201	(21.1%)	0.659
2-3 markers	24	(7.9%)	24	(6.9%)	18	(5.9%)	66	(6.9%)	
**Numeracy question**									
Wrong	163	(53.4%)	180	(52.0%)	151	(50.0%)	494	(51.8%)	0.695
Correct	142	(46.6%)	166	(48.0%)	151	(50.0%)	459	(48.2%)	

*p-value refers to Chi-Square test of independence if not stated differently

The three demographic questions on education, car and house ownership were used to calculate a proxy measure for socio-economic deprivation. One point was given to an individual if their household did not have a car or van, if they had no formal qualifications and if they did not own their own home [1, 2]. Scores, therefore, ranged from 0 to 3, with high scores indicating higher levels of social deprivation. Due to low frequencies, we collapsed the two groups that either had a score of 2 or 3 into one group called 2-3 markers.

For peer review only - http://bmjopen.bmj.com/site/about/guidelines.xhtml

Table 2. Logistic regression models on wanting to have the test probably or definitely (N=953)

	Unadjusted model		Adjusted model	
	OR	95% CI	OR	95% CI
**Condition**				
Colonoscopy	Ref.		Ref.	
Capsule endoscopy	1.254	0.696 - 2.259	1.276	0.694 - 2.347
CT colonography	2.607	**1.230 - 5.527***	2.642	**1.218 - 5.733***
**Age**				
60-64			Ref.	
65-69			0.702	0.360 - 1.371
70-74			1.424	0.629 - 3.224
**Gender**				
Male			Ref.	
Female			1.090	0.623 - 1.908
**Ethnicity**				
White			Ref.	
BAME			0.086	**0.026 - 0.286****
**Deprivation**				
0 makers			Ref.	
1 marker			0.454	**0.246 - 0.839***
2-3 markers			0.344	**0.149 - 0.794***
**Paid employment**				
No			Ref.	
Yes			0.868	0.453 - 1.665
**Numeracy task**				
Wrong			Ref.	
Correct			1.576	0.892 - 2.783

OR: Odds ratio; CI: Confidence intervals; BAME: Black, Asian and Minority Ethnic; * p<0.05; ** p<0.01

For peer review only

For peer review only - http://bmjopen.bmj.com/site/about/guidelines.xhtml

Table 3. Multi-variable logistic regression on emotional barrier items (N=953).

	Off-putting		Uncomfortable		Embarrassing		Worry about risks of test		Afraid of results		Worry about cancer	
	OR	95% CI	OR	95% CI	OR	95% CI	OR	95% CI	OR	95% CI	OR	95% CI
**Condition**												
Colonoscopy	Ref.		Ref.		Ref.		Ref.		Ref.		Ref.	
Capsule endoscopy	0.747	0.533 - 1.046	0.106	**0.072 - 0.156****	0.330	**0.227 - 0.480****	0.699	**0.506 - 0.967***	0.750	0.544 - 1.034	0.816	0.591 - 1.126
CT colonography	0.661	**0.464 - 0.942***	0.510	**0.338 - 0.768****	0.723	0.512 - 1.020	0.728	0.522 - 1.017	0.915	0.656 - 1.277	1.146	0.827 - 1.589
**Age**												
60-64	Ref.		Ref.		Ref.		Ref.		Ref.		Ref.	
65-69	0.989	0.694 - 1.410	0.905	0.622 - 1.318	0.805	0.560 - 1.158	0.852	0.610 - 1.190	0.675	**0.483 - 0.942***	0.673	**0.484 - 0.934***
70-74	1.049	0.717 - 1.536	0.894	0.599 - 1.334	0.881	0.594 - 1.307	0.884	0.617 - 1.266	0.706	0.494 - 1.010	0.742	0.522 - 1.055
**Gender**												
Male	Ref.		Ref.		Ref.		Ref.		Ref.		Ref.	
Female	1.414	**1.062 - 1.883***	1.027	0.759 - 1.389	1.832	**1.357 - 2.472****	1.456	**1.109 - 1.910****	1.567	**1.197 - 2.051****	1.054	0.806 - 1.378
**Ethnicity**												
White	Ref.		Ref.		Ref.		Ref.		Ref.		Ref.	
BAME	2.224	0.732 - 6.753	1.221	0.328 - 4.553	1.189	0.338 - 4.185	2.022	0.663 - 6.166	3.967	0.859 - 18.327	1.764	0.583 - 5.341
**Deprivation**												
0 markers	Ref.		Ref.		Ref.		Ref.		Ref.		Ref.	
1 marker	1.058	0.747 - 1.500	1.357	0.934 - 1.971	0.993	0.687 - 1.434	1.501	**1.085 - 2.077***	1.356	0.971 - 1.893	1.336	0.968 - 1.845
2-3 markers	1.432	0.840 - 2.440	0.646	0.364 - 1.147	1.285	0.731 - 2.261	1.264	0.749 - 2.136	0.680	0.406 - 1.139	0.988	0.581 - 1.679
**Paid employment**												
No	Ref.		Ref.		Ref.		Ref.		Ref.		Ref.	
Yes	0.986	0.696 - 1.396	1.453	0.998 - 2.114	1.417	0.998 - 2.014	1.105	0.797 - 1.532	1.191	0.858 - 1.652	1.012	0.734 - 1.396
**Numeracy task**												
Wrong	Ref.		Ref.		Ref.		Ref.		Ref.		Ref.	
Correct	1.034	0.778 - 1.374	0.884	0.656 - 1.191	1.130	0.839 - 1.520	1.083	0.827 - 1.418	1.322	**1.014 - 1.724***	1.221	0.937 - 1.591

OR: Odds ratio; CI: Confidence intervals; BAME: Black, Asian and Minority Ethnic; * *p*<0.05; ** *p*<0.01

For peer review only

For peer review only - http://bmjopen.bmj.com/site/about/guidelines.xhtml

Table 4. Multi-variable logistic regression on practical barrier items (n=953).

	No time		Other problems		Difficulties with transport		Health problems	
	OR	95% CI	OR	95% CI	OR	95% CI	OR	95% CI
**Condition**								
Colonoscopy	Ref.		Ref.		Ref.		Ref.	
Capsule endoscopy	0.268	**0.103 - .698****	0.753	0.519 - 1.093	0.756	0.482 - 1.184	0.702	0.421 - 1.172
CT colonography	0.458	0.192 - 1.090	0.803	0.549 - 1.176	0.642	0.397 - 1.037	0.577	0.331 - 1.008
**Age**								
60-64	Ref.		Ref.		Ref.		Ref.	
65-69	1.058	0.426 - 2.628	0.914	0.620 - 1.347	0.774	0.487 - 1.231	0.919	0.533 - 1.583
70-74	1.279	0.483 - 3.386	1.025	0.678 - 1.549	0.653	0.388 - 1.101	0.803	0.442 - 1.460
**Gender**								
Male	Ref.		Ref.		Ref.		Ref.	
Female	0.733	0.347 - 1.549	1.179	0.862 - 1.612	1.334	0.906 - 1.964	0.539	**0.338 - 0.860****
**Ethnicity**								
White	Ref.		Ref.		Ref.		Ref.	
BAME	9.369	**2.230 - 39.355****	2.282	0.734 - 7.099	3.319	0.989 - 11.133	6.452	**2.008 - 20.729****
**Deprivation**								
0 markers	Ref.		Ref.		Ref.		Ref.	
1 marker	1.399	0.590 - 3.317	1.106	0.758 - 1.614	1.650	**1.055 - 2.578***	1.349	0.793 - 2.292
2-3 markers	2.410	0.775 - 7.493	1.372	0.772 - 2.440	3.517	**1.960 - 6.313****	2.535	**1.268 - 5.070****
**Paid employment**								
No	Ref.		Ref.		Ref.		Ref.	
Yes	2.421	**1.091 - 5.375***	0.967	0.661 - 1.415	0.879	0.549 - 1.407	0.952	0.558 - 1.626
**Numeracy task**								
Wrong	Ref.		Ref.		Ref.		Ref.	
Correct	0.589	0.276 - 1.255	0.831	0.609 - 1.135	0.741	0.503 - 1.093	0.955	0.614 - 1.485

OR: Odds ratio; CI: Confidence intervals; BAME: Black, Asian and Minority Ethnic; * $p<0.05$; ** $p<0.01$

For peer review only

For peer review only - http://bmjopen.bmj.com/site/about/guidelines.xhtml

Table 1. Intention to have the test for colonoscopy, CT colonography, and capsule endoscopy n (%).

N=953	Colonoscopy	Capsule endoscopy	CT colonography
Definitely not	4 (1.3)	6 (1.7)	2 (0.7)
Probably not	21 (6.9)	17 (4.9)	8 (2.7)
Yes, probably	114 (37.4)	123 (35.6)	115 (38.1)
Yes, definitely	166 (54.4)	200 (57.8)	177 (58.6)

Table 2.Emotional barriers to each test n(%)

N=953	Colonoscopy	Capsule endoscopy	CT colonography
**Off-putting**			
Strongly disagree	106 (34.8)	134 (38.7)	122 (40.4)
Slightly disagree	94 (30.8)	116 (33.5)	103 (34.1)
Slightly agree	83 (27.2)	84 (24.3)	67 (22.2)
Strongly agree	22 (7.2)	12 (3.5)	10 (3.3)
**Uncomfortable**			
Strongly disagree	14 (4.6)	87 (25.1)	27 (8.9)
Slightly disagree	32 (10.5)	127 (36.7)	51 (16.9)
Slightly agree	183 (60.0)	119 (34.4)	188 (62.3)
Strongly agree	76 (24.9)	13 (3.8)	36 (11.9)
**Embarrassing**			
Strongly disagree	115 (37.7)	201 (58.1)	135 (44.7)
Slightly disagree	75 (24.6)	89 (25.7)	77 (25.5)
Slightly agree	92 (30.2)	51 (14.7)	76 (25.2)
Strongly agree	23 (7.5)	5 (1.5)	14 (4.6)
**Worry about risks**			
Strongly disagree	82 (26.9)	110 (31.8)	103 (34.1)
Slightly disagree	95 (31.2)	121 (35.0)	95 (31.5)
Slightly agree	111 (36.4)	107 (30.9)	92 (30.5)
Strongly agree	17 (5.6)	8 (2.3)	12 (4.0)
**Afraid of results**			
Strongly disagree	64 (21.0)	87 (25.1)	68 (22.5)
Slightly disagree	54 (17.7)	73 (21.1)	55 (18.2)
Slightly agree	140 (45.9)	154 (44.5)	146 (48.3)
Strongly agree	47 (15.4)	32 (9.3)	33 (10.9)
**Worry about cancer**			
Strongly disagree	87 (28.5)	126 (36.4)	86 (28.5)
Slightly disagree	97 (31.8)	99 (28.6)	85 (28.2)
Slightly agree	94 (30.8)	108 (31.2)	112 (37.1)
Strongly agree	27 (8.9)	13 (3.8)	19 (6.3)

For peer review only

Table 3.Practical barriers to each test n(%)

N=953	Colonoscopy	Capsule endoscopy	CT colonography
**No time**			
Strongly disagree	226 (74.1)	285 (82.4)	246 (81.5)
Slightly disagree	61 (20.0)	55 (15.9)	48 (15.9)
Slightly agree	14 (4.6)	6 (1.7)	6 (2.0)
Strongly agree	4 (1.3)	0 (0.0)	2 (0.7)
**Other problems**			
Strongly disagree	140 (45.9)	182 (52.6)	144 (47.7)
Slightly disagree	88 (28.9)	94 (27.2)	94 (31.1)
Slightly agree	61 (20.0)	61 (17.6)	49 (16.2)
Strongly agree	16 (5.3)	9 (2.6)	15 (5.0)
**Difficulties with transport**			
Strongly disagree	188 (61.6)	239 (69.1)	210 (69.5)
Slightly disagree	67 (22.0)	63 (18.2)	58 (19.2)
Slightly agree	39 (12.8)	39 (11.3)	27 (8.9)
Strongly agree	11 (3.6)	5 (1.5)	7 (2.3)
**Health problems**			
Strongly disagree	183 (60.0)	218 (63.0)	209 (68.9)
Slightly disagree	85 (27.9)	96 (27.8)	71 (23.5)
Slightly agree	27 (8.9)	26 (7.5)	14 (4.6)
Strongly agree	10 (3.3)	6 (1.7)	9 (3.0)

For peer review only

```
  Thursday September 5 11:12:06 2019   Page 1

                                                    ___  ____  ____  ____  ____(R)
                                                   /__    /   ____/   /   ____/
                                                  ___/   /   /___/   /   /___/
                                                    Statistics/Data Analysis

      name:  <unnamed>
       log:  S:\FPHS_BSH_CancerComm\QQ PROJECTS 2012-17\BOWEL SCREENING PROJECT\Alternatives to
  log type:  smcl
 opened on:   5 Sep 2019, 11:08:18

1 . use "S:\FPHS_BSH_CancerComm\QQ PROJECTS 2012-17\BOWEL SCREENING PROJECT\Alternatives to Colon

2 .
3 . //Intention and BAME interaction//
4 . logit int2 i.cond##i.ethnicity i.cage i.female i.idep i.paid i.num if comp==1, or

note: 1.cond#1.ethnicity != 0 predicts success perfectly
      1.cond#1.ethnicity dropped and 4 obs not used

note: 3.cond#1.ethnicity omitted because of collinearity
Iteration 0:   log likelihood = -218.29837
Iteration 1:   log likelihood = -215.64434
Iteration 2:   log likelihood = -206.51414
Iteration 3:   log likelihood = -199.21258
Iteration 4:   log likelihood = -197.68211
Iteration 5:   log likelihood = -197.37916
Iteration 6:   log likelihood = -197.37691
Iteration 7:   log likelihood = -197.37691

Logistic regression                             Number of obs     =        949
                                                LR chi2(11)       =      41.84
                                                Prob > chi2       =     0.0000
Log likelihood = -197.37691                     Pseudo R2         =     0.0958

------------------------------------------------------------------------------------
               int2 | Odds Ratio   Std. Err.      z    P>|z|     [95% Conf. Interval]
--------------------+---------------------------------------------------------------
               cond |
            Capsule |   1.440134   .4558544     1.15   0.249     .7744031    2.678175
    CT colonography |   3.164895   1.327974     2.75   0.006     1.390591    7.203097
                    |
          ethnicity |
               BAME |   .0257632   .0289846    -3.25   0.001     .0028403    .2336873
                    |
     cond#ethnicity |
   Colonoscopy#BAME |          1  (empty)
       Capsule#BAME |   1.525905   2.263561     0.28   0.776     .0833368    27.93948
CT colonography#BAME |         1  (omitted)
                    |
               cage |
              65-69 |   .7060353   .2438926    -1.01   0.314     .3587465    1.389521
              70-74 |   1.402867   .5943763     0.80   0.424     .6114676    3.218544
                    |
             female |
                Yes |    1.06484   .3075379     0.22   0.828     .6045717    1.875517
                    |
               idep |
           1 marker |   .4684608    .148785    -2.39   0.017     .2513786    .8730079
        2-3 markers |   .3456216   .1480694    -2.48   0.013     .1492566    .8003283
                    |
               paid |
                Yes |   .7472369   .2478844    -0.88   0.380     .3900185    1.431632
                    |
                num |
            Correct |   1.487324   .4351085     1.36   0.175     .8382839    2.638882
              _cons |   14.26229   6.135926     6.18   0.000     6.137408    33.14312
------------------------------------------------------------------------------------
Note: _cons estimates baseline odds.
```

For peer review only

```
  Thursday September 5 11:12:08 2019   Page 2

 5 .
 6 . //Intention and deprivation interaction//
 7 . logit int2 i.cond##i.idep  i.cage i.female i.ethnicity i.paid i.num if comp==1, or

Iteration 0:   log likelihood = -218.55008
Iteration 1:   log likelihood = -203.06951
Iteration 2:   log likelihood = -199.19849
Iteration 3:   log likelihood = -199.10343
Iteration 4:   log likelihood = -199.10307
Iteration 5:   log likelihood = -199.10307

Logistic regression                             Number of obs     =        953
                                                LR chi2(14)       =      38.89
                                                Prob > chi2       =     0.0004
Log likelihood = -199.10307                     Pseudo R2         =     0.0890
```

int2	Odds Ratio	Std. Err.	z	P>|z|	[95% Conf.	Interval]
cond						
Capsule	1.740958	.7370709	1.31	0.190	.7593019	3.99174
CT colonography	3.305797	1.748944	2.26	0.024	1.172046	9.324114
idep						
1 marker	.5428541	.2653282	-1.25	0.211	.208279	1.414884
2-3 markers	.8346543	.6618163	-0.23	0.820	.1764289	3.948604
cond#idep						
Capsule#1 marker	.7549797	.5310209	-0.40	0.689	.1902111	2.99664
Capsule#2-3 markers	.1872543	.1874193	-1.67	0.094	.0263318	1.33163
CT colonography#1 marker	.6383686	.5497961	-0.52	0.602	.1180246	3.452792
CT colonography#2-3 markers	.4718537	.6540561	-0.54	0.588	.0311833	7.139919
cage						
65-69	.6643002	.2293366	-1.18	0.236	.3376788	1.306848
70-74	1.385225	.5810532	0.78	0.437	.6087953	3.151879
female						
Yes	1.135806	.3270864	0.44	0.658	.6459178	1.997243
ethnicity						
BAME	.0868887	.0536264	-3.96	0.000	.0259188	.2912809
paid						
Yes	.8567131	.2863368	-0.46	0.644	.4449803	1.649415
num						
Correct	1.533339	.44838	1.46	0.144	.8644292	2.719864
_cons	12.76679	5.550371	5.86	0.000	5.44529	29.93245

Note: **_cons** estimates baseline odds.

```
 8 .
 9 . //Off-putting and gender interaction//
10 . logit datt1 i.cond##i.female i.cage i.ethnicity i.idep i.paid i.num if comp==1, or

Iteration 0:   log likelihood = -575.64795
Iteration 1:   log likelihood = -567.00146
Iteration 2:   log likelihood = -566.95168
Iteration 3:   log likelihood = -566.95168

Logistic regression                             Number of obs     =        954
                                                LR chi2(12)       =      17.39
                                                Prob > chi2       =     0.1354
Log likelihood = -566.95168                     Pseudo R2         =     0.0151
```

For peer review only

Thursday September 5 11:12:08 2019 Page 3

datt1	Odds Ratio	Std. Err.	z	P>|z|	[95% Conf.	Interval]
cond						
Capsule	.5994014	.1453158	-2.11	0.035	.3726978	.9640035
CT colonography	.602084	.1528489	-2.00	0.046	.3660713	.9902584
female						
Yes	1.147654	.2795688	0.57	0.572	.7119644	1.849967
cond#female						
Capsule#Yes	1.561034	.5367613	1.30	0.195	.7956584	3.062653
CT colonography#Yes	1.193689	.4305356	0.49	0.624	.5886849	2.42047
cage						
65-69	.9965692	.1806268	-0.02	0.985	.6985996	1.42163
70-74	1.047513	.2039252	0.24	0.812	.7152387	1.534149
ethnicity						
BAME	2.271933	1.290318	1.44	0.148	.7463898	6.91553
idep						
1 marker	1.061183	.1891052	0.33	0.739	.7483492	1.504792
2-3 markers	1.403437	.3834878	1.24	0.215	.8214909	2.397634
paid						
Yes	.9865023	.1755985	-0.08	0.939	.6959585	1.39834
num						
Correct	1.039599	.1512154	0.27	0.789	.7817247	1.38254
_cons	.4533808	.1097497	-3.27	0.001	.2821064	.7286403

Note: **_cons** estimates baseline odds.

```
11 .
12 . //Embarrasing and gender interaction//
13 . logit datt3 i.cond##i.female i.cage i.ethnicity i.idep i.paid i.num if comp==1, or

Iteration 0:   log likelihood = -559.79953
Iteration 1:   log likelihood = -526.78936
Iteration 2:   log likelihood = -526.08874
Iteration 3:   log likelihood = -526.08791
Iteration 4:   log likelihood = -526.08791

Logistic regression                             Number of obs     =        954
                                                LR chi2(12)       =      67.42
                                                Prob > chi2       =     0.0000
Log likelihood = -526.08791                     Pseudo R2         =     0.0602
```

datt3	Odds Ratio	Std. Err.	z	P>|z|	[95% Conf.	Interval]
cond						
Capsule	.393843	.1049377	-3.50	0.000	.2336277	.6639295
CT colonography	.6117832	.158551	-1.90	0.058	.3681276	1.016709
female						
Yes	1.802571	.4375261	2.43	0.015	1.120175	2.900674
cond#female						
Capsule#Yes	.6950697	.2652284	-0.95	0.340	.32902	1.468366
CT colonography#Yes	1.382337	.4910231	0.91	0.362	.6890595	2.773136
cage						
65-69	.8078209	.1503881	-1.15	0.252	.5608556	1.163534
70-74	.889448	.1795823	-0.58	0.562	.5987705	1.321237
ethnicity						
BAME	1.155396	.7401264	0.23	0.822	.3292044	4.055045
idep						
1 marker	.9886713	.1860287	-0.06	0.952	.6837378	1.429599

For peer review only

```
  Thursday September 5 11:12:08 2019   Page 4

         2-3 markers |   1.281844   .3718801     0.86   0.392     .7259228    2.263498
                     |
                paid |
                 Yes |   1.439032   .2589492     2.02   0.043     1.011346    2.047583
                     |
                 num |
             Correct |   1.112985   .1693064     0.70   0.482     .8260481    1.499593
               _cons |    .420774   .1040708    -3.50   0.000     .2591312    .6832476
------------------------------------------------------------------------------------
Note: _cons estimates baseline odds.

14 .
15 . //Worry about test risk and gender interaction//
16 . logit datt4 i.cond##i.female i.cage i.ethnicity i.idep i.paid i.num if comp==1, or

Iteration 0:   log likelihood = -625.38047
Iteration 1:   log likelihood = -610.79631
Iteration 2:   log likelihood = -610.75012
Iteration 3:   log likelihood = -610.75012

Logistic regression                             Number of obs     =        954
                                                LR chi2(12)       =      29.26
                                                Prob > chi2       =     0.0036
Log likelihood = -610.75012                     Pseudo R2         =     0.0234

------------------------------------------------------------------------------------
               datt4 | Odds Ratio   Std. Err.      z    P>|z|     [95% Conf. Interval]
---------------------+--------------------------------------------------------------
                cond |
             Capsule |   .8625152   .1965071    -0.65   0.516     .5518706     1.34802
    CT colonography  |   .6402238   .1565886    -1.82   0.068      .396407    1.034004
                     |
              female |
                 Yes |   1.574237    .372587     1.92   0.055     .9899417    2.503402
                     |
         cond#female |
         Capsule#Yes |    .626443   .2078761    -1.41   0.159     .3269057     1.20044
CT colonography#Yes  |   1.312932   .4499751     0.79   0.427     .6706774    2.570221
                     |
                cage |
               65-69 |   .8532505   .1461122    -0.93   0.354     .6099795    1.193542
               70-74 |   .8931162   .1645699    -0.61   0.540     .6223884    1.281606
                     |
           ethnicity |
                BAME |    1.94679    1.10931     1.17   0.242     .6372233    5.947667
                     |
                idep |
            1 marker |   1.494679   .2483308     2.42   0.016     1.079261    2.069995
         2-3 markers |   1.271042   .3415635     0.89   0.372     .7506177     2.15229
                     |
                paid |
                 Yes |   1.121261   .1875352     0.68   0.494     .8078669    1.556228
                     |
                 num |
             Correct |   1.065082   .1471823     0.46   0.648     .8123747    1.396398
               _cons |    .532697   .1239152    -2.71   0.007     .3376564    .8403991
------------------------------------------------------------------------------------
Note: _cons estimates baseline odds.

17 .
```

```
  Thursday September 5 11:12:09 2019   Page 5

18 . //Worry about test risk and deprivation interaction//
19 . logit datt4 i.cond##i.idep i.cage i.female i.ethnicity i.paid i.num if comp==1, or

   Iteration 0:   log likelihood = -625.38047
   Iteration 1:   log likelihood = -609.00743
   Iteration 2:   log likelihood = -608.97148
   Iteration 3:   log likelihood = -608.97148

   Logistic regression                             Number of obs     =        954
                                                   LR chi2(14)       =      32.82
                                                   Prob > chi2       =     0.0031
   Log likelihood = -608.97148                     Pseudo R2         =     0.0262
```

datt4	Odds Ratio	Std. Err.	z	P>|z|	[95% Conf.	Interval]
cond						
Capsule	.8545529	.1688906	-0.80	0.426	.5801105	1.25883
CT colonography	.8738928	.1757055	-0.67	0.503	.5892709	1.295989
idep						
1 marker	2.773085	.8485567	3.33	0.001	1.522291	5.051596
2-3 markers	1.35632	.5985531	0.69	0.490	.5711117	3.221092
cond#idep						
Capsule#1 marker	.3593903	.1484572	-2.48	0.013	.159938	.8075719
Capsule#2-3 markers	1.287068	.7999347	0.41	0.685	.3806846	4.351488
CT colonography#1 marker	.4862063	.2070739	-1.69	0.090	.2110067	1.120327
CT colonography#2-3 markers	.5373902	.3794284	-0.88	0.379	.1346767	2.144307
cage						
65-69	.8475212	.1459977	-0.96	0.337	.6046707	1.187906
70-74	.8790054	.1626208	-0.70	0.486	.6116663	1.263189
female						
Yes	1.454996	.2032422	2.68	0.007	1.106525	1.91321
ethnicity						
BAME	2.084474	1.191957	1.28	0.199	.6796058	6.39346
paid						
Yes	1.107485	.1857581	0.61	0.543	.7971979	1.538543
num						
Correct	1.074747	.1491767	0.52	0.604	.8187623	1.410764
_cons	.4918955	.1081869	-3.23	0.001	.3196396	.7569813

```
   Note: _cons estimates baseline odds.

20 .
21 . //Afraid of results risk and age group interaction//
22 . logit datt5 i.cond##i.cage i.female i.ethnicity i.idep i.paid i.num if comp==1, or

   Iteration 0:   log likelihood = -649.10164
   Iteration 1:   log likelihood =   -626.968
   Iteration 2:   log likelihood = -626.80181
   Iteration 3:   log likelihood = -626.80112
   Iteration 4:   log likelihood = -626.80112

   Logistic regression                             Number of obs     =        954
                                                   LR chi2(14)       =      44.60
                                                   Prob > chi2       =     0.0000
   Log likelihood = -626.80112                     Pseudo R2         =     0.0344
```

For peer review only

Thursday September 5 11:12:09 2019 Page 6

datt5	Odds Ratio	Std. Err.	z	P>|z|	[95% Conf.	Interval]
cond						
Capsule	.9487913	.2971592	-0.17	0.867	.5135425	1.752932
CT colonography	.8040911	.2395326	-0.73	0.464	.4484756	1.441689
cage						
65-69	.6737809	.1962447	-1.36	0.175	.3807139	1.192446
70-74	.7619691	.2441478	-0.85	0.396	.4066287	1.427831
cond#cage						
Capsule#65-69	.8464529	.3448479	-0.41	0.682	.3809082	1.880985
Capsule#70-74	.618933	.2636364	-1.13	0.260	.2685788	1.426315
CT colonography#65-69	1.124773	.4530285	0.29	0.770	.5107682	2.476887
CT colonography#70-74	1.360613	.5960542	0.70	0.482	.5765568	3.210902
female						
Yes	1.579452	.217962	3.31	0.001	1.205152	2.070003
ethnicity						
BAME	3.903997	3.066424	1.73	0.083	.8373884	18.20087
idep						
1 marker	1.369841	.2341368	1.84	0.066	.9798976	1.91496
2-3 markers	.6881451	.1825012	-1.41	0.159	.4091994	1.157244
paid						
Yes	1.1609	.1946665	0.89	0.374	.835719	1.61261
num						
Correct	1.318004	.1794388	2.03	0.043	1.009323	1.721087
_cons	1.307052	.3353875	1.04	0.297	.7904531	2.161274

Note: **_cons** estimates baseline odds.

```
23 .
24 . //Afraid of results risk and gender interaction//
25 . logit datt5 i.cond##i.female i.cage i.ethnicity i.idep i.paid i.num if comp==1, or

Iteration 0:   log likelihood = -649.10164
Iteration 1:   log likelihood = -628.77429
Iteration 2:   log likelihood = -628.62076
Iteration 3:   log likelihood = -628.62007
Iteration 4:   log likelihood = -628.62007

Logistic regression                             Number of obs     =        954
                                                LR chi2(12)       =      40.96
                                                Prob > chi2       =     0.0000
Log likelihood = -628.62007                     Pseudo R2         =     0.0316
```

datt5	Odds Ratio	Std. Err.	z	P>|z|	[95% Conf.	Interval]
cond						
Capsule	.7984076	.1756849	-1.02	0.306	.5187078	1.228928
CT colonography	.9635103	.2206762	-0.16	0.871	.6150389	1.50942
female						
Yes	1.706078	.413164	2.21	0.027	1.061358	2.742432
cond#female						
Capsule#Yes	.8715473	.286892	-0.42	0.676	.4571896	1.661444
CT colonography#Yes	.8956024	.3066038	-0.32	0.747	.457838	1.751938
cage						
65-69	.6722562	.1145439	-2.33	0.020	.4813943	.9387906
70-74	.7060271	.1287507	-1.91	0.056	.4938519	1.00936
ethnicity						
BAME	3.943815	3.079102	1.76	0.079	.8537837	18.21735

For peer review only

Thursday September 5 11:12:10 2019 Page 7

idep						
1 marker	**1.355455**	**.2308021**	**1.79**	**0.074**	**.9708356**	**1.892452**
2-3 markers	**.6852833**	**.1808773**	**-1.43**	**0.152**	**.408507**	**1.149584**
paid						
Yes	**1.188009**	**.1987038**	**1.03**	**0.303**	**.8559524**	**1.648883**
num						
Correct	**1.321628**	**.1794319**	**2.05**	**0.040**	**1.01285**	**1.72454**
_cons	**1.28226**	**.2930399**	**1.09**	**0.277**	**.8193087**	**2.006802**

Note: **_cons** estimates baseline odds.

```
26 .
27 . //Worry about cancer and gender interaction//
28 . logit datt6 i.cond##i.female i.cage i.ethnicity i.idep i.paid i.num if comp==1, or

Iteration 0:   log likelihood = -638.84517
Iteration 1:   log likelihood = -629.17662
Iteration 2:   log likelihood = -629.16658
Iteration 3:   log likelihood = -629.16658

Logistic regression                             Number of obs     =        954
                                                LR chi2(12)       =      19.36
                                                Prob > chi2       =     0.0803
Log likelihood = -629.16658                     Pseudo R2         =     0.0152
```

datt6	Odds Ratio	Std. Err.	z	P>|z|	[95% Conf.	Interval]
cond						
Capsule	**.8870318**	**.1995902**	**-0.53**	**0.594**	**.5707042**	**1.378692**
CT colonography	**1.139612**	**.2635305**	**0.57**	**0.572**	**.7243027**	**1.793056**
female						
Yes	**1.116424**	**.2651117**	**0.46**	**0.643**	**.7009684**	**1.778115**
cond#female						
Capsule#Yes	**.8280574**	**.2740884**	**-0.57**	**0.569**	**.4328239**	**1.584198**
CT colonography#Yes	**1.021262**	**.3416495**	**0.06**	**0.950**	**.5301261**	**1.967412**
cage						
65-69	**.6718341**	**.1127177**	**-2.37**	**0.018**	**.4835605**	**.9334119**
70-74	**.7439943**	**.1335862**	**-1.65**	**0.100**	**.5232798**	**1.057804**
ethnicity						
BAME	**1.742306**	**.9845806**	**0.98**	**0.326**	**.5755841**	**5.274**
idep						
1 marker	**1.33407**	**.2194894**	**1.75**	**0.080**	**.9663482**	**1.84172**
2-3 markers	**.9924304**	**.269328**	**-0.03**	**0.978**	**.583041**	**1.689278**
paid						
Yes	**1.015197**	**.1667701**	**0.09**	**0.927**	**.7357331**	**1.400813**
num						
Correct	**1.215624**	**.1644366**	**1.44**	**0.149**	**.9325193**	**1.584677**
_cons	**.6736522**	**.153843**	**-1.73**	**0.084**	**.4305717**	**1.053964**

Note: **_cons** estimates baseline odds.

```
  Thursday September 5 11:12:10 2019   Page 8

29 .
30 . //No time about BAME interaction//
31 . logit datt7 i.cond##i.ethnicity i.cage i.female i.idep i.paid i.num if comp==1, or

   note: 1.cond#1.ethnicity != 0 predicts failure perfectly
         1.cond#1.ethnicity dropped and 4 obs not used

   note: 3.cond#1.ethnicity omitted because of collinearity
   Iteration 0:   log likelihood = -139.95813
   Iteration 1:   log likelihood = -131.43249
   Iteration 2:   log likelihood =    -126.68
   Iteration 3:   log likelihood = -123.90107
   Iteration 4:   log likelihood =  -123.2927
   Iteration 5:   log likelihood = -123.26786
   Iteration 6:   log likelihood = -123.26785

   Logistic regression                             Number of obs     =        950
                                                   LR chi2(11)       =      33.38
                                                   Prob > chi2       =     0.0005
   Log likelihood = -123.26785                     Pseudo R2         =     0.1193

   ------------------------------------------------------------------------------------
                 datt7 | Odds Ratio   Std. Err.      z    P>|z|     [95% Conf. Interval]
   --------------------+---------------------------------------------------------------
                  cond |
               Capsule |    .225937   .1167816    -2.88   0.004     .0820391    .6222342
      CT colonography  |   .3317475   .1608767    -2.28   0.023     .1282414     .858197
                       |
             ethnicity |
                  BAME |   71.17544   79.73479     3.81   0.000     7.920736    639.5798
                       |
        cond#ethnicity |
      Colonoscopy#BAME |          1  (empty)
          Capsule#BAME |   .2360667   .3916317    -0.87   0.384     .0091391    6.097719
  CT colonography#BAME |          1  (omitted)
                       |
                  cage |
                 65-69 |   .9844457   .4644365    -0.03   0.973     .3904959    2.481801
                 70-74 |   1.270358   .6398335     0.48   0.635     .4733764    3.409147
                       |
                female |
                   Yes |   .7283684    .280484    -0.82   0.410     .3424253    1.549303
                       |
                  idep |
              1 marker |   1.327865   .5964158     0.63   0.528     .5505965    3.202392
          2-3 markers  |   2.433038   1.419385     1.52   0.127     .7754885    7.633479
                       |
                  paid |
                   Yes |   3.022953   1.249684     2.68   0.007     1.344455    6.796991
                       |
                   num |
               Correct |   .6374225   .2473719    -1.16   0.246     .2979147    1.363839
                 _cons |   .0482849   .0262249    -5.58   0.000     .0166533    .1399981
   ------------------------------------------------------------------------------------
   Note: _cons estimates baseline odds.

32 .
33 . //No time about employment interaction//
34 . logit datt7 i.cond##i.paid i.ethnicity i.cage i.female i.idep i.num if comp==1, or

   Iteration 0:   log likelihood =  -140.0949
   Iteration 1:   log likelihood = -129.40018
   Iteration 2:   log likelihood = -126.37467
   Iteration 3:   log likelihood =  -126.3263
   Iteration 4:   log likelihood = -126.32608
   Iteration 5:   log likelihood = -126.32608

   Logistic regression                             Number of obs     =        954
                                                   LR chi2(12)       =      27.54
                                                   Prob > chi2       =     0.0065
   Log likelihood = -126.32608                     Pseudo R2         =     0.0983
```

Thursday September 5 11:12:10 2019   Page 9

datt7	Odds Ratio	Std. Err.	z	P>|z|	[95% Conf.	Interval]
cond						
Capsule	.4443503	.2622262	-1.37	0.169	.1397664	1.412695
CT colonography	.5453833	.3208585	-1.03	0.303	.1721571	1.727741
paid						
Yes	3.409665	1.811371	2.31	0.021	1.203689	9.65849
cond#paid						
Capsule#Yes	.2084142	.2547593	-1.28	0.200	.0189865	2.287761
CT colonography#Yes	.6946165	.6397408	-0.40	0.692	.1142325	4.223773
ethnicity						
BAME	8.265565	6.210024	2.81	0.005	1.895632	36.04052
cage						
65-69	1.03754	.4843493	0.08	0.937	.4155685	2.5904
70-74	1.229042	.6137913	0.41	0.680	.4618182	3.270861
female						
Yes	.7356454	.2819938	-0.80	0.423	.3470394	1.559403
idep						
1 marker	1.407705	.6223427	0.77	0.439	.591831	3.34831
2-3 markers	2.431126	1.412986	1.53	0.126	.7781867	7.595062
num						
Correct	.5895268	.2287447	-1.36	0.173	.2755658	1.261194
_cons	.0408934	.023251	-5.62	0.000	.0134178	.1246311

Note: **_cons** estimates baseline odds.

```
35 .
36 . //Difficulty with transport and deprivation interaction//
37 . logit datt9 i.cond##i.idep i.cage i.female i.ethnicity i.paid i.num if comp==1, or

Iteration 0:   log likelihood = -376.10599
Iteration 1:   log likelihood = -361.04686
Iteration 2:   log likelihood = -358.60263
Iteration 3:   log likelihood = -358.59978
Iteration 4:   log likelihood = -358.59978

Logistic regression                             Number of obs     =        954
                                                LR chi2(14)       =      35.01
                                                Prob > chi2       =     0.0015
Log likelihood = -358.59978                     Pseudo R2         =     0.0465
```

datt9	Odds Ratio	Std. Err.	z	P>|z|	[95% Conf.	Interval]
cond						
Capsule	.6689234	.1965032	-1.37	0.171	.3761206	1.189668
CT colonography	.5796323	.1791188	-1.76	0.078	.3163101	1.062165
idep						
1 marker	1.551238	.5872859	1.16	0.246	.738619	3.257889
2-3 markers	2.259239	1.1653	1.58	0.114	.8220878	6.208779
cond#idep						
Capsule#1 marker	1.046429	.5651208	0.08	0.933	.3630955	3.015773
Capsule#2-3 markers	2.351092	1.660775	1.21	0.226	.5888354	9.387398
CT colonography#1 marker	1.206273	.6866612	0.33	0.742	.3952813	3.681165
CT colonography#2-3 markers	1.584024	1.237581	0.59	0.556	.3425522	7.324815
cage						
65-69	.7956792	.189795	-0.96	0.338	.498539	1.269922
70-74	.6638411	.1776491	-1.53	0.126	.3928945	1.121637
female						
Yes	1.310302	.259888	1.36	0.173	.8882644	1.93286

For peer review only

Thursday September 5 11:12:11 2019 Page 10

ethnicity						
BAME	3.29872	2.040796	1.93	0.054	.9811572	11.09053
paid						
Yes	.8836746	.2128248	-0.51	0.608	.5511736	1.41676
num						
Correct	.7531362	.1499798	-1.42	0.155	.5097591	1.11271
_cons	.2011304	.0606306	-5.32	0.000	.1113996	.363138

Note: **_cons** estimates baseline odds.

```
38 .
39 . //Health problems and gender interaction//
40 . logit datt10 i.cond##i.female i.cage i.ethnicity i.idep i.paid i.num if comp==1, or

Iteration 0:   log likelihood = -302.59055
Iteration 1:   log likelihood = -293.95422
Iteration 2:   log likelihood = -289.70446
Iteration 3:   log likelihood = -289.54772
Iteration 4:   log likelihood = -289.54738
Iteration 5:   log likelihood = -289.54738

Logistic regression                             Number of obs     =        954
                                                LR chi2(12)       =      26.09
                                                Prob > chi2       =     0.0104
Log likelihood = -289.54738                     Pseudo R2         =     0.0431
```

datt10	Odds Ratio	Std. Err.	z	P>|z|	[95% Conf.	Interval]
cond						
Capsule	.8579749	.2803729	-0.47	0.639	.4521839	1.627924
CT colonography	.6854997	.245683	-1.05	0.292	.3395759	1.383814
female						
Yes	.7217563	.2606936	-0.90	0.367	.355584	1.465004
cond#female						
Capsule#Yes	.5703324	.3195649	-1.00	0.316	.1901903	1.710282
CT colonography#Yes	.6423506	.3828119	-0.74	0.458	.1997535	2.065617
cage						
65-69	.9063112	.2517804	-0.35	0.723	.5257834	1.56224
70-74	.8010558	.2442216	-0.73	0.467	.4407101	1.456037
ethnicity						
BAME	6.379531	3.809814	3.10	0.002	1.97905	20.56462
idep						
1 marker	1.348598	.3652431	1.10	0.269	.7931407	2.293057
2-3 markers	2.626326	.9337083	2.72	0.007	1.308369	5.271895
paid						
Yes	.9490002	.2590525	-0.19	0.848	.5557895	1.6204
num						
Correct	.9549643	.2155266	-0.20	0.838	.6135909	1.486262
_cons	.1505126	.0531107	-5.37	0.000	.0753726	.3005608

Note: **_cons** estimates baseline odds.

```
  Thursday September 5 11:12:11 2019   Page 11

41 .
42 . //Health problems and BAME interaction//
43 . logit datt10 i.cond##i.ethnicity i.cage i.female i.idep i.paid i.num if comp==1, or

   Iteration 0:   log likelihood = -302.59055
   Iteration 1:   log likelihood = -294.25034
   Iteration 2:   log likelihood = -292.74312
   Iteration 3:   log likelihood = -290.23031
   Iteration 4:   log likelihood = -289.55165
   Iteration 5:   log likelihood = -289.50732
   Iteration 6:   log likelihood = -289.50731

   Logistic regression                             Number of obs     =        954
                                                   LR chi2(12)       =      26.17
                                                   Prob > chi2       =     0.0102
   Log likelihood = -289.50731                     Pseudo R2         =     0.0432
```

datt10	Odds Ratio	Std. Err.	z	P>|z|	[95% Conf.	Interval]
cond						
Capsule	.6875368	.1819878	-1.42	0.157	.4092482	1.155061
CT colonography	.5434485	.158273	-2.09	0.036	.3070831	.9617471
ethnicity						
BAME	2.668504	3.201338	0.82	0.413	.2541573	28.01774
cond#ethnicity						
Capsule#BAME	2.369605	3.631279	0.56	0.573	.1175603	47.76299
CT colonography#BAME	5.661841	9.042489	1.09	0.278	.2474625	129.5406
cage						
65-69	.9066002	.2523885	-0.35	0.725	.5253512	1.564523
70-74	.7970324	.2438844	-0.74	0.458	.4375385	1.451896
female						
Yes	.5380308	.1284885	-2.60	0.009	.3369222	.859181
idep						
1 marker	1.332997	.3621348	1.06	0.290	.782679	2.270256
2-3 markers	2.5314	.8957882	2.62	0.009	1.26516	5.06496
paid						
Yes	.993958	.2734556	-0.02	0.982	.5796786	1.704311
num						
Correct	.962673	.2180531	-0.17	0.867	.6175538	1.500662
_cons	.1727472	.0568114	-5.34	0.000	.0906727	.3291136

```
   Note: _cons estimates baseline odds.

44 .
45 . //Health problems and deprivation interaction//
46 . logit datt10 i.cond##i.dep i.ethnicity i.cage i.female i.paid i.num if comp==1, or

   note: 3.cond#3.depr != 0 predicts failure perfectly
         3.cond#3.depr dropped and 1 obs not used

   note: 1.cond#3.depr identifies no observations in the sample
   note: 2.cond#3.depr omitted because of collinearity
   Iteration 0:   log likelihood = -302.48909
   Iteration 1:   log likelihood = -294.18407
   Iteration 2:   log likelihood = -289.98635
   Iteration 3:   log likelihood = -289.83164
   Iteration 4:   log likelihood = -289.83128
   Iteration 5:   log likelihood = -289.83128

   Logistic regression                             Number of obs     =        953
                                                   LR chi2(15)       =      25.32
                                                   Prob > chi2       =     0.0459
   Log likelihood = -289.83128                     Pseudo R2         =     0.0418
```

Thursday September 5 11:12:11 2019   Page 12

datt10	Odds Ratio	Std. Err.	z	P>|z|	[95% Conf.	Interval]
cond						
Capsule	.7088625	.2289895	-1.07	0.287	.3763497	1.335157
CT colonography	.5338427	.1891195	-1.77	0.076	.2666036	1.068958
depr						
1	1.375126	.612255	0.72	0.474	.5745892	3.290999
2	2.160367	1.200865	1.39	0.166	.7267371	6.422109
3	2.412483	2.730994	0.78	0.437	.2623504	22.18435
cond#depr						
Colonoscopy#3	1	(empty)				
Capsule#1	.8794388	.5557166	-0.20	0.839	.2548779	3.034444
Capsule#2	1.193254	1.049022	0.20	0.841	.2130191	6.684163
Capsule#3	1	(omitted)				
CT colonography#1	1.116122	.7644453	0.16	0.873	.2915502	4.272773
CT colonography#2	1.624588	1.464046	0.54	0.590	.2777523	9.502306
CT colonography#3	1	(empty)				
ethnicity						
BAME	6.434172	3.838078	3.12	0.002	1.998658	20.71319
cage						
65-69	.936774	.2627153	-0.23	0.816	.5406523	1.623124
70-74	.8234025	.2533449	-0.63	0.528	.4505199	1.504909
female						
Yes	.53614	.128135	-2.61	0.009	.3356178	.8564686
paid						
Yes	.9559571	.2622917	-0.16	0.870	.5583276	1.63677
num						
Correct	.9552938	.2165329	-0.20	0.840	.61263	1.489621
_cons	.1700755	.0577478	-5.22	0.000	.0874225	.3308722

Note: **_cons** estimates baseline odds.

```
47 .
48 . log close
      name:  <unnamed>
       log:  S:\FPHS_BSH_CancerComm\QQ PROJECTS 2012-17\BOWEL SCREENING PROJECT\Alternatives to
  log type:  smcl
 closed on:   5 Sep 2019, 11:08:22
```

For peer review only

STROBE Statement—checklist of items that should be included in reports of observational studies

	Item No	Recommendation
**Title and abstract**	1	(*a*) Indicate the study's design with a commonly used term in the title or the abstract CVW
		(*b*) Provide in the abstract an informative and balanced summary of what was done and what was found CVW
**Introduction**		
Background/rationale	2	Explain the scientific background and rationale for the investigation being reported CVW
Objectives	3	State specific objectives, including any prespecified hypotheses CVW
**Methods**		
Study design	4	Present key elements of study design early in the paper CVW
Setting	5	Describe the setting, locations, and relevant dates, including periods of recruitment, exposure, follow-up, and data collection CVW
Participants	6	(*a*) *Cohort study*—Give the eligibility criteria, and the sources and methods of selection of participants. Describe methods of follow-up<br>*Case-control study*—Give the eligibility criteria, and the sources and methods of case ascertainment and control selection. Give the rationale for the choice of cases and controls<br>*Cross-sectional study*—Give the eligibility criteria, and the sources and methods of selection of participants CVW
		(*b*) *Cohort study*—For matched studies, give matching criteria and number of exposed and unexposed<br>*Case-control study*—For matched studies, give matching criteria and the number of controls per case
Variables	7	Clearly define all outcomes, exposures, predictors, potential confounders, and effect modifiers. Give diagnostic criteria, if applicable cvw
Data sources/ measurement	8*	For each variable of interest, give sources of data and details of methods of assessment (measurement). Describe comparability of assessment methods if there is more than one group cvw
Bias	9	Describe any efforts to address potential sources of bias NA
Study size	10	Explain how the study size was arrived at NA, study was exploratory
Quantitative variables	11	Explain how quantitative variables were handled in the analyses. If applicable, describe which groupings were chosen and why CVW
Statistical methods	12	(*a*) Describe all statistical methods, including those used to control for confounding CVW
		(*b*) Describe any methods used to examine subgroups and interactions NA
		(*c*) Explain how missing data were addressed NA
		(*d*) *Cohort study*—If applicable, explain how loss to follow-up was addressed<br>*Case-control study*—If applicable, explain how matching of cases and controls was addressed<br>*Cross-sectional study*—If applicable, describe analytical methods taking account of sampling strategy CVW
		(*e*) Describe any sensitivity analyses NA

Continued on next page

For peer review only - http://bmjopen.bmj.com/site/about/guidelines.xhtml

**Results**		
Participants	13*	(a) Report numbers of individuals at each stage of study—eg numbers potentially eligible, examined for eligibility, confirmed eligible, included in the study, completing follow-up, and analysed CVW
		(b) Give reasons for non-participation at each stage NA, survey was anonymous
		(c) Consider use of a flow diagram CVW
Descriptive data	14*	(a) Give characteristics of study participants (eg demographic, clinical, social) and information on exposures and potential confounders CVW
		(b) Indicate number of participants with missing data for each variable of interest CVW
		(c) *Cohort study*—Summarise follow-up time (eg, average and total amount)CVW
Outcome data	15*	*Cohort study*—Report numbers of outcome events or summary measures over time
		Case-control study—Report numbers in each exposure category, or summary measures of exposure
		Cross-sectional study—Report numbers of outcome events or summary measures CVW
Main results	16	(*a*) Give unadjusted estimates and, if applicable, confounder-adjusted estimates and their precision (eg, 95% confidence interval). Make clear which confounders were adjusted for and why they were included CVW
		(*b*) Report category boundaries when continuous variables were categorized CVW
		(*c*) If relevant, consider translating estimates of relative risk into absolute risk for a meaningful time period
Other analyses	17	Report other analyses done—eg analyses of subgroups and interactions, and sensitivity analyses
**Discussion**		
Key results	18	Summarise key results with reference to study objectives CVW
Limitations	19	Discuss limitations of the study, taking into account sources of potential bias or imprecision. Discuss both direction and magnitude of any potential biasCVW
Interpretation	20	Give a cautious overall interpretation of results considering objectives, limitations, multiplicity of analyses, results from similar studies, and other relevant evidenceCVW
Generalisability	21	Discuss the generalisability (external validity) of the study resultsCVW
**Other information**		
Funding	22	Give the source of funding and the role of the funders for the present study and, if applicable, for the original study on which the present article is based CVW

*Give information separately for cases and controls in case-control studies and, if applicable, for exposed and unexposed groups in cohort and cross-sectional studies.

Note: An Explanation and Elaboration article discusses each checklist item and gives methodological background and published examples of transparent reporting. The STROBE checklist is best used in conjunction with this article (freely available on the Web sites of PLoS Medicine at http://www.plosmedicine.org/, Annals of Internal Medicine at http://www.annals.org/, and Epidemiology at http://www.epidem.com/). Information on the STROBE Initiative is available at www.strobe-statement.org.

For peer review only

BMJ Open

Preferences for different diagnostic modalities to follow up abnormal colorectal cancer screening results: a hypothetical vignette study

Journal:	*BMJ Open*
Manuscript ID	bmjopen-2019-035264.R1
Article Type:	Original research
Date Submitted by the Author:	22-Mar-2020

Complete List of Authors:	Kaushal, Aradhna; University College London, Behavioural Science and Health<br>Stoffel , Sandro; University College London, Business School; University of Aberdeen<br>Kerrison, Robert; University College London, Epidemiology and Public Health<br>von Wagner, Christian; UCL, Department of Epidemiology and Public Health

**Primary Subject Heading**:	Public health
Secondary Subject Heading:	Communication
Keywords:	PREVENTIVE MEDICINE, PUBLIC HEALTH, Endoscopy < GASTROENTEROLOGY

SCHOLARONE™ Manuscripts

I, the Submitting Author has the right to grant and does grant on behalf of all authors of the Work (as defined in the below author licence), an exclusive licence and/or a non-exclusive licence for contributions from authors who are: i) UK Crown employees; ii) where BMJ has agreed a CC-BY licence shall apply, and/or iii) in accordance with the terms applicable for US Federal Government officers or employees acting as part of their official duties; on a worldwide, perpetual, irrevocable, royalty-free basis to BMJ Publishing Group Ltd ("BMJ") its licensees and where the relevant Journal is co-owned by BMJ to the co-owners of the Journal, to publish the Work in this journal and any other BMJ products and to exploit all rights, as set out in our licence.

The Submitting Author accepts and understands that any supply made under these terms is made by BMJ to the Submitting Author unless you are acting as an employee on behalf of your employer or a postgraduate student of an affiliated institution which is paying any applicable article publishing charge ("APC") for Open Access articles. Where the Submitting Author wishes to make the Work available on an Open Access basis (and intends to pay the relevant APC), the terms of reuse of such Open Access shall be governed by a Creative Commons licence – details of these licences and which Creative Commons licence will apply to this Work are set out in our licence referred to above.

Other than as permitted in any relevant BMJ Author's Self Archiving Policies, I confirm this Work has not been accepted for publication elsewhere, is not being considered for publication elsewhere and does not duplicate material already published. I confirm all authors consent to publication of this Work and authorise the granting of this licence.

For peer review only

Preferences for different diagnostic modalities to follow up abnormal colorectal cancer screening results: a hypothetical vignette study

Aradhna Kaushal[1], Sandro T. Stoffel[1,2], Robert Kerrison[1], and Christian von Wagner[1]

[1] Research Department of Behavioural Science and Health, University College London

[2] Business School, University of Aberdeen

Corresponding author:

Christian von Wagner, Research Department of Behavioural Science and Health, University College London, 1-19 Torrington Place, London, WC1E 7HB

Email: c.wagner@ucl.ac.uk

Word Count: 3160/3000

Tables and Figures: 5/5

3/22/2020

For peer review only

For peer review only - http://bmjopen.bmj.com/site/about/guidelines.xhtml

Abstract

Objectives

In England, a significant proportion of people who take part in the national bowel cancer screening programme (BCSP) and have a positive faecal occult blood test (FOBt) result, do not attend follow-up colonoscopy (CC). The aim of this study was to investigate differences in intended participation in a follow-up investigation by diagnostic modality offered including (CC), computed tomography colonography (CTC), or capsule endoscopy (CE).

Setting

We performed a randomised online experiment with individuals who had previously completed a FOBt as part of the English BCSP.

Methods

Participants (n=953) were randomly allocated to receive one of three online vignettes asking participants to imagine they had received an abnormal FOBt result, and that they had been invited for a follow-up test. The follow-up test offered was either: CC (n=346), CTC (n=302), or CE (n=305). Participants were then asked how likely they were to have their allocated test or if they refused either of the other tests. Respondents were also asked to cite possible emotional and practical barriers to follow up testing. Multi-variable logistic regression models were used to investigate intentions.

Results

Intention to have the test was higher in the CTC group (96.7%) compared with the CC group (91.8%; Odds Ratio [OR]: 2.64; 95% Confidence Intervals [CI]: 1.22-5.73). CTC was

For peer review only

For peer review only - http://bmjopen.bmj.com/site/about/guidelines.xhtml

considered less “off-putting” (OR: 0.66, 95% CI: 0.46-0.94) and less uncomfortable compared to CC (OR: 0.51, 95%; CI: 0.34-0.77). For those who did not intend to have the test they were offered, CE (39.7%) or no investigation (34.5%) was preferable to CC (8.6%) or CTC (17.2%).

Conclusions

Alternative tests have the potential to increase attendance at diagnostic follow-up appointments.

Word count: 267/250

Strengths and Limitations

- Use of a vignette design allowed us to investigate attitudes towards hypothetical scenarios which would be more difficult to carry out in a real-world setting.
- The use of comprehension checks after presenting vignettes ensure that participants engage with materials and had an understanding of the tests they were presented.
- The study design presents a hypothetical scenario and so it is not possible to say if intentions would align with behaviours in real-life.
- We only provided brief descriptions of the test which may have biased intentions.
- The online panel used in the survey has a very small number of BAME participants and therefore may not be representative of the UK population.

For peer review only

For peer review only - http://bmjopen.bmj.com/site/about/guidelines.xhtml

Introduction

Colorectal cancer (CRC) is the 4th most common cancer in the United Kingdom (UK), accounting for 12% of all cancer diagnosis, and is the second leading cause of cancer death. [1, 2] Screening can reduce mortality and improve survival by detecting CRC at an earlier stage, when treatment is more likely to be successful. [3] In England, the national bowel cancer screening programme (BCSP) sends a screening test to all men and women aged 60 to 74 to be completed at home. The test is offered once every two years, and can detect small amounts of blood in the stool, which may be indicative of CRC. This test until recently was the 'faecal occult blood test' (FOBt) and was replaced with the more sensitive 'feacal immunochemical test' (FIT) in June 2019. Around 2% of people who complete a FOBt have a positive test result and are invited for further investigation, usually a colonoscopy (CC). Despite being at increased risk, approximately 14% of those with an abnormal test result fail to complete their diagnostic follow up. [4-7] There are a number of reasons why a CC in a small proportion of these patients would not be appropriate, such as clinical decisions based on frailty, or having had a recent CC outside the BSCP. [8, 9]

Previous research point towards a range of psychological and practical factors which may influence decisions to have colorectal cancer screening. [9, 11, 12] Plumb et al (2017) reviewed the medical records of patients of 170 patients to identify patient factors associated with non-attendance at CC after a positive FOBt and found the most cited reason was "unwillingness to complete the test". [9] For some patients, this was due to anxiety about pain, and risks but in the majority of cases, the reason was unspecified. This is supported by a recent systematic review on incomplete diagnostic follow-up after an abnormal CRC screening result which identified perception of pain as the most commonly cited reason in addition to

For peer review only - http://bmjopen.bmj.com/site/about/guidelines.xhtml

82 ‘embarrassment’ and ‘being too busy’. [13] Consequently, for a vast majority of this group of patients there is a strong need to develop patient or system level interventions to remove modifiable barriers to investigating the cause of their FOB test result. [10]

85 Some of these barriers may be directly or indirectly related to the nature of CC. CC involves inserting a long flexible tube with a tiny camera on the end into the anus in order to examine the bowel. If polyps (small, potentially cancerous growths) are found, they are removed during the procedure. The test requires the patient to restrict their diet, take a strong laxative, and arrange for someone to take them home afterwards due to drowsiness caused by a sedative. After the procedure, most people will need to rest for the remainder of the day. [14]

91 Considering the practical and emotional barriers which may inhibit someone from accepting the offer of further investigation via CC, it has been suggested that offering alternative tests, such as CTC or capsule endoscopy, may be more acceptable to patients. [15-17] These tests differ in terms of preparation, time in hospital and level of perceived invasiveness. Both of these tests, as with CC, require the patient to restrict their diet and take a laxative. In CTC, a small tube is used to inflate the bowel after which x-rays are used to take images of the bowel after which most people are able to resume their daily activities. [18] In capsule endoscopy, the patient ingests a capsule containing a small camera which takes photos inside the bowel and transmits them wirelessly to a receiver worn by the patient. [19] Patients are able to carry on with their normal activity and the capsule is passed through the body after eight hours. For both of these tests, a CC is usually recommended to remove any polyps if they are found.

102 CTC and CE could be offered individuals who decline to have a CC after a positive FOBt, in the expectation that providing individuals with alternative choices increases their feeling of autonomy and intrinsic motivation. [20-22] While offering choice seems promising, there exists no evidence that offering more than one screening test influences adherence and patient

For peer review only - http://bmjopen.bmj.com/site/about/guidelines.xhtml

satisfaction.[23] Although CTC and CE are not currently endorsed as a screening methods, it is possible that offering alternative tests for those who do stop engaging with the screening programme may increase intentions to have further investigations.

The present study aimed to evaluate whether offering different screening tests, such as CTC, or CE, increases intentions to attend for further investigation, when compared to offering a CC.

Methods

Design

We performed a randomised online experiment with a survey company called: 'Dynata' (formerly Survey Sampling International and Research Now). Potentially eligible men and women were invited to participate in the online experiment by Dynata, who invited them from their panel if they were: 1) aged 60-74 years and 2) lived in England. Potentially eligible men and women were informed that they would be rewarded with 'Dynata points', which they could exchange for money, or a donation to a charity, if they complete the survey.

Individuals who agreed to participate in the survey were asked about their screening history, which was used to filter ineligible adults. Only individuals who had completed a FOBt previously, and received a normal result, were eligible to participate in the survey. We excluded participants who had previously had a CC, a previous diagnosis of colorectal cancer or had parts of their bowel removed. Once eligibility to participate had been established using these criteria, participants were then randomly allocated to one of three experimental conditions in which they were asked to read a hypothetical vignette (see Supplementary Appendix 1).

For peer review only - http://bmjopen.bmj.com/site/about/guidelines.xhtml

Depending on the condition individuals were allocated to, the vignette asked them to imagine that their next FOBt result was abnormal, and they were being invited for a follow-up test consisting of either CC, a CTC, or a CE. Each vignette included a some images of the test, a short description about what happens before, during and after the test, and any risks associated with the offered test. After reading the information, participants were required to successfully answer a 'comprehension check question' about the test (what happens during the test, how long it takes, and what happens afterwards), to ensure they had understood the information. If answered incorrectly, the information about the test was presented again and the participant is provided with another opportunity to answer the question. Once the participant answered the comprehension check question correctly, they were asked about their intention to have the follow-up test. Details of the comprehension check questions can be found in the Supplementary Appendix 1.

Measures

Intention to have follow-up test: On answering the comprehension check question correctly, participants were asked to indicate their intention to book an appointment (*"Considering all the information presented above, would you take up the offer of this test?"*), using a four-point Likert scale, with response options: '*definitely not*', '*probably not*', '*probably, yes*' and '*definitely, yes*'. [24, 25] These responses were dichotomised ('*probably, yes*' or '*definitely, yes*' vs. '*probably not*' or '*definitely not*') due to the relative lack of 'probably not' and 'definitely not' responses (see Supplementary Appendix 2).

Barriers for follow-up test: After indicating their intentions, participants were then asked to indicate how they felt about potential barriers for not wanting the tests ("*Please read each statement and select how strongly you agree or disagree with it?*"), using a four-point Likert scale, with response options: *'strongly agree', 'slightly agree', 'slightly disagree'* and

For peer review only - http://bmjopen.bmj.com/site/about/guidelines.xhtml

'strongly disagree'. The barriers featured six emotional and four practical items, and were derived from previous surveys. [9, 26] The six emotional barrier items were: "*The preparation for the test (restricted diet and strong laxative) puts me off", "The test looks like it would be uncomfortable", "I would be embarrassed about taking the test", "I would worry about the risks associated with the test", "I would be afraid of getting an abnormal result" and "Doing the test would make me worry more about bowel cancer"*. The four practical barrier items were: *"I would not have time to do the test", "I have other problems to worry about", "It would be difficult to arrange transport to the hospital"* and *"I have other health problems that are more important"*. These responses were dichotomised ('*strongly agree'* or *'slightly agree'* vs '*slightly disagree'* or *'strongly disagree'*) due to the relative lack of 'strongly agree' responses (see Supplementary Appendix 2).

Preference of disinclined study participants. Study participants who indicated that they would not (probably or definitely) have the offered follow-up test were presented with information about the other two tests and asked which of the three tests they would prefer ("*Which of the two tests would you prefer to have*?"), with response options *"colonoscopy", "capsule", "CT colonography"* and *"none of them"*.

Numeracy skills. Numeracy skills were assessed by the question: 'Which of the following numbers represents the biggest risk of getting a disease?', with answer options '1/10', '1/100', '1/1000' and 'I don't know'. This measure was adapted from Lipkus and colleagues, who previously validated the question as a measure for numeracy skills. [15]

Socio-demographic variables. Details of the participant's age, gender, ethnicity, employment status, education, car and home ownership were collected at the end of the survey. Ethnicity data were recoded as 'White' (White British or Other White Background) and "BAME" (Black, Asian and minority ethnic) due to the small numbers in each group.

For peer review only

The three variables on education, car and home ownership were used to calculate a proxy measure for socio-economic deprivation. One point was given to a participant if their household did not own a car or van, if they had no formal qualifications and if they did not own their own home. [27-29] Scores, therefore, ranged from 0 to 3, with high scores indicating higher levels of deprivation.

Sample size calculation

Sample size of this study was calculated prior to data collection based on the results of a soft launch. We calculated that we needed approximately 300 participants per condition to detect differences of at least 5 percentage points in proportion of intenders effect size between any of the three conditions, with a power of 80% and an alpha value of 0.05. [30]

Statistical analysis

Our primary outcome was intention to have the offered follow-up test after exposure to the allocated vignette. Our secondary outcomes were the responses to the perceived emotional and practical barrier questions. Descriptive statistics were used to report the socio-demographic characteristics of the study population. Uni-variable and multi-variable logistic regression models were used to investigate the effect of offering alternative follow-up tests on participants’ intentions to have further investigations, as well as their perceived emotional and practical barriers of the test offered. Covariates that were included in the regression models were age, gender, ethnicity, deprivation score, employment status and numeracy. All statistical analysis was conducted using Stata/SE (version 15.1). [31] The survey, data and Stata codes for the experiment are available via Open Science Framework: https://osf.io/fx69t/?view_only=46ba2185c26940a89c19bde28da40a9c.

For peer review only

For peer review only - http://bmjopen.bmj.com/site/about/guidelines.xhtml

Results

Participants

In total, 1,926 adults responded to the online invitation. Of these, 481 (25.0%) were excluded due to their age or medical history (i.e. previous bowel cancer diagnosis or removal of part of the bowel). A further 372 (19.3%) were excluded as they had never been invited for, or completed, a FOBt, and an additional 46 (2.4%) were excluded as they had previously received an abnormal FOBt result. Of the remaining 1,027 adults who were eligible for inclusion, 953 (92.8%) completed the survey (see Figure 1 for an overview of participants through the study).

- Figure 1 here -

As shown in Table 1, around half of participants were male (54.5%), of a White ethnic background (98.6%), not in paid employment (75.1%), had a formal education (62.7%), owned car (87.1%), and owned a house (87.4%).

For peer review only - http://bmjopen.bmj.com/site/about/guidelines.xhtml

213

214 **Table 1. Socio-demographic characteristics of the study population (N=953)**

	Colonoscopy condition (N=305)		Capsule endoscopy condition (N=346)		CT Colonography condition (N=302)		Overall (N=953)	
**Age**								
60-64	98	(32.1%)	94	(27.2%)	109	(36.1%)	301	(31.6%)
65-69	123	(40.3%)	125	(36.1%)	109	(36.1%)	357	(37.5%)
70-74	84	(27.5%)	127	(36.7%)	84	(27.8%)	295	(31.0%)
**Gender**								
Male	151	(49.5%)	201	(58.1%)	167	(55.3%)	519	(54.5%)
Female	154	(50.5%)	145	(41.9%)	135	(44.7%)	434	(45.5%)
**Ethnicity**								
White	301	(98.7%)	341	(98.6%)	298	(98.7%)	940	(98.6%)
BMAE	4	(1.3%)	5	(1.4%)	4	(1.3%)	13	(1.4%)
**Paid employment**								
No	221	(72.5%)	260	(75.1%)	235	(77.8%)	716	(75.1%)
Yes	84	(27.5%)	86	(24.9%)	67	(22.2%)	237	(24.9%)
**Education**								
No A levels	119	(39.0%)	125	(36.1%)	112	(37.4%)	356	(37.4%)
A levels or higher	186	(61.0%)	221	(63.9%)	190	(62.6%)	597	(62.6%)
**Car ownership**								
No	36	(11.8%)	46	(13.3%)	41	(13.6%)	123	(12.9%)
Yes	269	(88.2%)	300	(86.7%)	261	(86.4%)	830	(87.1%)
**House ownership**								
No	44	(14.4%)	46	(13.3%)	30	(9.9%)	120	(12.6%)
Yes	261	(85.6%)	300	(86.7%)	272	(90.1%)	833	(87.4%)
**Individual social deprivation**								
0 markers	223	(73.1%)	242	(69.9%)	221	(73.2%)	686	(72.0%)
1 marker	58	(19.0%)	80	(23.1%)	63	(20.8%)	201	(21.1%)
2-3 markers	24	(7.9%)	24	(6.9%)	18	(5.9%)	66	(6.9%)
**Numeracy question**								
Wrong	163	(53.4%)	180	(52.0%)	151	(50.0%)	494	(51.8%)
Correct	142	(46.6%)	166	(48.0%)	151	(50.0%)	459	(48.2%)

215 *p-value refers to Chi-Square test of independence if not stated differently

216 The three demographic questions on education, car and house ownership were used to calculate a proxy
217 measure for socio-economic deprivation. One point was given to an individual if their household did not have a
218 car or van, if they had no formal qualifications and if they did not own their own home [1, 2]. Scores, therefore,

For peer review only

ranged from 0 to 3, with high scores indicating higher levels of social deprivation. Due to low frequencies, we collapsed the two groups that either had a score of 2 or 3 into one group called 2-3 markers.

Intention to have the allocated test

A large majority (93.7%) intended to have the allocated test, although this was graded by ethnicity (61.5% vs 94.4%) and deprivation (2-3 markers: 87.9% vs. 0 markers: 95.5%). The proportion of intenders was highest among those who were offered CTC, followed by those who were offered capsule, and finally those who were offered CC (96.7% vs. 93.4% vs. 91.8% respectively, $\chi 2$=(2, N=953) =6.64, p=0.036).

In the multi-variable model, participants remained more likely to accept further investigation if the offered test was CTC compared to CC, after adjusting for co-variates (Table 2; OR: 2.64, 95% CI: 1.22–5.73, p=0.014). Offering CE did not significantly increase intentions compared to CC (OR: 1.28, 95% CI: 0.69–2.35, p=0.432). The multi-variable analysis also revealed that intention to undergo follow-up tests was lower among BAME groups than White ethnic groups (OR: 0.09, 95% CI: 0.03–0.29, p<0.001), as well as more deprived individuals (1 marker: OR: 0.45, 95% CI: 0.25–0.84, p=0.012; 2-3 markers: OR: 0.34, 95% CI: 0.15–0.79, p=0.012).

Preferences of disinclined study participants

In total, 58 (6.1%) study participants did not intend to have their offered follow-up test. Independently from the initial offer, most disinclined study participants would either choose CE (N=23, 39.7%) or none of the tests (N=20, 34.5%). A Fisher's exact test demonstrated that preferences were not influenced by the initial random allocation (p=0.806).

For peer review only - http://bmjopen.bmj.com/site/about/guidelines.xhtml

242 –

For peer review only

For peer review only - http://bmjopen.bmj.com/site/about/guidelines.xhtml

243 Table 2. Association between test offered and wanting to have the test (N=953)

	Unadjusted model		Adjusted model	
	OR	95% CI	OR	95% CI
**Condition**				
Colonoscopy	Ref.		Ref.	
Capsule endoscopy	1.25	0.70 - 2.26	1.28	0.69 - 2.35
CT colonography	2.61	**1.23 - 5.53***	2.64	**1.22 - 5.73***
**Age**				
60-64			Ref.	
65-69			0.70	0.36 - 1.37
70-74			1.42	0.63 - 3.22
**Gender**				
Male			Ref.	
Female			1.09	0.62 - 1.91
**Ethnicity**				
White			Ref.	
BAME			0.09	**0.03 - 0.29****
**Deprivation**				
0 makers			Ref.	
1 marker			0.45	**0.25 - 0.84***
2-3 markers			0.34	**0.15 - 0.79***
**Paid employment**				
No			Ref.	
Yes			0.87	0.45 - 1.67
**Numeracy task**				
Wrong			Ref.	
Correct			1.58	0.89 - 2.78

244 Logistic regression models presented; OR: Odds ratio; CI: Confidence intervals; BAME: Black, Asian and Minority

245 Ethnic; * $p<0.05$; ** $p<0.01$

For peer review only

For peer review only - http://bmjopen.bmj.com/site/about/guidelines.xhtml

Perceived emotional barriers

Those who were randomised to CTC were less likely to agree that the test is off-putting (OR: 0.66, 95% CI: 0.46–0.94, p=0.022) or uncomfortable (OR: 0.51, 95% CI: 0.34–0.77, p=0.001) than those who were offered a CC. Participants who were offered CE as a follow-up test were less likely to perceive the test as uncomfortable (OR: 0.11: 95% CI: 0.07–0.16, p<0.001), embarrassing (OR: 0.33, 95% CI: 0. 23–0.48, p<0.001), or creating worries about the risks associated with the test (OR: 0.70, 95% CI: 0.51–0.97, p=0.031) than those who were offered a CC (Table 3).

The table also shows that women were more likely to perceive the offered tests as off-putting (OR: 1.41, 95% CI: 1.06–1.88, p=0.018), embarrassing (OR: 1.83, 95% CI: 1.36–2.47, p<0.001) and causing worries about the risks (OR: 1.46, 95% CI: 1.11–1.91, p=0.007) and results of the test (OR: 1.57, 95% CI: 1.20–2.05, p=0.001).

Higher deprivation was associated with worrying about the risks associated with the test (OR: 1.50, 95% CI: 1.08-2.08, p=0.014) and numeracy was positively associated with being afraid of the results (OR: 1.32, 95% CI: 1.01-1.72, p=0.39). Sub-group analysis did not reveal any significant interactions between these demographic variables and test allocation (Supplementary Appendix 3).

- Table 3 here –

For peer review only - http://bmjopen.bmj.com/site/about/guidelines.xhtml

For peer review only

For peer review only - http://bmjopen.bmj.com/site/about/guidelines.xhtml

Table 3. Association between test offered and emotional barrier items (N=953).

	Off-putting		Uncomfortable		Embarrassing		Worry about risks of test		Afraid of results		Worry about cancer	
	OR	95% CI	OR	95% CI	OR	95% CI	OR	95% CI	OR	95% CI	OR	95% CI
**Condition**												
Colonoscopy	Ref.		Ref.		Ref.		Ref.		Ref.		Ref.	
Capsule endoscopy	0.75	0.53 - 1.047	0.11	**0.07 - 0.16****	0.33	**0.23 - 0.48****	0.70	**0.51 - 0.97***	0.75	0.54 - 1.03	0.82	0.59 - 1.13
CT colonography	0.66	**0.47 - 0.94***	0.51	**0.34 - 0.77****	0.72	0.51 - 1.02	0.73	0.52 - 1.02	0.92	0.66 - 1.28	1.15	0.83 - 1.60
**Age**												
60-64	Ref.		Ref.		Ref.		Ref.		Ref.		Ref.	
65-69	0.99	0.69 - 1.41	0.91	0.62 - 1.32	0.81	0.56 - 1.16	0.85	0.61 - 1.19	0.68	**0.48 - 0.94***	0.67	**0.48 - 0.93***
70-74	1.05	0.72 - 1.54	0.89	0.60 - 1.33	0.88	0.59 - 1.31	0.88	0.62 - 1.27	0.71	0.49 - 1.01	0.74	0.52 - 1.06
**Gender**												
Male	Ref.		Ref.		Ref.		Ref.		Ref.		Ref.	
Female	1.41	**1.06 - 1.88***	1.03	0.76 - 1.39	1.83	**1.36 - 2.47****	1.46	**1.11 - 1.91****	1.57	**1.20 - 2.05****	1.05	0.801 - 1.38
**Ethnicity**												
White	Ref.		Ref.		Ref.		Ref.		Ref.		Ref.	
BAME	2.22	0.73 - 6.75	1.22	0.33 - 4.55	1.19	0.34 - 4.19	2.02	0.66 - 6.17	3.97	0.86 - 18.33	1.76	0.58 - 5.34
**Deprivation**												
0 markers	Ref.		Ref.		Ref.		Ref.		Ref.		Ref.	
1 marker	1.06	0.75 - 1.50	1.36	0.93 - 1.97	0.99	0.69 - 1.43	1.50	**1.09 - 2.08***	1.36	0.97 - 1.89	1.34	0.97 - 1.86
2-3 markers	1.43	0.84 - 2.44	0.65	0.36 - 1.15	1.29	0.73 - 2.26	1.26	0.75 - 2.14	0.68	0.41 - 1.14	0.99	0.58 - 1.680
**Paid employment**												
No	Ref.		Ref.		Ref.		Ref.		Ref.		Ref.	
Yes	0.99	0.70 - 1.40	1.45	1.00 - 2.11	1.41	1.00 - 2.01	1.11	0.80 - 1.53	1.19	0.86 - 1.65	1.01	0.73 - 1.40
**Numeracy task**												
Wrong	Ref.		Ref.		Ref.		Ref.		Ref.		Ref.	
Correct	1.03	0.79 - 1.37	0.88	0.66 - 1.19	1.13	0.84 - 1.52	1.08	0.83 - 1.42	1.32	**1.01 - 1.72***	1.22	0.94 - 1.59

Logistic regression models presented; OR: Odds ratio; CI: Confidence intervals; BAME: Black, Asian and Minority Ethnic; * $p<0.05$; ** $p<0.01$

For peer review only

For peer review only - http://bmjopen.bmj.com/site/about/guidelines.xhtml

Perceived practical barriers

Study participants who were offered CE, instead of CC, were less likely to think that they wouldn't have the time to do the test (OR: 0.27, 95% CI: 0.10–0.70, p=0.007). There were no other differences in practical barriers by test offered (See table 4). Health problems were cited as a barrier by participants with a higher number of deprivation markers (OR: 2.54, 95% CI: 1.27-5.07 , p=0.009), those with a BAME background (OR: 6.45, 95% CI:2.01-20.73 , p=0.002) and by men (OR: 1.85, 95% CI: 2.94-1.16, p=0.009). Those from BAME groups (OR:9.37, 95% CI:2.23-39.36, p=0.002), and those who were in paid in employment (OR:2.42, 95% CI:1.09-5.38 , p=0.03) were more likely to report not having enough time as a barrier to a follow-up investigation. Participants with a higher number of deprivation markers were also more likely to cite difficulties with transport (OR: 3.52 , 95% CI:1.96-6.31, p<0.001) as a barrier to having a follow-up investigation. Sub-group analysis did not reveal any significant interactions between these demographic variables and test allocation (Supplementary Appendix 3) .

–

For peer review only - http://bmjopen.bmj.com/site/about/guidelines.xhtml

Table 4. Association between study condition and practical barrier items (n=953).

	No time		Other problems		Difficulties with transport		Health problems	
	OR	95% CI	OR	95% CI	OR	95% CI	OR	95% CI
**Condition**								
Colonoscopy	Ref.		Ref.		Ref.		Ref.	
Capsule endoscopy	0.27	**0.10 - .70****	0.75	0.52 - 1.09	0.76	0.48 - 1.18	0.70	0.42 - 1.17
CT colonography	0.46	0.19 - 1.09	0.80	0.55 - 1.18	0.64	0.40 - 1.04	0.58	0.33 - 1.01
**Age**								
60-64	Ref.		Ref.		Ref.		Ref.	
65-69	1.06	0.43 - 2.63	0.91	0.62 - 1.35	0.77	0.49 - 1.23	0.92	0.53 - 1.58
70-74	1.28	0.48 - 3.39	1.03	0.68 - 1.55	0.65	0.39 - 1.10	0.80	0.44 - 1.46
**Gender**								
Male	Ref.		Ref.		Ref.		Ref.	
Female	0.73	0.35 - 1.55	1.18	0.86 - 1.61	1.33	0.91 - 1.96	0.54	**0.34 - 0.86****
**Ethnicity**								
White	Ref.		Ref.		Ref.		Ref.	
BAME	9.37	**2.23 - 39.36****	2.28	0.73 - 7.10	3.32	0.99 - 11.13	6.45	**2.01 - 20.73****
**Deprivation**								
0 markers	Ref.		Ref.		Ref.		Ref.	
1 marker	1.40	0.59 - 3.32	1.11	0.76 - 1.61	1.65	**1.06 - 2.58***	1.35	0.79 - 2.29
2-3 markers	2.41	0.78 - 7.49	1.37	0.77 - 2.44	3.52	**1.96 - 6.31****	2.54	**1.27 - 5.07****
**Paid employment**								
No	Ref.		Ref.		Ref.		Ref.	
Yes	2.42	**1.09 - 5.38***	0.97	0.66 - 1.42	0.88	0.55 - 1.41	0.95	0.56 - 1.63
**Numeracy task**								
Wrong	Ref.		Ref.		Ref.		Ref.	
Correct	0.60	0.28 - 1.26	0.83	0.61 - 1.14	0.74	0.50 - 1.09	0.96	0.61 - 1.49

Logistic regression models presented; OR: Odds ratio; CI: Confidence intervals; BAME: Black, Asian and Minority Ethnic;
* *p*<0.05; ** *p*<0.01

For peer review only - http://bmjopen.bmj.com/site/about/guidelines.xhtml

Discussion

This study investigated whether offering alternative follow-up tests increases intention to undergo further investigation in the context of CRC screening. The results of our study show that, while the vast majority of individuals would accept any test, offering CTC instead of CC may increase acceptance of follow-up tests. Findings from our study suggest that this is due to individuals perceiving CTC as less ‘off-putting’ and ‘uncomfortable’ than CC. No differences were found in intention between those randomised to CE compared to CC despite CE appearing less uncomfortable, less embarrassing, causing less worry about the risks associated with the test, and taking test time. However, for the small number of participants who did not intend to take the offered test, CE appeared to be the preferred test when presented with all options.

The findings from this study are supported by a trial in France by Pioche et al. (2018) which randomised patients who had an abnormal FOBt result, but who refused CC, to be invited for either CE or CTC. [32] They found that CTC was more appealing than CE (7.4% vs. 5.0% having the investigation) but no differences were found in clinical outcomes. The authors concluded that those who refuse CC are difficult to recruit into the screening programme and that simply offering a different test is not effective. This trial was carried out in France and may not be generalisable to a UK population and the NHS screening programme. Importantly, as the study focused on patients who had already refused CC it looked at preferences in a pre-selected sample. This is reflected in the small sample size with less than 50 participants in each comparison group which makes it difficult to say for certain that offering alternative tests would not be of any benefit at a population level.

For peer review only - http://bmjopen.bmj.com/site/about/guidelines.xhtml

Our study also observed a lower likelihood of ethnic minorities to accept any follow-up test. This is supported by a recent systematic review of characteristics of incomplete diagnostic follow-up after a positive bowel screening test identified non-White participants as less likely to attend in 9 out of 10 studies. [13] Analysis of barriers suggest that other health problems and not having enough time may be additional contributing factors to intention. Previous research has identified embarrassment as a potential barrier to colorectal cancer screening, although this was not found in this study. [33] We also found that not having enough time, and difficulties arranging transport are barriers to screening for people in employment, those with a higher level of deprivation and those from a BAME background. This is consistent with evidence from an American study, which showed that the location of the CC appointment and the availability of evening and weekend appointments are important factors limiting uptake. [34] These findings should be interpreted with care due to the very small number of BAME participants in the entire sample and the heterogeneity of the group. Future research should aim to recruit a larger and more representative BAME sample so findings can be presented for each ethnic group.

While our study provides support that individuals perceive follow-up tests differently, we only offered one test at random. Future research should present all three tests to participants in a random order to analyse whether offering individuals the choice between different tests increases intentions. [23] This could also include investigation of other diagnostic tests such as stool DNA tests. [35]

Strengths and limitations

Our study has several limitations. Firstly, our study used hypothetical scenarios and non-representative online study samples. Although the vignettes were developed in collaboration with clinicians, it was not possible in the space available to fully explain the level of burden

For peer review only

For peer review only - http://bmjopen.bmj.com/site/about/guidelines.xhtml

associated with each test which may have biased responses. Furthermore, the experiments lacked behavioural validation, in that they only measured intentions and choice in a hypothetical setting. The next step would be to test the effect of offering alternative follow-up tests under more ecologically valid conditions in a randomised controlled trial.

A strength of our study is that we used comprehension checks to ensure that all study participants sufficiently engaged with the provided information about the follow-up test.

Conclusions

While the results from this randomised online experiment show a high acceptance of the current practice of the NHS Bowel Screening Programme to invite individuals who have an abnormal FOBt result for a follow-up CC, our study also provides some evidence that offering CTC, instead of CC, may increase the total number of participants undertaking a follow-up test. Although CTC and CE are not currently endorsed as a screening modalites, our study provides some evidence that for high-risk and hard-to-engage groups, such as those who have a positive FOBt but refuse follow-up, offering alternative tests may increase intentions to have engage with medical professionals and receive appropriate care.

For peer review only - http://bmjopen.bmj.com/site/about/guidelines.xhtml

Figure legend:

Figure 1. Participant flow diagram

Abbreviations

CRC: colorectal cancer; FOBt: faecal occult blood test; CC: Colonoscopy; CTC: CT Colonography; CE: Capsule Endoscopy; OR: Odds Ratio; 95% CI: 95% Confidence Interval

Declarations

Ethics and consent to participate

This research project was approved by UCL Research ethics committee (approval number 14687/001). All participants were asked to give explicit written consent for their data to be used and published as part of this research project before taking part in this study.

Consent for publication

Not applicable.

Competing interests

The authors have declared that no competing interests exist.

Supporting information

Supporting file contains demographic characteristics of the study samples, additional description of results and all outcome measures of the experimental studies.

For peer review only

For peer review only - http://bmjopen.bmj.com/site/about/guidelines.xhtml

Patient and Public Involvement

The design of this study followed a direct question posed by representatives of Public Health England and was informed by previous research on this topic and close collaboration with experts involved in administering relevant tests (see acknowledgments).

Availability of data and materials

All data files and materials are publicly available via the Open Science Framework and can be accessed at https://osf.io/fx69t/?view_only=46ba2185c26940a89c19bde28da40a9c.

Funding

This report presents independent research commissioned and funded by the National Institute for Health Research (NIHR) Policy Research Programme, conducted through the Policy Research Unit in Cancer Awareness, Screening and Early Diagnosis, PR-PRU-1217-21601. The views expressed are those of the authors and not necessarily those of the NIHR, the Department of Health and Social Care or its arm's length bodies, or other Government Departments.

Authors' contributions

AK, STS and CW developed the study concept. All authors contributed to the study design. AK, STS and CW performed the data analysis and interpretation. AK and STS drafted the manuscript, RK and CW provided critical revisions. All authors approved the final version of the manuscript for submission.

Acknowledgements

We would like to acknowledge the contribution to the study materials by Professor Owen Epstein, Tim Rayne and Dr. Andrew Plumb.

For peer review only

For peer review only - http://bmjopen.bmj.com/site/about/guidelines.xhtml

References

1. Cancer Research UK. Bowel cancer incidence statistics, https://www.cancerresearchuk.org/health-professional/cancer-statistics/statistics-by-cancer-type/bowel-cancer/incidence (2018, accessed 24th October 2018).

2. Cancer Research UK. Bowel cancer mortality statistics, https://www.cancerresearchuk.org/health-professional/cancer-statistics/statistics-by-cancer-type/bowel-cancer/mortality#ref- (2018, accessed 29th Aug 2019).

3. Logan RF, Patnick J, Nickerson C, et al. Outcomes of the Bowel Cancer Screening Programme (BCSP) in England after the first 1 million tests. *Gut* 2012; 61: 1439-1446.

4. Hirst Y, Stoffel S, Baio G, et al. Uptake of the English Bowel (Colorectal) Cancer Screening Programme: an update 5 years after the full roll-out. *European Journal of Cancer* 2018; 103: 267-273.

5. Kearns B, Whyte S, Seaman HE, et al. Factors associated with completion of bowel cancer screening and the potential effects of simplifying the screening test algorithm. 2016; 114: 327.

6. Beshara A, Ahoroni M, Comanester D, et al. Association Between Time to Colonoscopy After a Positive Guaiac Fecal Test Result and Risk of Colorectal Cancer and Advanced Stage Disease at Diagnosis. *International journal of cancer* 2019.

7. Kaalby L, Rasmussen M, Zimmermann-Nielsen E, et al. Time to colonoscopy, cancer probability, and precursor lesions in the Danish colorectal cancer screening program. *Clinical Epidemiology* 2019; 11: 659.

8. Paynter S. A pilot study exploring primary care involvement in re-engaging patients with the Bowel Cancer Screening Programme who have tested positive on screening but failed to complete colonoscopy. Poster, Public Health England,, 2015.

9. Plumb AA, Ghanouni A, Rainbow S, et al. Patient factors associated with non-attendance at colonoscopy after a positive screening faecal occult blood test. *Journal of medical screening* 2017; 24: 12-19.

10. Selby K, Baumgartner C, Levin TR, et al. Interventions to improve follow-up of positive results on fecal blood tests: a systematic review. *Annals of internal medicine* 2017; 167: 565-575.

11. Honein-AbouHaidar GN, Kastner M, Vuong V, et al. Systematic review and meta-study synthesis of qualitative studies evaluating facilitators and barriers to participation in colorectal cancer screening. *Cancer Epidemiol Biomarkers Prev*. 2016; 25: 907-917.

For peer review only - http://bmjopen.bmj.com/site/about/guidelines.xhtml

12. Power E, Miles A, Von Wagner C, et al. Uptake of colorectal cancer screening: system, provider and individual factors and strategies to improve participation. 2009; 5: 1371-1388.

13. Dalton AJJoPH. Incomplete diagnostic follow-up after a positive colorectal cancer screening test: a systematic review. *J Public Health (Oxf)*. 2017; 40: e46-e58.

14. Carlson CM, Kirby KA, Casadei MA, et al. Lack of follow-up after fecal occult blood testing in older adults: inappropriate screening or failure to follow up? *Archives of internal medicine* 2011; 171: 249-256.

15. Groth S, Krause H, Behrendt R, et al. Capsule colonoscopy increases uptake of colorectal cancer screening. *BMC gastroenterology* 2012; 12: 80.

16. Rex DK and Lieberman DA. A survey of potential adherence to capsule colonoscopy in patients who have accepted or declined conventional colonoscopy. *Journal of clinical gastroenterology* 2012; 46: 691-695.

17. Pooler BD, Baumel MJ, Cash BD, et al. Screening CT colonography: multicenter survey of patient experience, preference, and potential impact on adherence. *American journal of roentgenology* 2012; 198: 1361-1366.

18. Kim DH, Pickhardt PJ, Taylor AJ, et al. CT colonography versus colonoscopy for the detection of advanced neoplasia. *New England journal of medicine* 2007; 357: 1403-1412.

19. Iddan G, Meron G, Glukhovsky A, et al. Wireless capsule endoscopy. *Nature* 2000; 405: 417.

20. Katz I and Assor A. When choice motivates and when it does not. *Educational Psychology Review* 2007; 19: 429.

21. Patall EA, Cooper H and Robinson JC. The effects of choice on intrinsic motivation and related outcomes: a meta-analysis of research findings. *Psychological bulletin* 2008; 134: 270.

22. Zuckerman M, Porac J, Lathin D, et al. On the importance of self-determination for intrinsically-motivated behavior. *Personality and social psychology bulletin* 1978; 4: 443-446.

23. Partin MR, Powell AA, Burgess DJ, et al. Bringing an organizational perspective to the optimal number of colorectal cancer screening options debate. *Journal of general internal medicine* 2012; 27: 376-380.

24. Stoffel ST, Goodwin M, Sieverding M, et al. Testing verbal quantifiers for social norms messages in cancer screening: evidence from an online experiment. *BMC public health* 2019; 19: 658.

25. Stoffel ST, Hirst Y, Ghanouni A, et al. Testing active choice for screening practitioner's gender in endoscopy among disinclined women: An online experiment. *Journal of medical screening* 2019; 26: 98-103.

For peer review only

For peer review only - http://bmjopen.bmj.com/site/about/guidelines.xhtml

26. Smith S, McGregor L, Raine R, et al. Inequalities in cancer screening participation: examining differences in perceived benefits and barriers. *Psycho‐Oncology* 2016; 25: 1168-1174.

27. McGregor LM, von Wagner C, Vart G, et al. The impact of supplementary narrative-based information on colorectal cancer screening beliefs and intention. *BMC cancer* 2015; 15: 162.

28. Von Wagner C, Bonello B, Stoffel S, et al. Barriers to bowel scope (flexible sigmoidoscopy) screening: a comparison of non-responders, active decliners and non-attenders. *BMC public health* 2018; 18: 1161.

29. Wardle J, McCaffery K, Nadel M, et al. Socioeconomic differences in cancer screening participation: comparing cognitive and psychosocial explanations. *Social science & medicine* 2004; 59: 249-261.

30. Cohen J. *Statistical power analysis for the behavioral sciences*. Routledge, 2013.

31. StataCorp. Stata Statistical Software: Release 16. College Station, TX: StataCorp LLC, 2019.

32. Pioche M, Ganne C, Gincul R, et al. Colon capsule versus computed tomography colonography for colorectal cancer screening in patients with positive fecal occult blood test who refuse colonoscopy: a randomized trial. *Endoscopy* 2018; 50: 761-769.

33. Robb KA, Solarin I, Power E, et al. Attitudes to colorectal cancer screening among ethnic minority groups in the UK. *BMC public health* 2008; 8: 34.

34. O'Malley A, Beaton E, Yabroff K, et al. Patient and provider barriers to colorectal cancer screening in the primary care safety-net. 2004; 39: 56-63.

35. Imperiale TF, Ransohoff DF, Itzkowitz SH, et al. Multitarget stool DNA testing for colorectal-cancer screening. *New England Journal of Medicine* 2014; 370: 1287-1297.

For peer review only

For peer review only - http://bmjopen.bmj.com/site/about/guidelines.xhtml

STROBE Statement—Checklist of items that should be included in reports of ***cross-sectional studies***

	Item No	Recommendation	Page number (line number)
**Title and abstract**	1	(*a*) Indicate the study's design with a commonly used term in the title or the abstract	2 (24)
		(*b*) Provide in the abstract an informative and balanced summary of what was done and what was found	2-3 (27-40)
**Introduction**			
Background/rationale	2	Explain the scientific background and rationale for the investigation being reported	5-6 (100-106)
Objectives	3	State specific objectives, including any prespecified hypotheses	6 (107-109)
**Methods**			
Study design	4	Present key elements of study design early in the paper	6-7 (126-137)
Setting	5	Describe the setting, locations, and relevant dates, including periods of recruitment, exposure, follow-up, and data collection	6 (112-125)
Participants	6	(*a*) Give the eligibility criteria, and the sources and methods of selection of participants	6 (118-125)
Variables	7	Clearly define all outcomes, exposures, predictors, potential confounders, and effect modifiers. Give diagnostic criteria, if applicable	7-9 (139-178)
Data sources/ measurement	8*	For each variable of interest, give sources of data and details of methods of assessment (measurement). Describe comparability of assessment methods if there is more than one group	7-9 (139-178)
Bias	9	Describe any efforts to address potential sources of bias	6-7 (126-137)
Study size	10	Explain how the study size was arrived at	9 (180-183)
Quantitative variables	11	Explain how quantitative variables were handled in the analyses. If applicable, describe which groupings were chosen and why	7-9 (139-178)
Statistical methods	12	(*a*) Describe all statistical methods, including those used to control for confounding	9 (185-197)
		(*b*) Describe any methods used to examine subgroups and interactions	12 (247-249)
		(*c*) Explain how missing data were addressed	N/A
		(*d*) If applicable, describe analytical methods taking account of sampling strategy	N/A
		(*e*) Describe any sensitivity analyses	N/A
**Results**			
Participants	13*	(a) Report numbers of individuals at each stage of study—eg numbers potentially eligible, examined for eligibility, confirmed eligible, included in the study, completing follow-up, and analysed	10 (200-206)

For peer review only - http://bmjopen.bmj.com/site/about/guidelines.xhtml
For peer review only

		(b) Give reasons for non-participation at each stage	See Figure 1
		(c) Consider use of a flow diagram	See Figure 1
Descriptive data	14*	(a) Give characteristics of study participants (eg demographic, clinical, social) and information on exposures and potential confounders	10 (208-210) and Table 1.
		(b) Indicate number of participants with missing data for each variable of interest	N/A
Outcome data	15*	Report numbers of outcome events or summary measures	10 (213-217)
Main results	16	(*a*) Give unadjusted estimates and, if applicable, confounder-adjusted estimates and their precision (eg, 95% confidence interval). Make clear which confounders were adjusted for and why they were included	11 (219-226) and Table 2.
		(*b*) Report category boundaries when continuous variables were categorized	See Table 1 caption
		(*c*) If relevant, consider translating estimates of relative risk into absolute risk for a meaningful time period	N/A
Other analyses	17	Report other analyses done—eg analyses of subgroups and interactions, and sensitivity analyses	12 (247-249) and Supplementary Appendix 3.
**Discussion**			
Key results	18	Summarise key results with reference to study objectives	13 (266-275)
Limitations	19	Discuss limitations of the study, taking into account sources of potential bias or imprecision. Discuss both direction and magnitude of any potential bias	14-15 (307-315)
Interpretation	20	Give a cautious overall interpretation of results considering objectives, limitations, multiplicity of analyses, results from similar studies, and other relevant evidence	13-14 (276-300
Generalisability	21	Discuss the generalisability (external validity) of the study results	14-15 (307-315)
**Other information**			
Funding	22	Give the source of funding and the role of the funders for the present study and, if applicable, for the original study on which the present article is based	17 (349-354)

*Give information separately for exposed and unexposed groups.

Note: An Explanation and Elaboration article discusses each checklist item and gives methodological background and published examples of transparent reporting. The STROBE checklist is best used in conjunction with this article (freely available on the Web sites of PLoS Medicine at http://www.plosmedicine.org/, Annals of Internal Medicine at http://www.annals.org/, and Epidemiology at http://www.epidem.com/). Information on the STROBE Initiative is available at www.strobe-statement.org.

For peer review only - http://bmjopen.bmj.com/site/about/guidelines.xhtml

```
      Thursday September 5 11:12:06 2019   Page 1

                                                     ___  ____  ____  ____  ____(R)
                                                    /__    /   ____/   /   ____/
                                                   ___/   /   /___/   /   /___/
                                                     Statistics/Data Analysis

-------------------------------------------------------------------------------------------
      name:  <unnamed>
       log:  S:\FPHS_BSH_CancerComm\QQ PROJECTS 2012-17\BOWEL SCREENING PROJECT\Alternatives to
  log type:  smcl
 opened on:   5 Sep 2019, 11:08:18

  1 . use "S:\FPHS_BSH_CancerComm\QQ PROJECTS 2012-17\BOWEL SCREENING PROJECT\Alternatives to Colon

  2 .
  3 . //Intention and BAME interaction//
  4 . logit int2 i.cond##i.ethnicity i.cage i.female i.idep i.paid i.num if comp==1, or

note: 1.cond#1.ethnicity != 0 predicts success perfectly
      1.cond#1.ethnicity dropped and 4 obs not used

note: 3.cond#1.ethnicity omitted because of collinearity
Iteration 0:   log likelihood = -218.29837
Iteration 1:   log likelihood = -215.64434
Iteration 2:   log likelihood = -206.51414
Iteration 3:   log likelihood = -199.21258
Iteration 4:   log likelihood = -197.68211
Iteration 5:   log likelihood = -197.37916
Iteration 6:   log likelihood = -197.37691
Iteration 7:   log likelihood = -197.37691

Logistic regression                             Number of obs     =        949
                                                LR chi2(11)       =      41.84
                                                Prob > chi2       =     0.0000
Log likelihood = -197.37691                     Pseudo R2         =     0.0958

-------------------------------------------------------------------------------------
                 int2 | Odds Ratio   Std. Err.      z    P>|z|     [95% Conf. Interval]
----------------------+--------------------------------------------------------------
                 cond |
              Capsule |   1.440134   .4558544     1.15   0.249     .7744031    2.678175
      CT colonography |   3.164895   1.327974     2.75   0.006     1.390591    7.203097
                      |
            ethnicity |
                 BAME |   .0257632   .0289846    -3.25   0.001     .0028403    .2336873
                      |
       cond#ethnicity |
     Colonoscopy#BAME |          1  (empty)
         Capsule#BAME |   1.525905   2.263561     0.28   0.776     .0833368    27.93948
 CT colonography#BAME |          1  (omitted)
                      |
                 cage |
                65-69 |   .7060353   .2438926    -1.01   0.314     .3587465    1.389521
                70-74 |   1.402867   .5943763     0.80   0.424     .6114676    3.218544
                      |
               female |
                  Yes |    1.06484   .3075379     0.22   0.828     .6045717    1.875517
                      |
                 idep |
             1 marker |   .4684608    .148785    -2.39   0.017     .2513786    .8730079
         2-3 markers  |   .3456216   .1480694    -2.48   0.013     .1492566    .8003283
                      |
                 paid |
                  Yes |   .7472369   .2478844    -0.88   0.380     .3900185    1.431632
                      |
                  num |
              Correct |   1.487324   .4351085     1.36   0.175     .8382839    2.638882
                _cons |   14.26229   6.135926     6.18   0.000     6.137408    33.14312
-------------------------------------------------------------------------------------
Note: _cons estimates baseline odds.
```

For peer review only

Thursday September 5 11:12:08 2019 Page 2

```
  5 .
  6 . //Intention and deprivation interaction//
  7 . logit int2 i.cond##i.idep  i.cage i.female i.ethnicity i.paid i.num if comp==1, or

Iteration 0:   log likelihood = -218.55008
Iteration 1:   log likelihood = -203.06951
Iteration 2:   log likelihood = -199.19849
Iteration 3:   log likelihood = -199.10343
Iteration 4:   log likelihood = -199.10307
Iteration 5:   log likelihood = -199.10307

Logistic regression                             Number of obs     =        953
                                                LR chi2(14)       =      38.89
                                                Prob > chi2       =     0.0004
Log likelihood = -199.10307                     Pseudo R2         =     0.0890
```

int2	Odds Ratio	Std. Err.	z	P>|z|	[95% Conf.	Interval]
cond						
Capsule	1.740958	.7370709	1.31	0.190	.7593019	3.99174
CT colonography	3.305797	1.748944	2.26	0.024	1.172046	9.324114
idep						
1 marker	.5428541	.2653282	-1.25	0.211	.208279	1.414884
2-3 markers	.8346543	.6618163	-0.23	0.820	.1764289	3.948604
cond#idep						
Capsule#1 marker	.7549797	.5310209	-0.40	0.689	.1902111	2.99664
Capsule#2-3 markers	.1872543	.1874193	-1.67	0.094	.0263318	1.33163
CT colonography#1 marker	.6383686	.5497961	-0.52	0.602	.1180246	3.452792
CT colonography#2-3 markers	.4718537	.6540561	-0.54	0.588	.0311833	7.139919
cage						
65-69	.6643002	.2293366	-1.18	0.236	.3376788	1.306848
70-74	1.385225	.5810532	0.78	0.437	.6087953	3.151879
female						
Yes	1.135806	.3270864	0.44	0.658	.6459178	1.997243
ethnicity						
BAME	.0868887	.0536264	-3.96	0.000	.0259188	.2912809
paid						
Yes	.8567131	.2863368	-0.46	0.644	.4449803	1.649415
num						
Correct	1.533339	.44838	1.46	0.144	.8644292	2.719864
_cons	12.76679	5.550371	5.86	0.000	5.44529	29.93245

Note: **_cons** estimates baseline odds.

```
  8 .
  9 . //Off-putting and gender interaction//
 10 . logit datt1 i.cond##i.female i.cage i.ethnicity i.idep i.paid i.num if comp==1, or

Iteration 0:   log likelihood = -575.64795
Iteration 1:   log likelihood = -567.00146
Iteration 2:   log likelihood = -566.95168
Iteration 3:   log likelihood = -566.95168

Logistic regression                             Number of obs     =        954
                                                LR chi2(12)       =      17.39
                                                Prob > chi2       =     0.1354
Log likelihood = -566.95168                     Pseudo R2         =     0.0151
```

For peer review only

Thursday September 5 11:12:08 2019 Page 3

datt1	Odds Ratio	Std. Err.	z	P>|z|	[95% Conf.	Interval]
cond						
Capsule	.5994014	.1453158	-2.11	0.035	.3726978	.9640035
CT colonography	.602084	.1528489	-2.00	0.046	.3660713	.9902584
female						
Yes	1.147654	.2795688	0.57	0.572	.7119644	1.849967
cond#female						
Capsule#Yes	1.561034	.5367613	1.30	0.195	.7956584	3.062653
CT colonography#Yes	1.193689	.4305356	0.49	0.624	.5886849	2.42047
cage						
65-69	.9965692	.1806268	-0.02	0.985	.6985996	1.42163
70-74	1.047513	.2039252	0.24	0.812	.7152387	1.534149
ethnicity						
BAME	2.271933	1.290318	1.44	0.148	.7463898	6.91553
idep						
1 marker	1.061183	.1891052	0.33	0.739	.7483492	1.504792
2-3 markers	1.403437	.3834878	1.24	0.215	.8214909	2.397634
paid						
Yes	.9865023	.1755985	-0.08	0.939	.6959585	1.39834
num						
Correct	1.039599	.1512154	0.27	0.789	.7817247	1.38254
_cons	.4533808	.1097497	-3.27	0.001	.2821064	.7286403

Note: **_cons** estimates baseline odds.

```
11 .
12 . //Embarrasing and gender interaction//
13 . logit datt3 i.cond##i.female i.cage i.ethnicity i.idep i.paid i.num if comp==1, or

Iteration 0:   log likelihood = -559.79953
Iteration 1:   log likelihood = -526.78936
Iteration 2:   log likelihood = -526.08874
Iteration 3:   log likelihood = -526.08791
Iteration 4:   log likelihood = -526.08791

Logistic regression                         Number of obs     =        954
                                            LR chi2(12)       =      67.42
                                            Prob > chi2       =     0.0000
Log likelihood = -526.08791                 Pseudo R2         =     0.0602
```

datt3	Odds Ratio	Std. Err.	z	P>|z|	[95% Conf.	Interval]
cond						
Capsule	.393843	.1049377	-3.50	0.000	.2336277	.6639295
CT colonography	.6117832	.158551	-1.90	0.058	.3681276	1.016709
female						
Yes	1.802571	.4375261	2.43	0.015	1.120175	2.900674
cond#female						
Capsule#Yes	.6950697	.2652284	-0.95	0.340	.32902	1.468366
CT colonography#Yes	1.382337	.4910231	0.91	0.362	.6890595	2.773136
cage						
65-69	.8078209	.1503881	-1.15	0.252	.5608556	1.163534
70-74	.889448	.1795823	-0.58	0.562	.5987705	1.321237
ethnicity						
BAME	1.155396	.7401264	0.23	0.822	.3292044	4.055045
idep						
1 marker	.9886713	.1860287	-0.06	0.952	.6837378	1.429599

For peer review only

Thursday September 5 11:12:08 2019 Page 4

2-3 markers	1.281844	.3718801	0.86	0.392	.7259228	2.263498
paid						
Yes	1.439032	.2589492	2.02	0.043	1.011346	2.047583
num						
Correct	1.112985	.1693064	0.70	0.482	.8260481	1.499593
_cons	.420774	.1040708	-3.50	0.000	.2591312	.6832476

Note: **_cons** estimates baseline odds.

```
14 .
15 . //Worry about test risk and gender interaction//
16 . logit datt4 i.cond##i.female i.cage i.ethnicity i.idep i.paid i.num if comp==1, or

Iteration 0:   log likelihood = -625.38047
Iteration 1:   log likelihood = -610.79631
Iteration 2:   log likelihood = -610.75012
Iteration 3:   log likelihood = -610.75012

Logistic regression                             Number of obs     =        954
                                                LR chi2(12)       =      29.26
                                                Prob > chi2       =     0.0036
Log likelihood = -610.75012                     Pseudo R2         =     0.0234
```

datt4	Odds Ratio	Std. Err.	z	P>|z|	[95% Conf.	Interval]
cond						
Capsule	.8625152	.1965071	-0.65	0.516	.5518706	1.34802
CT colonography	.6402238	.1565886	-1.82	0.068	.396407	1.034004
female						
Yes	1.574237	.372587	1.92	0.055	.9899417	2.503402
cond#female						
Capsule#Yes	.626443	.2078761	-1.41	0.159	.3269057	1.20044
CT colonography#Yes	1.312932	.4499751	0.79	0.427	.6706774	2.570221
cage						
65-69	.8532505	.1461122	-0.93	0.354	.6099795	1.193542
70-74	.8931162	.1645699	-0.61	0.540	.6223884	1.281606
ethnicity						
BAME	1.94679	1.10931	1.17	0.242	.6372233	5.947667
idep						
1 marker	1.494679	.2483308	2.42	0.016	1.079261	2.069995
2-3 markers	1.271042	.3415635	0.89	0.372	.7506177	2.15229
paid						
Yes	1.121261	.1875352	0.68	0.494	.8078669	1.556228
num						
Correct	1.065082	.1471823	0.46	0.648	.8123747	1.396398
_cons	.532697	.1239152	-2.71	0.007	.3376564	.8403991

Note: **_cons** estimates baseline odds.

```
17 .
```

For peer review only

```
  Thursday September 5 11:12:09 2019   Page 5

18 . //Worry about test risk and deprivation interaction//
19 . logit datt4 i.cond##i.idep i.cage i.female i.ethnicity i.paid i.num if comp==1, or

   Iteration 0:   log likelihood = -625.38047
   Iteration 1:   log likelihood = -609.00743
   Iteration 2:   log likelihood = -608.97148
   Iteration 3:   log likelihood = -608.97148

   Logistic regression                             Number of obs     =        954
                                                   LR chi2(14)       =      32.82
                                                   Prob > chi2       =     0.0031
   Log likelihood = -608.97148                     Pseudo R2         =     0.0262
```

datt4	Odds Ratio	Std. Err.	z	P>|z|	[95% Conf.	Interval]
cond						
Capsule	.8545529	.1688906	-0.80	0.426	.5801105	1.25883
CT colonography	.8738928	.1757055	-0.67	0.503	.5892709	1.295989
idep						
1 marker	2.773085	.8485567	3.33	0.001	1.522291	5.051596
2-3 markers	1.35632	.5985531	0.69	0.490	.5711117	3.221092
cond#idep						
Capsule#1 marker	.3593903	.1484572	-2.48	0.013	.159938	.8075719
Capsule#2-3 markers	1.287068	.7999347	0.41	0.685	.3806846	4.351488
CT colonography#1 marker	.4862063	.2070739	-1.69	0.090	.2110067	1.120327
CT colonography#2-3 markers	.5373902	.3794284	-0.88	0.379	.1346767	2.144307
cage						
65-69	.8475212	.1459977	-0.96	0.337	.6046707	1.187906
70-74	.8790054	.1626208	-0.70	0.486	.6116663	1.263189
female						
Yes	1.454996	.2032422	2.68	0.007	1.106525	1.91321
ethnicity						
BAME	2.084474	1.191957	1.28	0.199	.6796058	6.39346
paid						
Yes	1.107485	.1857581	0.61	0.543	.7971979	1.538543
num						
Correct	1.074747	.1491767	0.52	0.604	.8187623	1.410764
_cons	.4918955	.1081869	-3.23	0.001	.3196396	.7569813

```
   Note: _cons estimates baseline odds.

20 .
21 . //Afraid of results risk and age group interaction//
22 . logit datt5 i.cond##i.cage i.female i.ethnicity i.idep i.paid i.num if comp==1, or

   Iteration 0:   log likelihood = -649.10164
   Iteration 1:   log likelihood =   -626.968
   Iteration 2:   log likelihood = -626.80181
   Iteration 3:   log likelihood = -626.80112
   Iteration 4:   log likelihood = -626.80112

   Logistic regression                             Number of obs     =        954
                                                   LR chi2(14)       =      44.60
                                                   Prob > chi2       =     0.0000
   Log likelihood = -626.80112                     Pseudo R2         =     0.0344
```

For peer review only

Thursday September 5 11:12:09 2019 Page 6

datt5	Odds Ratio	Std. Err.	z	P>|z|	[95% Conf.	Interval]
cond						
Capsule	.9487913	.2971592	-0.17	0.867	.5135425	1.752932
CT colonography	.8040911	.2395326	-0.73	0.464	.4484756	1.441689
cage						
65-69	.6737809	.1962447	-1.36	0.175	.3807139	1.192446
70-74	.7619691	.2441478	-0.85	0.396	.4066287	1.427831
cond#cage						
Capsule#65-69	.8464529	.3448479	-0.41	0.682	.3809082	1.880985
Capsule#70-74	.618933	.2636364	-1.13	0.260	.2685788	1.426315
CT colonography#65-69	1.124773	.4530285	0.29	0.770	.5107682	2.476887
CT colonography#70-74	1.360613	.5960542	0.70	0.482	.5765568	3.210902
female						
Yes	1.579452	.217962	3.31	0.001	1.205152	2.070003
ethnicity						
BAME	3.903997	3.066424	1.73	0.083	.8373884	18.20087
idep						
1 marker	1.369841	.2341368	1.84	0.066	.9798976	1.91496
2-3 markers	.6881451	.1825012	-1.41	0.159	.4091994	1.157244
paid						
Yes	1.1609	.1946665	0.89	0.374	.835719	1.61261
num						
Correct	1.318004	.1794388	2.03	0.043	1.009323	1.721087
_cons	1.307052	.3353875	1.04	0.297	.7904531	2.161274

Note: **_cons** estimates baseline odds.

```
23 .
24 . //Afraid of results risk and gender interaction//
25 . logit datt5 i.cond##i.female i.cage i.ethnicity i.idep i.paid i.num if comp==1, or

Iteration 0:   log likelihood = -649.10164
Iteration 1:   log likelihood = -628.77429
Iteration 2:   log likelihood = -628.62076
Iteration 3:   log likelihood = -628.62007
Iteration 4:   log likelihood = -628.62007

Logistic regression                             Number of obs     =        954
                                                LR chi2(12)       =      40.96
                                                Prob > chi2       =     0.0000
Log likelihood = -628.62007                     Pseudo R2         =     0.0316
```

datt5	Odds Ratio	Std. Err.	z	P>|z|	[95% Conf.	Interval]
cond						
Capsule	.7984076	.1756849	-1.02	0.306	.5187078	1.228928
CT colonography	.9635103	.2206762	-0.16	0.871	.6150389	1.50942
female						
Yes	1.706078	.413164	2.21	0.027	1.061358	2.742432
cond#female						
Capsule#Yes	.8715473	.286892	-0.42	0.676	.4571896	1.661444
CT colonography#Yes	.8956024	.3066038	-0.32	0.747	.457838	1.751938
cage						
65-69	.6722562	.1145439	-2.33	0.020	.4813943	.9387906
70-74	.7060271	.1287507	-1.91	0.056	.4938519	1.00936
ethnicity						
BAME	3.943815	3.079102	1.76	0.079	.8537837	18.21735

For peer review only

Thursday September 5 11:12:10 2019   Page 7

idep						
1 marker	1.355455	.2308021	1.79	0.074	.9708356	1.892452
2-3 markers	.6852833	.1808773	-1.43	0.152	.408507	1.149584
paid						
Yes	1.188009	.1987038	1.03	0.303	.8559524	1.648883
num						
Correct	1.321628	.1794319	2.05	0.040	1.01285	1.72454
_cons	1.28226	.2930399	1.09	0.277	.8193087	2.006802

Note: **_cons** estimates baseline odds.

```
26 .
27 . //Worry about cancer and gender interaction//
28 . logit datt6 i.cond##i.female i.cage i.ethnicity i.idep i.paid i.num if comp==1, or

Iteration 0:   log likelihood = -638.84517
Iteration 1:   log likelihood = -629.17662
Iteration 2:   log likelihood = -629.16658
Iteration 3:   log likelihood = -629.16658

Logistic regression                          Number of obs     =        954
                                             LR chi2(12)       =      19.36
                                             Prob > chi2       =     0.0803
Log likelihood = -629.16658                  Pseudo R2         =     0.0152
```

datt6	Odds Ratio	Std. Err.	z	P>|z|	[95% Conf.	Interval]
cond						
Capsule	.8870318	.1995902	-0.53	0.594	.5707042	1.378692
CT colonography	1.139612	.2635305	0.57	0.572	.7243027	1.793056
female						
Yes	1.116424	.2651117	0.46	0.643	.7009684	1.778115
cond#female						
Capsule#Yes	.8280574	.2740884	-0.57	0.569	.4328239	1.584198
CT colonography#Yes	1.021262	.3416495	0.06	0.950	.5301261	1.967412
cage						
65-69	.6718341	.1127177	-2.37	0.018	.4835605	.9334119
70-74	.7439943	.1335862	-1.65	0.100	.5232798	1.057804
ethnicity						
BAME	1.742306	.9845806	0.98	0.326	.5755841	5.274
idep						
1 marker	1.33407	.2194894	1.75	0.080	.9663482	1.84172
2-3 markers	.9924304	.269328	-0.03	0.978	.583041	1.689278
paid						
Yes	1.015197	.1667701	0.09	0.927	.7357331	1.400813
num						
Correct	1.215624	.1644366	1.44	0.149	.9325193	1.584677
_cons	.6736522	.153843	-1.73	0.084	.4305717	1.053964

Note: **_cons** estimates baseline odds.

For peer review only

```
      Thursday September 5 11:12:10 2019   Page 8

 29 .
 30 . //No time about BAME interaction//
 31 . logit datt7 i.cond##i.ethnicity i.cage i.female i.idep i.paid i.num if comp==1, or

    note: 1.cond#1.ethnicity != 0 predicts failure perfectly
          1.cond#1.ethnicity dropped and 4 obs not used

    note: 3.cond#1.ethnicity omitted because of collinearity
    Iteration 0:   log likelihood = -139.95813
    Iteration 1:   log likelihood = -131.43249
    Iteration 2:   log likelihood =    -126.68
    Iteration 3:   log likelihood = -123.90107
    Iteration 4:   log likelihood =  -123.2927
    Iteration 5:   log likelihood = -123.26786
    Iteration 6:   log likelihood = -123.26785

    Logistic regression                             Number of obs     =        950
                                                    LR chi2(11)       =      33.38
                                                    Prob > chi2       =     0.0005
    Log likelihood = -123.26785                     Pseudo R2         =     0.1193

    ----------------------------------------------------------------------------------------
                     datt7 | Odds Ratio   Std. Err.      z    P>|z|     [95% Conf. Interval]
    -----------------------+----------------------------------------------------------------
                      cond |
                  Capsule  |    .225937   .1167816    -2.88   0.004     .0820391    .6222342
         CT colonography  |   .3317475   .1608767    -2.28   0.023     .1282414     .858197
                           |
                 ethnicity |
                     BAME  |   71.17544   79.73479     3.81   0.000     7.920736    639.5798
                           |
            cond#ethnicity |
        Colonoscopy#BAME  |          1  (empty)
            Capsule#BAME  |   .2360667   .3916317    -0.87   0.384     .0091391    6.097719
    CT colonography#BAME  |          1  (omitted)
                           |
                      cage |
                    65-69  |   .9844457   .4644365    -0.03   0.973     .3904959    2.481801
                    70-74  |   1.270358   .6398335     0.48   0.635     .4733764    3.409147
                           |
                    female |
                      Yes  |   .7283684    .280484    -0.82   0.410     .3424253    1.549303
                           |
                      idep |
                 1 marker  |   1.327865   .5964158     0.63   0.528     .5505965    3.202392
             2-3 markers  |   2.433038   1.419385     1.52   0.127     .7754885    7.633479
                           |
                      paid |
                      Yes  |   3.022953   1.249684     2.68   0.007     1.344455    6.796991
                           |
                       num |
                  Correct  |   .6374225   .2473719    -1.16   0.246     .2979147    1.363839
                    _cons  |   .0482849   .0262249    -5.58   0.000     .0166533    .1399981
    ----------------------------------------------------------------------------------------
    Note: _cons estimates baseline odds.

 32 .
 33 . //No time about employment interaction//
 34 . logit datt7 i.cond##i.paid i.ethnicity i.cage i.female i.idep i.num if comp==1, or

    Iteration 0:   log likelihood =  -140.0949
    Iteration 1:   log likelihood = -129.40018
    Iteration 2:   log likelihood = -126.37467
    Iteration 3:   log likelihood =  -126.3263
    Iteration 4:   log likelihood = -126.32608
    Iteration 5:   log likelihood = -126.32608

    Logistic regression                             Number of obs     =        954
                                                    LR chi2(12)       =      27.54
                                                    Prob > chi2       =     0.0065
    Log likelihood = -126.32608                     Pseudo R2         =     0.0983
```

For peer review only

Thursday September 5 11:12:10 2019   Page 9

datt7	Odds Ratio	Std. Err.	z	P>|z|	[95% Conf.	Interval]
cond						
Capsule	.4443503	.2622262	-1.37	0.169	.1397664	1.412695
CT colonography	.5453833	.3208585	-1.03	0.303	.1721571	1.727741
paid						
Yes	3.409665	1.811371	2.31	0.021	1.203689	9.65849
cond#paid						
Capsule#Yes	.2084142	.2547593	-1.28	0.200	.0189865	2.287761
CT colonography#Yes	.6946165	.6397408	-0.40	0.692	.1142325	4.223773
ethnicity						
BAME	8.265565	6.210024	2.81	0.005	1.895632	36.04052
cage						
65-69	1.03754	.4843493	0.08	0.937	.4155685	2.5904
70-74	1.229042	.6137913	0.41	0.680	.4618182	3.270861
female						
Yes	.7356454	.2819938	-0.80	0.423	.3470394	1.559403
idep						
1 marker	1.407705	.6223427	0.77	0.439	.591831	3.34831
2-3 markers	2.431126	1.412986	1.53	0.126	.7781867	7.595062
num						
Correct	.5895268	.2287447	-1.36	0.173	.2755658	1.261194
_cons	.0408934	.023251	-5.62	0.000	.0134178	.1246311

Note: **_cons** estimates baseline odds.

```
35 .
36 . //Difficulty with transport and deprivation interaction//
37 . logit datt9 i.cond##i.idep i.cage i.female i.ethnicity i.paid i.num if comp==1, or

Iteration 0:   log likelihood = -376.10599
Iteration 1:   log likelihood = -361.04686
Iteration 2:   log likelihood = -358.60263
Iteration 3:   log likelihood = -358.59978
Iteration 4:   log likelihood = -358.59978

Logistic regression                             Number of obs     =        954
                                                LR chi2(14)       =      35.01
                                                Prob > chi2       =     0.0015
Log likelihood = -358.59978                     Pseudo R2         =     0.0465
```

datt9	Odds Ratio	Std. Err.	z	P>|z|	[95% Conf.	Interval]
cond						
Capsule	.6689234	.1965032	-1.37	0.171	.3761206	1.189668
CT colonography	.5796323	.1791188	-1.76	0.078	.3163101	1.062165
idep						
1 marker	1.551238	.5872859	1.16	0.246	.738619	3.257889
2-3 markers	2.259239	1.1653	1.58	0.114	.8220878	6.208779
cond#idep						
Capsule#1 marker	1.046429	.5651208	0.08	0.933	.3630955	3.015773
Capsule#2-3 markers	2.351092	1.660775	1.21	0.226	.5888354	9.387398
CT colonography#1 marker	1.206273	.6866612	0.33	0.742	.3952813	3.681165
CT colonography#2-3 markers	1.584024	1.237581	0.59	0.556	.3425522	7.324815
cage						
65-69	.7956792	.189795	-0.96	0.338	.498539	1.269922
70-74	.6638411	.1776491	-1.53	0.126	.3928945	1.121637
female						
Yes	1.310302	.259888	1.36	0.173	.8882644	1.93286

For peer review only

Thursday September 5 11:12:11 2019 Page 10

ethnicity						
BAME	3.29872	2.040796	1.93	0.054	.9811572	11.09053
paid						
Yes	.8836746	.2128248	-0.51	0.608	.5511736	1.41676
num						
Correct	.7531362	.1499798	-1.42	0.155	.5097591	1.11271
_cons	.2011304	.0606306	-5.32	0.000	.1113996	.363138

Note: **_cons** estimates baseline odds.

```
38 .
39 . //Health problems and gender interaction//
40 . logit datt10 i.cond##i.female i.cage i.ethnicity i.idep i.paid i.num if comp==1, or

Iteration 0:   log likelihood = -302.59055
Iteration 1:   log likelihood = -293.95422
Iteration 2:   log likelihood = -289.70446
Iteration 3:   log likelihood = -289.54772
Iteration 4:   log likelihood = -289.54738
Iteration 5:   log likelihood = -289.54738

Logistic regression                             Number of obs     =        954
                                                LR chi2(12)       =      26.09
                                                Prob > chi2       =     0.0104
Log likelihood = -289.54738                     Pseudo R2         =     0.0431
```

datt10	Odds Ratio	Std. Err.	z	P>|z|	[95% Conf.	Interval]
cond						
Capsule	.8579749	.2803729	-0.47	0.639	.4521839	1.627924
CT colonography	.6854997	.245683	-1.05	0.292	.3395759	1.383814
female						
Yes	.7217563	.2606936	-0.90	0.367	.355584	1.465004
cond#female						
Capsule#Yes	.5703324	.3195649	-1.00	0.316	.1901903	1.710282
CT colonography#Yes	.6423506	.3828119	-0.74	0.458	.1997535	2.065617
cage						
65-69	.9063112	.2517804	-0.35	0.723	.5257834	1.56224
70-74	.8010558	.2442216	-0.73	0.467	.4407101	1.456037
ethnicity						
BAME	6.379531	3.809814	3.10	0.002	1.97905	20.56462
idep						
1 marker	1.348598	.3652431	1.10	0.269	.7931407	2.293057
2-3 markers	2.626326	.9337083	2.72	0.007	1.308369	5.271895
paid						
Yes	.9490002	.2590525	-0.19	0.848	.5557895	1.6204
num						
Correct	.9549643	.2155266	-0.20	0.838	.6135909	1.486262
_cons	.1505126	.0531107	-5.37	0.000	.0753726	.3005608

Note: **_cons** estimates baseline odds.

```
  Thursday September 5 11:12:11 2019   Page 11

41 .
42 . //Health problems and BAME interaction//
43 . logit datt10 i.cond##i.ethnicity i.cage i.female i.idep i.paid i.num if comp==1, or

Iteration 0:   log likelihood = -302.59055
Iteration 1:   log likelihood = -294.25034
Iteration 2:   log likelihood = -292.74312
Iteration 3:   log likelihood = -290.23031
Iteration 4:   log likelihood = -289.55165
Iteration 5:   log likelihood = -289.50732
Iteration 6:   log likelihood = -289.50731

Logistic regression                             Number of obs     =        954
                                                LR chi2(12)       =      26.17
                                                Prob > chi2       =     0.0102
Log likelihood = -289.50731                     Pseudo R2         =     0.0432
```

datt10	Odds Ratio	Std. Err.	z	P>|z|	[95% Conf.	Interval]
cond						
Capsule	.6875368	.1819878	-1.42	0.157	.4092482	1.155061
CT colonography	.5434485	.158273	-2.09	0.036	.3070831	.9617471
ethnicity						
BAME	2.668504	3.201338	0.82	0.413	.2541573	28.01774
cond#ethnicity						
Capsule#BAME	2.369605	3.631279	0.56	0.573	.1175603	47.76299
CT colonography#BAME	5.661841	9.042489	1.09	0.278	.2474625	129.5406
cage						
65-69	.9066002	.2523885	-0.35	0.725	.5253512	1.564523
70-74	.7970324	.2438844	-0.74	0.458	.4375385	1.451896
female						
Yes	.5380308	.1284885	-2.60	0.009	.3369222	.859181
idep						
1 marker	1.332997	.3621348	1.06	0.290	.782679	2.270256
2-3 markers	2.5314	.8957882	2.62	0.009	1.26516	5.06496
paid						
Yes	.993958	.2734556	-0.02	0.982	.5796786	1.704311
num						
Correct	.962673	.2180531	-0.17	0.867	.6175538	1.500662
_cons	.1727472	.0568114	-5.34	0.000	.0906727	.3291136

```
Note: _cons estimates baseline odds.

44 .
45 . //Health problems and deprivation interaction//
46 . logit datt10 i.cond##i.dep i.ethnicity i.cage i.female i.paid i.num if comp==1, or

note: 3.cond#3.depr != 0 predicts failure perfectly
      3.cond#3.depr dropped and 1 obs not used

note: 1.cond#3.depr identifies no observations in the sample
note: 2.cond#3.depr omitted because of collinearity
Iteration 0:   log likelihood = -302.48909
Iteration 1:   log likelihood = -294.18407
Iteration 2:   log likelihood = -289.98635
Iteration 3:   log likelihood = -289.83164
Iteration 4:   log likelihood = -289.83128
Iteration 5:   log likelihood = -289.83128

Logistic regression                             Number of obs     =        953
                                                LR chi2(15)       =      25.32
                                                Prob > chi2       =     0.0459
Log likelihood = -289.83128                     Pseudo R2         =     0.0418
```

Thursday September 5 11:12:11 2019 Page 12

datt10	Odds Ratio	Std. Err.	z	P>|z|	[95% Conf.	Interval]
cond						
Capsule	.7088625	.2289895	-1.07	0.287	.3763497	1.335157
CT colonography	.5338427	.1891195	-1.77	0.076	.2666036	1.068958
depr						
1	1.375126	.612255	0.72	0.474	.5745892	3.290999
2	2.160367	1.200865	1.39	0.166	.7267371	6.422109
3	2.412483	2.730994	0.78	0.437	.2623504	22.18435
cond#depr						
Colonoscopy#3	1	(empty)				
Capsule#1	.8794388	.5557166	-0.20	0.839	.2548779	3.034444
Capsule#2	1.193254	1.049022	0.20	0.841	.2130191	6.684163
Capsule#3	1	(omitted)				
CT colonography#1	1.116122	.7644453	0.16	0.873	.2915502	4.272773
CT colonography#2	1.624588	1.464046	0.54	0.590	.2777523	9.502306
CT colonography#3	1	(empty)				
ethnicity						
BAME	6.434172	3.838078	3.12	0.002	1.998658	20.71319
cage						
65-69	.936774	.2627153	-0.23	0.816	.5406523	1.623124
70-74	.8234025	.2533449	-0.63	0.528	.4505199	1.504909
female						
Yes	.53614	.128135	-2.61	0.009	.3356178	.8564686
paid						
Yes	.9559571	.2622917	-0.16	0.870	.5583276	1.63677
num						
Correct	.9552938	.2165329	-0.20	0.840	.61263	1.489621
_cons	.1700755	.0577478	-5.22	0.000	.0874225	.3308722

Note: **_cons** estimates baseline odds.

```
47 .
48 . log close
      name:  <unnamed>
       log:  S:\FPHS_BSH_CancerComm\QQ PROJECTS 2012-17\BOWEL SCREENING PROJECT\Alternatives to
  log type:  smcl
 closed on:   5 Sep 2019, 11:08:22
```

For peer review only

Preferences for different diagnostic modalities to follow up abnormal colorectal cancer screening results: a hypothetical vignette study

Journal:	*BMJ Open*
Manuscript ID	bmjopen-2019-035264.R2
Article Type:	Original research
Date Submitted by the Author:	23-Apr-2020
Complete List of Authors:	Kaushal, Aradhna; University College London, Behavioural Science and Health<br>Stoffel , Sandro; University College London, Business School; University of Aberdeen<br>Kerrison, Robert; University College London, Epidemiology and Public Health<br>von Wagner, Christian; UCL, Department of Epidemiology and Public Health
**Primary Subject Heading**:	Public health
Secondary Subject Heading:	Communication, Oncology, Public health
Keywords:	PREVENTIVE MEDICINE, PUBLIC HEALTH, Endoscopy < GASTROENTEROLOGY

I, the Submitting Author has the right to grant and does grant on behalf of all authors of the Work (as defined in the below author licence), an exclusive licence and/or a non-exclusive licence for contributions from authors who are: i) UK Crown employees; ii) where BMJ has agreed a CC-BY licence shall apply, and/or iii) in accordance with the terms applicable for US Federal Government officers or employees acting as part of their official duties; on a worldwide, perpetual, irrevocable, royalty-free basis to BMJ Publishing Group Ltd ("BMJ") its licensees and where the relevant Journal is co-owned by BMJ to the co-owners of the Journal, to publish the Work in this journal and any other BMJ products and to exploit all rights, as set out in our licence.

The Submitting Author accepts and understands that any supply made under these terms is made by BMJ to the Submitting Author unless you are acting as an employee on behalf of your employer or a postgraduate student of an affiliated institution which is paying any applicable article publishing charge ("APC") for Open Access articles. Where the Submitting Author wishes to make the Work available on an Open Access basis (and intends to pay the relevant APC), the terms of reuse of such Open Access shall be governed by a Creative Commons licence – details of these licences and which Creative Commons licence will apply to this Work are set out in our licence referred to above.

Other than as permitted in any relevant BMJ Author's Self Archiving Policies, I confirm this Work has not been accepted for publication elsewhere, is not being considered for publication elsewhere and does not duplicate material already published. I confirm all authors consent to publication of this Work and authorise the granting of this licence.

For peer review only

Preferences for different diagnostic modalities to follow up abnormal colorectal cancer screening results: a hypothetical vignette study

Aradhna Kaushal[1], Sandro T. Stoffel[1,2], Robert Kerrison[1], and Christian von Wagner[1]

[1] Research Department of Behavioural Science and Health, University College London

[2] Business School, University of Aberdeen

Corresponding author:

Christian von Wagner, Research Department of Behavioural Science and Health, University College London, 1-19 Torrington Place, London, WC1E 7HB

Email: c.wagner@ucl.ac.uk

Word Count: 3160/3000

Tables and Figures: 5/5

6/10/2020

For peer review only - http://bmjopen.bmj.com/site/about/guidelines.xhtml

Abstract

Objectives

In England, a significant proportion of people who take part in the national bowel cancer screening programme (BCSP) and have a positive faecal occult blood test (FOBt) result, do not attend follow-up colonoscopy (CC). The aim of this study was to investigate differences in intended participation in a follow-up investigation by diagnostic modality offered including (CC), computed tomography colonography (CTC), or capsule endoscopy (CE).

Setting

We performed a randomised online experiment with individuals who had previously completed a FOBt as part of the English BCSP.

Methods

Participants (n=953) were randomly allocated to receive one of three online vignettes asking participants to imagine they had received an abnormal FOBt result, and that they had been invited for a follow-up test. The follow-up test offered was either: CC (n=346), CTC (n=302), or CE (n=305). Participants were then asked how likely they were to have their allocated test or if they refused either of the other tests. Respondents were also asked to cite possible emotional and practical barriers to follow up testing. Multi-variable logistic regression models were used to investigate intentions.

Results

Intention to have the test was higher in the CTC group (96.7%) compared with the CC group (91.8%; Odds Ratio [OR]: 2.64; 95% Confidence Intervals [CI]: 1.22-5.73). CTC was considered less "off-putting" (OR: 0.66, 95% CI: 0.46-0.94) and less uncomfortable

For peer review only - http://bmjopen.bmj.com/site/about/guidelines.xhtml

compared to CC (OR: 0.51, 95%; CI: 0.34-0.77). For those who did not intend to have the test they were offered, CE (39.7%) or no investigation (34.5%) was preferable to CC (8.6%) or CTC (17.2%).

Conclusions

Alternative tests have the potential to increase attendance at diagnostic follow-up appointments.

Word count: 267/250

Strengths and Limitations

- Use of a vignette design allowed us to investigate attitudes towards hypothetical scenarios which would be more difficult to carry out in a real-world setting.
- The use of comprehension checks after presenting vignettes ensure that participants engage with materials and had an understanding of the tests they were presented.
- The study design presents a hypothetical scenario and so it is not possible to say if intentions would align with behaviours in real-life.
- We only provided brief descriptions of the test which may have biased intentions.
- The online panel used in the survey has a very small number of BAME participants and therefore may not be representative of the UK population.

For peer review only

For peer review only - http://bmjopen.bmj.com/site/about/guidelines.xhtml

Introduction

Colorectal cancer (CRC) is the 4th most common cancer in the United Kingdom (UK), accounting for 12% of all cancer diagnosis, and is the second leading cause of cancer death. [1, 2] Screening can reduce mortality and improve survival by detecting CRC at an earlier stage, when treatment is more likely to be successful. [3] In England, the national bowel cancer screening programme (BCSP) sends a screening test to all men and women aged 60 to 74 to be completed at home. The test is offered once every two years, and can detect small amounts of blood in the stool, which may be indicative of CRC. This test until recently was the 'faecal occult blood test' (FOBt) and was replaced with the more sensitive 'feacal immunochemical test' (FIT) in June 2019. Around 2% of people who complete a FOBt have a positive test result and are invited for further investigation, usually a colonoscopy (CC). Despite being at increased risk, approximately 14% of those with an abnormal test result fail to complete their diagnostic follow up. [4-7] There are a number of reasons why a CC in a small proportion of these patients would not be appropriate, such as clinical decisions based on frailty, or having had a recent CC outside the BSCP. [8, 9]

Previous research point towards a range of psychological and practical factors which may influence decisions to have colorectal cancer screening. [9-11] Plumb et al (2017) reviewed the medical records of patients of 170 patients to identify patient factors associated with non-attendance at CC after a positive FOBt and found the most cited reason was "unwillingness to complete the test". [9] For some patients, this was due to anxiety about pain, and risks but in the majority of cases, the reason was unspecified. This is supported by a recent systematic review on incomplete diagnostic follow-up after an abnormal CRC screening result which identified perception of pain as the most commonly cited reason in addition to 'embarrassment' and 'being too busy'. [12] Consequently, for a vast majority of this group of

For peer review only - http://bmjopen.bmj.com/site/about/guidelines.xhtml

patients there is a strong need to develop patient or system level interventions to remove modifiable barriers to investigating the cause of their FOB test result. [13]

Some of these barriers may be directly or indirectly related to the nature of CC. CC involves inserting a long flexible tube with a tiny camera on the end into the anus in order to examine the bowel. If polyps (small, potentially cancerous growths) are found, they are removed during the procedure. The test requires the patient to restrict their diet, take a strong laxative, and arrange for someone to take them home afterwards due to drowsiness caused by a sedative. After the procedure, most people will need to rest for the remainder of the day. [14]

Considering the practical and emotional barriers which may inhibit someone from accepting the offer of further investigation via CC, it has been suggested that offering alternative tests, such as CTC or capsule endoscopy, may be more acceptable to patients. [15-17] These tests differ in terms of preparation, time in hospital and level of perceived invasiveness. Both of these tests, as with CC, require the patient to restrict their diet and take a laxative. In CTC, a small tube is used to inflate the bowel after which x-rays are used to take images of the bowel after which most people are able to resume their daily activities. [18] In capsule endoscopy, the patient ingests a capsule containing a small camera which takes photos inside the bowel and transmits them wirelessly to a receiver worn by the patient. [19] Patients are able to carry on with their normal activity and the capsule is passed through the body after eight hours. For both of these tests, a CC is usually recommended to remove any polyps if they are found.

CTC and CE could be offered individuals who decline to have a CC after a positive FOBt, in the expectation that providing individuals with alternative choices increases their feeling of autonomy and intrinsic motivation. [20-22] While offering choice seems promising, there exists no evidence that offering more than one screening test influences adherence and patient satisfaction. [23] Although CTC and CE are not currently endorsed as a screening methods, it is

For peer review only - http://bmjopen.bmj.com/site/about/guidelines.xhtml

possible that offering alternative tests for those who do stop engaging with the screening programme may increase intentions to have further investigations.

The present study aimed to evaluate whether offering different screening tests, such as CTC, or CE, increases intentions to attend for further investigation, when compared to offering a CC.

Methods

Design

We performed a randomised online experiment with a survey company called: 'Dynata' (formerly Survey Sampling International and Research Now). Potentially eligible men and women were invited to participate in the online experiment by Dynata, who invited them from their panel if they were: 1) aged 60-74 years and 2) lived in England. Potentially eligible men and women were informed that they would be rewarded with 'Dynata points', which they could exchange for money, or a donation to a charity, if they complete the survey.

Individuals who agreed to participate in the survey were asked about their screening history, which was used to filter ineligible adults. Only individuals who had completed a FOBt previously, and received a normal result, were eligible to participate in the survey. We excluded participants who had previously had a CC, a previous diagnosis of colorectal cancer or had parts of their bowel removed. Once eligibility to participate had been established using these criteria, participants were then randomly allocated to one of three experimental conditions in which they were asked to read a hypothetical vignette (see Supplementary Appendix 1).

Depending on the condition individuals were allocated to, the vignette asked them to imagine that their next FOBt result was abnormal, and they were being invited for a follow-up test

For peer review only - http://bmjopen.bmj.com/site/about/guidelines.xhtml

consisting of either CC, a CTC, or a CE. Each vignette included a some images of the test, a short description about what happens before, during and after the test, and any risks associated with the offered test. After reading the information, participants were required to successfully answer a 'comprehension check question' about the test (what happens during the test, how long it takes, and what happens afterwards), to ensure they had understood the information. If answered incorrectly, the information about the test was presented again and the participant is provided with another opportunity to answer the question. Once the participant answered the comprehension check question correctly, they were asked about their intention to have the follow-up test. Details of the comprehension check questions can be found in the Supplementary Appendix 1.

Measures

Intention to have follow-up test: On answering the comprehension check question correctly, participants were asked to indicate their intention to book an appointment (*"Considering all the information presented above, would you take up the offer of this test?"*), using a four-point Likert scale, with response options: '*definitely not*', '*probably not*', '*probably, yes*' and '*definitely, yes*'. [24, 25] These responses were dichotomised ('*probably, yes*' or '*definitely, yes*' vs. '*probably not*' or '*definitely not*') due to the relative lack of 'probably not' and 'definitely not' responses (see Supplementary Appendix 2).

Barriers for follow-up test: After indicating their intentions, participants were then asked to indicate how they felt about potential barriers for not wanting the tests ("*Please read each statement and select how strongly you agree or disagree with it?*"), using a four-point Likert scale, with response options: *'strongly agree', 'slightly agree', 'slightly disagree'* and *'strongly disagree'*. The barriers featured six emotional and four practical items, and were derived from previous surveys. [9, 26] The six emotional barrier items were: "*The preparation*

For peer review only - http://bmjopen.bmj.com/site/about/guidelines.xhtml

for the test (restricted diet and strong laxative) puts me off", "The test looks like it would be uncomfortable", "I would be embarrassed about taking the test", "I would worry about the risks associated with the test", "I would be afraid of getting an abnormal result" and *"Doing the test would make me worry more about bowel cancer".* The four practical barrier items were: *"I would not have time to do the test", "I have other problems to worry about", "It would be difficult to arrange transport to the hospital"* and *"I have other health problems that are more important"*. These responses were dichotomised ('*strongly agree'* or *'slightly agree'* vs '*slightly disagree'* or *'strongly disagree'*) due to the relative lack of 'strongly agree' responses (see Supplementary Appendix 2).

Preference of disinclined study participants. Study participants who indicated that they would not (probably or definitely) have the offered follow-up test were presented with information about the other two tests and asked which of the three tests they would prefer ("*Which of the two tests would you prefer to have*?"), with response options *"colonoscopy", "capsule", "CT colonography"* and *"none of them"*.

Numeracy skills. Numeracy skills were assessed by the question: 'Which of the following numbers represents the biggest risk of getting a disease?', with answer options '1/10', '1/100', '1/1000' and 'I don't know'. This measure was adapted from Lipkus and colleagues, who previously validated the question as a measure for numeracy skills.[15]

Socio-demographic variables. Details of the participant's age, gender, ethnicity, employment status, education, car and home ownership were collected at the end of the survey. Ethnicity data were recoded as 'White' (White British or Other White Background) and "BAME" (Black, Asian and minority ethnic) due to the small numbers in each group. The three variables on education, car and home ownership were used to calculate a proxy measure for socio-economic deprivation. One point was given to a participant if their

For peer review only - http://bmjopen.bmj.com/site/about/guidelines.xhtml

household did not own a car or van, if they had no formal qualifications and if they did not own their own home. [27-29] Scores, therefore, ranged from 0 to 3, with high scores indicating higher levels of deprivation.

Sample size calculation

Sample size of this study was calculated prior to data collection based on the results of a soft launch. We calculated that we needed approximately 300 participants per condition to detect differences of at least 5 percentage points in proportion of intenders effect size between any of the three conditions, with a power of 80% and an alpha value of 0.05. [30]

Statistical analysis

Our primary outcome was intention to have the offered follow-up test after exposure to the allocated vignette. Our secondary outcomes were the responses to the perceived emotional and practical barrier questions. Descriptive statistics were used to report the socio-demographic characteristics of the study population. Uni-variable and multi-variable logistic regression models were used to investigate the effect of offering alternative follow-up tests on participants' intentions to have further investigations, as well as their perceived emotional and practical barriers of the test offered. Covariates that were included in the regression models were age, gender, ethnicity, deprivation score, employment status and numeracy. All statistical analysis was conducted using Stata/SE (version 15.1). [31] The survey, data and Stata codes for the experiment are available via Open Science Framework: https://osf.io/fx69t/?view_only=46ba2185c26940a89c19bde28da40a9c.

For peer review only - http://bmjopen.bmj.com/site/about/guidelines.xhtml

Results

Participants

In total, 1,926 adults responded to the online invitation. Of these, 481 (25.0%) were excluded due to their age or medical history (i.e. previous bowel cancer diagnosis or removal of part of the bowel). A further 372 (19.3%) were excluded as they had never been invited for, or completed, a FOBt, and an additional 46 (2.4%) were excluded as they had previously received an abnormal FOBt result. Of the remaining 1,027 adults who were eligible for inclusion, 953 (92.8%) completed the survey (see Figure 1 for an overview of participants through the study).

- Figure 1 here -

Table 1 shows the participant characteristics by study condition. Around half of participants were male (54.5%), of a White ethnic background (98.6%), not in paid employment (75.1%), had a formal education (62.7%), owned car (87.1%), and owned a house (87.4%).

For peer review only - http://bmjopen.bmj.com/site/about/guidelines.xhtml

For peer review only

Table 1. Socio-demographic characteristics of the study population (N=953)

	Colonoscopy condition (N=305)		Capsule endoscopy condition (N=346)		CT Colonography condition (N=302)		Overall (N=953)	
**Age**								
60-64	98	(32.1%)	94	(27.2%)	109	(36.1%)	301	(31.6%)
65-69	123	(40.3%)	125	(36.1%)	109	(36.1%)	357	(37.5%)
70-74	84	(27.5%)	127	(36.7%)	84	(27.8%)	295	(31.0%)
**Gender**								
Male	151	(49.5%)	201	(58.1%)	167	(55.3%)	519	(54.5%)
Female	154	(50.5%)	145	(41.9%)	135	(44.7%)	434	(45.5%)
**Ethnicity**								
White	301	(98.7%)	341	(98.6%)	298	(98.7%)	940	(98.6%)
BMAE	4	(1.3%)	5	(1.4%)	4	(1.3%)	13	(1.4%)
**Paid employment**								
No	221	(72.5%)	260	(75.1%)	235	(77.8%)	716	(75.1%)
Yes	84	(27.5%)	86	(24.9%)	67	(22.2%)	237	(24.9%)
**Education**								
No A levels	119	(39.0%)	125	(36.1%)	112	(37.4%)	356	(37.4%)
A levels or higher	186	(61.0%)	221	(63.9%)	190	(62.6%)	597	(62.6%)
**Car ownership**								
No	36	(11.8%)	46	(13.3%)	41	(13.6%)	123	(12.9%)
Yes	269	(88.2%)	300	(86.7%)	261	(86.4%)	830	(87.1%)
**House ownership**								
No	44	(14.4%)	46	(13.3%)	30	(9.9%)	120	(12.6%)
Yes	261	(85.6%)	300	(86.7%)	272	(90.1%)	833	(87.4%)
**Individual social deprivation**								
0 markers	223	(73.1%)	242	(69.9%)	221	(73.2%)	686	(72.0%)
1 marker	58	(19.0%)	80	(23.1%)	63	(20.8%)	201	(21.1%)
2-3 markers	24	(7.9%)	24	(6.9%)	18	(5.9%)	66	(6.9%)
**Numeracy question**								
Wrong	163	(53.4%)	180	(52.0%)	151	(50.0%)	494	(51.8%)
Correct	142	(46.6%)	166	(48.0%)	151	(50.0%)	459	(48.2%)

*p-value refers to Chi-Square test of independence if not stated differently

The three demographic questions on education, car and house ownership were used to calculate a proxy measure for socio-economic deprivation. One point was given to an individual if their household did not have a car or van, if they had no formal qualifications and if they did not own their own home [1, 2]. Scores, therefore, ranged from 0 to 3, with high scores indicating higher levels of social deprivation. Due to low frequencies, we collapsed the two groups that either had a score of 2 or 3 into one group called 2-3 markers.

For peer review only

For peer review only - http://bmjopen.bmj.com/site/about/guidelines.xhtml

Intention to have the allocated test

A large majority (93.7%) intended to have the allocated test, although this was graded by ethnicity (61.5% vs 94.4%) and deprivation (2-3 markers: 87.9% vs. 0 markers: 95.5%). The proportion of intenders was highest among those who were offered CTC, followed by those who were offered capsule, and finally those who were offered CC (96.7% vs. 93.4% vs. 91.8% respectively, $\chi 2$=(2, N=953) =6.64, p=0.036).

In the multi-variable model, participants remained more likely to accept further investigation if the offered test was CTC compared to CC, after adjusting for co-variates (Table 2; OR: 2.64, 95% CI: 1.22–5.73, p=0.014). Offering CE did not significantly increase intentions compared to CC (OR: 1.28, 95% CI: 0.69–2.35, p=0.432). The multi-variable analysis also revealed that intention to undergo follow-up tests was lower among BAME groups than White ethnic groups (OR: 0.09, 95% CI: 0.03–0.29, p<0.001), as well as more deprived individuals (1 marker: OR: 0.45, 95% CI: 0.25–0.84, p=0.012; 2-3 markers: OR: 0.34, 95% CI: 0.15–0.79, p=0.012).

Preferences of disinclined study participants

In total, 58 (6.1%) study participants did not intend to have their offered follow-up test. Independently from the initial offer, most disinclined study participants would either choose CE (N=23, 39.7%) or none of the tests (N=20, 34.5%). A Fisher's exact test demonstrated that preferences were not influenced by the initial random allocation (p=0.806).

For peer review only - http://bmjopen.bmj.com/site/about/guidelines.xhtml

Table 2. Association between test offered and wanting to have the test (N=953)

	Unadjusted model		Adjusted model	
	OR	95% CI	OR	95% CI
**Condition**				
Colonoscopy	Ref.		Ref.	
Capsule endoscopy	1.25	0.70 - 2.26	1.28	0.69 - 2.35
CT colonography	2.61	**1.23 - 5.53***	2.64	**1.22 - 5.73***
**Age**				
60-64			Ref.	
65-69			0.70	0.36 - 1.37
70-74			1.42	0.63 - 3.22
**Gender**				
Male			Ref.	
Female			1.09	0.62 - 1.91
**Ethnicity**				
White			Ref.	
BAME			0.09	**0.03 - 0.29****
**Deprivation**				
0 makers			Ref.	
1 marker			0.45	**0.25 - 0.84***
2-3 markers			0.34	**0.15 - 0.79***
**Paid employment**				
No			Ref.	
Yes			0.87	0.45 - 1.67
**Numeracy task**				
Wrong			Ref.	
Correct			1.58	0.89 - 2.78

Logistic regression models presented; OR: Odds ratio; CI: Confidence intervals; BAME: Black, Asian and Minority Ethnic; * $p<0.05$; ** $p<0.01$

Perceived emotional barriers

Those who were randomised to CTC were less likely to agree that the test is off-putting (OR: 0.66, 95% CI: 0.46–0.94, p=0.022) or uncomfortable (OR: 0.51, 95% CI: 0.34–0.77, p=0.001) than those who were offered a CC. Participants who were offered CE as a follow-up test were less likely to perceive the test as uncomfortable (OR: 0.11: 95% CI: 0.07–0.16, p<0.001), embarrassing (OR: 0.33, 95% CI: 0. 23–0.48, p<0.001), or creating worries about the risks associated with the test (OR: 0.70, 95% CI: 0.51–0.97, p=0.031) than those who were offered a CC (Table 3).

For peer review only

For peer review only - http://bmjopen.bmj.com/site/about/guidelines.xhtml

The table also shows that women were more likely to perceive the offered tests as off-putting (OR: 1.41, 95% CI: 1.06–1.88, p=0.018), embarrassing (OR: 1.83, 95% CI: 1.36–2.47, p<0.001) and causing worries about the risks (OR: 1.46, 95% CI: 1.11–1.91, p=0.007) and results of the test (OR: 1.57, 95% CI: 1.20–2.05, p=0.001).

Higher deprivation was associated with worrying about the risks associated with the test (OR: 1.50, 95% CI: 1.08-2.08, p=0.014) and numeracy was positively associated with being afraid of the results (OR: 1.32, 95% CI: 1.01-1.72, p=0.39). Sub-group analysis did not reveal any significant interactions between these demographic variables and test allocation (Supplementary Appendix 3).

For peer review only - http://bmjopen.bmj.com/site/about/guidelines.xhtml

Table 3. Association between test offered and emotional barrier items (N=953).

	Off-putting		Uncomfortable		Embarrassing		Worry about risks of test		Afraid of results		Worry about cancer	
	OR	95% CI	OR	95% CI	OR	95% CI	OR	95% CI	OR	95% CI	OR	95% CI
**Condition**												
Colonoscopy	Ref.		Ref.		Ref.		Ref.		Ref.		Ref.	
Capsule endoscopy	0.75	0.53 - 1.047	0.11	**0.07 - 0.16****	0.33	**0.23 - 0.48****	0.70	**0.51 - 0.97***	0.75	0.54 - 1.03	0.82	0.59 - 1.13
CT colonography	0.66	**0.47 - 0.94***	0.51	**0.34 - 0.77****	0.72	0.51 - 1.02	0.73	0.52 - 1.02	0.92	0.66 - 1.28	1.15	0.83 - 1.60
**Age**												
60-64	Ref.		Ref.		Ref.		Ref.		Ref.		Ref.	
65-69	0.99	0.69 - 1.41	0.91	0.62 - 1.32	0.81	0.56 - 1.16	0.85	0.61 - 1.19	0.68	**0.48 - 0.94***	0.67	**0.48 - 0.93***
70-74	1.05	0.72 - 1.54	0.89	0.60 - 1.33	0.88	0.59 - 1.31	0.88	0.62 - 1.27	0.71	0.49 - 1.01	0.74	0.52 - 1.06
**Gender**												
Male	Ref.		Ref.		Ref.		Ref.		Ref.		Ref.	
Female	1.41	**1.06 - 1.88***	1.03	0.76 - 1.39	1.83	**1.36 - 2.47****	1.46	**1.11 - 1.91****	1.57	**1.20 - 2.05****	1.05	0.801 - 1.38
**Ethnicity**												
White	Ref.		Ref.		Ref.		Ref.		Ref.		Ref.	
BAME	2.22	0.73 - 6.75	1.22	0.33 - 4.55	1.19	0.34 - 4.19	2.02	0.66 - 6.17	3.97	0.86 - 18.33	1.76	0.58 - 5.34
**Deprivation**												
0 markers	Ref.		Ref.		Ref.		Ref.		Ref.		Ref.	
1 marker	1.06	0.75 - 1.50	1.36	0.93 - 1.97	0.99	0.69 - 1.43	1.50	**1.09 - 2.08***	1.36	0.97 - 1.89	1.34	0.97 - 1.86
2-3 markers	1.43	0.84 - 2.44	0.65	0.36 - 1.15	1.29	0.73 - 2.26	1.26	0.75 - 2.14	0.68	0.41 - 1.14	0.99	0.58 - 1.680
**Paid employment**												
No	Ref.		Ref.		Ref.		Ref.		Ref.		Ref.	
Yes	0.99	0.70 - 1.40	1.45	1.00 - 2.11	1.41	1.00 - 2.01	1.11	0.80 - 1.53	1.19	0.86 - 1.65	1.01	0.73 - 1.40
**Numeracy task**												
Wrong	Ref.		Ref.		Ref.		Ref.		Ref.		Ref.	
Correct	1.03	0.79 - 1.37	0.88	0.66 - 1.19	1.13	0.84 - 1.52	1.08	0.83 - 1.42	1.32	**1.01 - 1.72***	1.22	0.94 - 1.59

Logistic regression models presented; OR: Odds ratio; CI: Confidence intervals; BAME: Black, Asian and Minority Ethnic; * $p<0.05$; ** $p<0.01$

For peer review only

For peer review only - http://bmjopen.bmj.com/site/about/guidelines.xhtml

Perceived practical barriers

Study participants who were offered CE, instead of CC, were less likely to think that they wouldn't have the time to do the test (OR: 0.27, 95% CI: 0.10–0.70, p=0.007). There were no other differences in practical barriers by test offered (See table 4). Health problems were cited as a barrier by participants with a higher number of deprivation markers (OR: 2.54, 95% CI: 1.27-5.07 , p=0.009), those with a BAME background (OR: 6.45, 95% CI:2.01-20.73 , p=0.002) and by men (OR: 1.85, 95% CI: 2.94-1.16, p=0.009). Those from BAME groups (OR:9.37, 95% CI:2.23-39.36, p=0.002), and those who were in paid in employment (OR:2.42, 95% CI:1.09-5.38 , p=0.03) were more likely to report not having enough time as a barrier to a follow-up investigation. Participants with a higher number of deprivation markers were also more likely to cite difficulties with transport (OR: 3.52 , 95% CI:1.96-6.31, p<0.001) as a barrier to having a follow-up investigation. Sub-group analysis did not reveal any significant interactions between these demographic variables and test allocation (Supplementary Appendix 3) .

For peer review only - http://bmjopen.bmj.com/site/about/guidelines.xhtml

Table 4. Association between study condition and practical barrier items (n=953).

	No time		Other problems		Difficulties with transport		Health problems	
	OR	95% CI	OR	95% CI	OR	95% CI	OR	95% CI
**Condition**								
Colonoscopy	Ref.		Ref.		Ref.		Ref.	
Capsule endoscopy	0.27	**0.10 - .70****	0.75	0.52 - 1.09	0.76	0.48 - 1.18	0.70	0.42 - 1.17
CT colonography	0.46	0.19 - 1.09	0.80	0.55 - 1.18	0.64	0.40 - 1.04	0.58	0.33 - 1.01
**Age**								
60-64	Ref.		Ref.		Ref.		Ref.	
65-69	1.06	0.43 - 2.63	0.91	0.62 - 1.35	0.77	0.49 - 1.23	0.92	0.53 - 1.58
70-74	1.28	0.48 - 3.39	1.03	0.68 - 1.55	0.65	0.39 - 1.10	0.80	0.44 - 1.46
**Gender**								
Male	Ref.		Ref.		Ref.		Ref.	
Female	0.73	0.35 - 1.55	1.18	0.86 - 1.61	1.33	0.91 - 1.96	0.54	**0.34 - 0.86****
**Ethnicity**								
White	Ref.		Ref.		Ref.		Ref.	
BAME	9.37	**2.23 - 39.36****	2.28	0.73 - 7.10	3.32	0.99 - 11.13	6.45	**2.01 - 20.73****
**Deprivation**								
0 markers	Ref.		Ref.		Ref.		Ref.	
1 marker	1.40	0.59 - 3.32	1.11	0.76 - 1.61	1.65	**1.06 - 2.58***	1.35	0.79 - 2.29
2-3 markers	2.41	0.78 - 7.49	1.37	0.77 - 2.44	3.52	**1.96 - 6.31****	2.54	**1.27 - 5.07****
**Paid employment**								
No	Ref.		Ref.		Ref.		Ref.	
Yes	2.42	**1.09 - 5.38***	0.97	0.66 - 1.42	0.88	0.55 - 1.41	0.95	0.56 - 1.63
**Numeracy task**								
Wrong	Ref.		Ref.		Ref.		Ref.	
Correct	0.60	0.28 - 1.26	0.83	0.61 - 1.14	0.74	0.50 - 1.09	0.96	0.61 - 1.49

Logistic regression models presented; OR: Odds ratio; CI: Confidence intervals; BAME: Black, Asian and Minority Ethnic; * $p<0.05$; ** $p<0.01$

For peer review only

For peer review only - http://bmjopen.bmj.com/site/about/guidelines.xhtml

Discussion

This study investigated whether offering alternative follow-up tests increases intention to undergo further investigation in the context of CRC screening. The results of our study show that, while the vast majority of individuals would accept any test, offering CTC instead of CC may increase acceptance of follow-up tests. Findings from our study suggest that this is due to individuals perceiving CTC as less 'off-putting' and 'uncomfortable' than CC. No differences were found in intention between those randomised to CE compared to CC despite CE appearing less uncomfortable, less embarrassing, causing less worry about the risks associated with the test, and taking test time. However, for the small number of participants who did not intend to take the offered test, CE appeared to be the preferred test when presented with all options.

The findings from this study are supported by two trials: one in in France, by Pioche et al. (2018) and one in Italy, by Sali et al. (2013). [32, 33] Both studies randomised patients with abnormal FOBt results who had refused the offer of a colonoscopy to be invited for another test. Pioche and colleagues found that CTC was more appealing than CE (7.4% vs. 5.0% having the investigation) with no differences found in clinical outcomes. The authors concluded that those who refuse CC are difficult to recruit into the screening programme and that simply offering a different test is not effective. Sali and colleagues also found that invitation to CTC was more preferable, but in comparison to CC (35.7% vs. 14.1% attending). These trials were carried out in France and Italy and may not be generalisable to a UK population and the NHS screening programme. Importantly, as the studies focused on patients who had already refused CC it looked at preferences in a pre-selected sample. This is reflected in the small sample sizes in both studies with only 50-100 participants in each

For peer review only - http://bmjopen.bmj.com/site/about/guidelines.xhtml

comparison group, making it difficult to draw conclusions about the population-level benefits of offering alternative tests.

Our study also observed a lower likelihood of ethnic minorities to accept any follow-up test. This is supported by a recent systematic review of characteristics of incomplete diagnostic follow-up after a positive bowel screening test identified non-White participants as less likely to attend in 9 out of 10 studies. [12] Analysis of barriers suggest that other health problems and not having enough time may be additional contributing factors to intention. Previous research has identified embarrassment as a potential barrier to colorectal cancer screening, although this was not found in this study. [34] We also found that not having enough time, and difficulties arranging transport are barriers to screening for people in employment, those with a higher level of deprivation and those from a BAME background. This is consistent with evidence from an American study, which showed that the location of the CC appointment and the availability of evening and weekend appointments are important factors limiting uptake. [35] These findings should be interpreted with care due to the very small number of BAME participants in the entire sample and the heterogeneity of the group. Future research should aim to recruit a larger and more representative BAME sample so findings can be presented for each ethnic group.

While our study provides support that individuals perceive follow-up tests differently, we only offered one test at random. Future research should present all three tests to participants in a random order to analyse whether offering individuals the choice between different tests increases intentions. [23] This could also include investigation of other diagnostic tests such as stool DNA tests. [36]

For peer review only - http://bmjopen.bmj.com/site/about/guidelines.xhtml

Strengths and limitations

Our study has several limitations. Firstly, our study used hypothetical scenarios and non-representative online study samples. Although the vignettes were developed in collaboration with clinicians, it was not possible in the space available to fully explain the level of burden associated with each test which may have biased responses. For example, in the CE vignette we stated that after the procedure it was possible to return to normal daily activities, when in reality the patient may be required to take booster laxatives and will have to return another day to have the recording analysed. It is possible that CE emerged as the second most preferred test due to the relatively positive description of the procedure.

Furthermore, the experiments lacked behavioural validation, in that they only measured intentions and choice in a hypothetical setting. The next step would be to test the effect of offering alternative follow-up tests under more ecologically valid conditions in a randomised controlled trial.

A strength of our study is that we used comprehension checks to ensure that all study participants sufficiently engaged with the provided information about the follow-up test.

Conclusions

While the results from this randomised online experiment show a high acceptance of the current practice of the NHS Bowel Screening Programme to invite individuals who have an abnormal FOBt result for a follow-up CC, our study also provides some evidence that offering CTC, instead of CC, may increase the total number of participants undertaking a follow-up test. Although CTC and CE are not currently endorsed as a screening modalites, our study provides some evidence that for high-risk and hard-to-engage groups, such as those

For peer review only - http://bmjopen.bmj.com/site/about/guidelines.xhtml

who have a positive FOBt but refuse follow-up, offering alternative tests may increase intentions to have engage with medical professionals and receive appropriate care.

For peer review only

For peer review only - http://bmjopen.bmj.com/site/about/guidelines.xhtml

Abbreviations

CRC: colorectal cancer; FOBt: faecal occult blood test; CC: Colonoscopy; CTC: CT Colonography; CE: Capsule Endoscopy; OR: Odds Ratio; 95% CI: 95% Confidence Interval

Declarations

Ethics and consent to participate

This research project was approved by UCL Research ethics committee (approval number 14687/001). All participants were asked to give explicit written consent for their data to be used and published as part of this research project before taking part in this study.

Consent for publication

Not applicable.

Competing interests

The authors have declared that no competing interests exist.

Supporting information

Supporting file contains demographic characteristics of the study samples, additional description of results and all outcome measures of the experimental studies.

Patient and Public Involvement

The design of this study followed a direct question posed by representatives of Public Health England and was informed by previous research on this topic and close collaboration with experts involved in administering relevant tests (see acknowledgments).

Availability of data and materials

All data files and materials are publicly available via the Open Science Framework and can be accessed at https://osf.io/fx69t/?view_only=46ba2185c26940a89c19bde28da40a9c.

For peer review only

For peer review only - http://bmjopen.bmj.com/site/about/guidelines.xhtml

Funding

This report presents independent research commissioned and funded by the National Institute for Health Research (NIHR) Policy Research Programme, conducted through the Policy Research Unit in Cancer Awareness, Screening and Early Diagnosis, PR-PRU-1217-21601. The views expressed are those of the authors and not necessarily those of the NIHR, the Department of Health and Social Care or its arm's length bodies, or other Government Departments. RK is supported by a Cancer Research UK Population Research Fellowship [C68512/A28209].

Authors' contributions

AK, STS and CW developed the study concept. All authors contributed to the study design. AK, STS and CW performed the data analysis and interpretation. AK and STS drafted the manuscript, RK and CW provided critical revisions. All authors approved the final version of the manuscript for submission.

Acknowledgements

We would like to acknowledge the contribution to the study materials by Professor Owen Epstein, Tim Rayne and Dr. Andrew Plumb.

For peer review only

For peer review only - http://bmjopen.bmj.com/site/about/guidelines.xhtml

References

1. Cancer Research UK. Bowel cancer incidence statistics. 2018.

2. Cancer Research UK. Bowel cancer mortality statistics. 2018.

3. Logan RF, Patnick J, Nickerson C, Coleman L, Rutter MD and von Wagner C. Outcomes of the Bowel Cancer Screening Programme (BCSP) in England after the first 1 million tests. *Gut*. 2012; 61: 1439-46.

4. Hirst Y, Stoffel S, Baio G, McGregor L and von Wagner C. Uptake of the English Bowel (Colorectal) Cancer Screening Programme: an update 5 years after the full roll-out. *European Journal of Cancer*. 2018; 103: 267-73.

5. Kearns B, Whyte S, Seaman HE, et al. Factors associated with completion of bowel cancer screening and the potential effects of simplifying the screening test algorithm. 2016; 114: 327.

6. Beshara A, Ahoroni M, Comanester D, et al. Association Between Time to Colonoscopy After a Positive Guaiac Fecal Test Result and Risk of Colorectal Cancer and Advanced Stage Disease at Diagnosis. *International journal of cancer*. 2019.

7. Kaalby L, Rasmussen M, Zimmermann-Nielsen E, Buijs MM and Baatrup G. Time to colonoscopy, cancer probability, and precursor lesions in the Danish colorectal cancer screening program. *Clinical Epidemiology*. 2019; 11: 659.

8. Paynter S. A pilot study exploring primary care involvement in re-engaging patients with the Bowel Cancer Screening Programme who have tested positive on screening but failed to complete colonoscopy. Public Health England,, 2015.

9. Plumb AA, Ghanouni A, Rainbow S, et al. Patient factors associated with non-attendance at colonoscopy after a positive screening faecal occult blood test. *Journal of medical screening*. 2017; 24: 12-9.

10. Honein-AbouHaidar GN, Kastner M, Vuong V, et al. Systematic review and meta-study synthesis of qualitative studies evaluating facilitators and barriers to participation in colorectal cancer screening. 2016; 25: 907-17.

11. Power E, Miles A, Von Wagner C, Robb K and Wardle JJFO. Uptake of colorectal cancer screening: system, provider and individual factors and strategies to improve participation. 2009; 5: 1371-88.

12. Dalton AJJoPH. Incomplete diagnostic follow-up after a positive colorectal cancer screening test: a systematic review. 2017; 40: e46-e58.

13. Selby K, Baumgartner C, Levin TR, et al. Interventions to improve follow-up of positive results on fecal blood tests: a systematic review. *Annals of internal medicine*. 2017; 167: 565-75.

For peer review only - http://bmjopen.bmj.com/site/about/guidelines.xhtml

14. Carlson CM, Kirby KA, Casadei MA, Partin MR, Kistler CE and Walter LC. Lack of follow-up after fecal occult blood testing in older adults: inappropriate screening or failure to follow up? *Archives of internal medicine*. 2011; 171: 249-56.

15. Groth S, Krause H, Behrendt R, et al. Capsule colonoscopy increases uptake of colorectal cancer screening. *BMC gastroenterology*. 2012; 12: 80.

16. Rex DK and Lieberman DA. A survey of potential adherence to capsule colonoscopy in patients who have accepted or declined conventional colonoscopy. *Journal of clinical gastroenterology*. 2012; 46: 691-5.

17. Pooler BD, Baumel MJ, Cash BD, et al. Screening CT colonography: multicenter survey of patient experience, preference, and potential impact on adherence. *American journal of roentgenology*. 2012; 198: 1361-6.

18. Kim DH, Pickhardt PJ, Taylor AJ, et al. CT colonography versus colonoscopy for the detection of advanced neoplasia. *New England journal of medicine*. 2007; 357: 1403-12.

19. Iddan G, Meron G, Glukhovsky A and Swain P. Wireless capsule endoscopy. *Nature*. 2000; 405: 417.

20. Katz I and Assor A. When choice motivates and when it does not. *Educational Psychology Review*. 2007; 19: 429.

21. Patall EA, Cooper H and Robinson JC. The effects of choice on intrinsic motivation and related outcomes: a meta-analysis of research findings. *Psychological bulletin*. 2008; 134: 270.

22. Zuckerman M, Porac J, Lathin D and Deci EL. On the importance of self-determination for intrinsically-motivated behavior. *Personality and social psychology bulletin*. 1978; 4: 443-6.

23. Partin MR, Powell AA, Burgess DJ and Wilt TJ. Bringing an organizational perspective to the optimal number of colorectal cancer screening options debate. *Journal of general internal medicine*. 2012; 27: 376-80.

24. Stoffel ST, Goodwin M, Sieverding M, Vlaev I and von Wagner C. Testing verbal quantifiers for social norms messages in cancer screening: evidence from an online experiment. *BMC public health*. 2019; 19: 658.

25. Stoffel ST, Hirst Y, Ghanouni A, et al. Testing active choice for screening practitioner's gender in endoscopy among disinclined women: An online experiment. *Journal of medical screening*. 2019; 26: 98-103.

26. Smith S, McGregor L, Raine R, Wardle J, Von Wagner C and Robb K. Inequalities in cancer screening participation: examining differences in perceived benefits and barriers. *Psycho-Oncology*. 2016; 25: 1168-74.

For peer review only

For peer review only - http://bmjopen.bmj.com/site/about/guidelines.xhtml

27. McGregor LM, von Wagner C, Vart G, et al. The impact of supplementary narrative-based information on colorectal cancer screening beliefs and intention. *BMC cancer*. 2015; 15: 162.

28. Von Wagner C, Bonello B, Stoffel S, et al. Barriers to bowel scope (flexible sigmoidoscopy) screening: a comparison of non-responders, active decliners and non-attenders. *BMC public health*. 2018; 18: 1161.

29. Wardle J, McCaffery K, Nadel M and Atkin W. Socioeconomic differences in cancer screening participation: comparing cognitive and psychosocial explanations. *Social science & medicine*. 2004; 59: 249-61.

30. Cohen J. *Statistical power analysis for the behavioral sciences*. Routledge, 2013.

31. StataCorp. Stata Statistical Software: Release 16. College Station, TX: StataCorp LLC, 2019.

32. Pioche M, Ganne C, Gincul R, et al. Colon capsule versus computed tomography colonography for colorectal cancer screening in patients with positive fecal occult blood test who refuse colonoscopy: a randomized trial. *Endoscopy*. 2018; 50: 761-9.

33. Sali L, Grazzini G, Ventura L, et al. Computed tomographic colonography in subjects with positive faecal occult blood test refusing optical colonoscopy. *Digestive and Liver Disease*. 2013; 45: 285-9.

34. Robb KA, Solarin I, Power E, Atkin W and Wardle J. Attitudes to colorectal cancer screening among ethnic minority groups in the UK. *BMC public health*. 2008; 8: 34.

35. O'Malley A, Beaton E, Yabroff K, Abramson R and Mandelblatt JJPm. Patient and provider barriers to colorectal cancer screening in the primary care safety-net. 2004; 39: 56-63.

36. Imperiale TF, Ransohoff DF, Itzkowitz SH, et al. Multitarget stool DNA testing for colorectal-cancer screening. *New England Journal of Medicine*. 2014; 370: 1287-97.

For peer review only

For peer review only - http://bmjopen.bmj.com/site/about/guidelines.xhtml

490 **Figure 1. Participant flow diagram**

For peer review only

For peer review only - http://bmjopen.bmj.com/site/about/guidelines.xhtml

Figure 1. Participant flow diagram

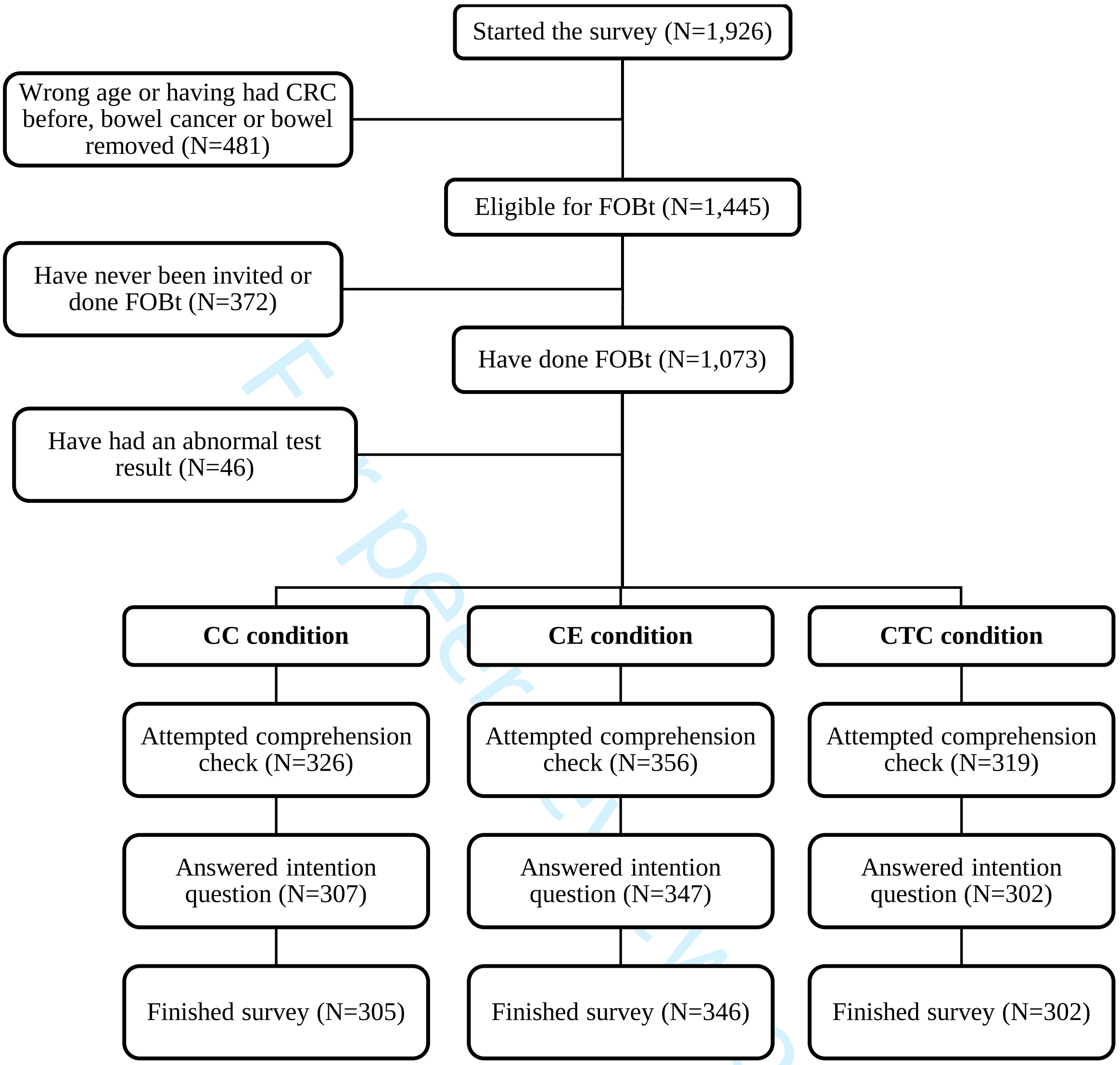

Investigating offers of alternative investigations after a positive bowel screening test

Welcome page

Thank you for agreeing to complete this survey.

It should take you no longer than 10 minutes to complete. You may, however, take as long as you want.

No identifiable information will be collected, meaning that your responses will be anonymous and it will not be possible for you to be identified as an individual. All information will be handled in accordance with the provisions of the Data Protection Act, 1998 and the General Data Protection Regulation (2018).

Please try to respond to **ALL** the questions – an incomplete questionnaire will make your responses less valid.

For peer review only

For peer review only - http://bmjopen.bmj.com/site/about/guidelines.xhtml

Information sheet

Title of Project: Investigating offers of alternative investigations after a positive bowel screening test

This study has been approved by the UCL Research Ethics Committee: Project ID number: 14687/001

We would like to invite you to take part in this study. You should only take part if you want to. Before you decide whether to take part, it is important for you to read the following information carefully and discuss it with others if you wish. Please ask us if anything is unclear or if you would like more information.

What is the purpose of the study?

This study is concerned with how individuals make choices related to bowel cancer screening. To explore this further, we have developed a survey to ask people about their preferences.

Why have I been invited?

We are inviting men and women aged between 60 and 74 years or older to take part in this survey.

Do I have to take part?

It is up to you to decide to join the study. If you decide to withdraw you will be asked what you wish to happen to the data you have provided up that point.

What will I have to do if I take part?

If you choose to take part we would ask you to complete an online survey which should take around 10 minutes to complete.

What are the possible disadvantages and risks of taking part?

This study carries very little risk for the participants. There are no right or wrong answers to the questions. As we are asking you about health issues that may be sensitive, there is a slight possibility that you will feel worried.

What are the possible benefits of taking part?

In addition to the remuneration you receive from SSI, this study will help us to better understand people's preferences for different bowel cancer screening tests.

What if there is a problem?

If you have a concern about any aspects of the study you should in the first instance contact Aradhna Kaushal (aradhna.kaushal.14@ucl.ac.uk) who will do her best to answer your questions. If you would like to discuss a problem with someone not directly involved in the project, you can contact the chairs of UCLs research ethics committees (ethics@ucl.ac.uk).

Will my taking part in this project be kept confidential?

Yes. All information which is collected about you during the course of the research is anonymous and not linked to any of your personal details. This means that you cannot be recognised from the information you provide. Our procedures for storage, processing, handling and destroying the information you give us are in line with Data Protection Act (1998) and the General Data Protection Regulation (2016). The survey will be conducted using SurveyMonkey which stores data collected on servers located in the United States.

For peer review only - http://bmjopen.bmj.com/site/about/guidelines.xhtml
For peer review only

What will happen to the results of the research project?

We hope to report the findings from the study in an academic journal. You will not be identified in any reports or publications from the study.

Who is organising and funding the research?

Dr Aradhna Kaushal in the Department of Behavioural Science and Health, Institute of Epidemiology and Healthcare, UCL. The project is funded by the Department of Health.

Contact for further information

Researcher: Dr Aradhna Kaushal | aradhna.kaushal.14@ucl.ac.uk | 020 7679 8254

Principal Researcher: Dr Christian von Wagner | c.wagner@ucl.ac.uk

UCL Data Protection Officer: Lee Shailer | l.shailer@ucl.ac.uk

Thank you for reading this information sheet and for considering to take part in this research study.

For peer review only

For peer review only - http://bmjopen.bmj.com/site/about/guidelines.xhtml

Consent form

Thank you for considering taking part in this research. If you have any questions arising from the Information explanation already given to you, please ask the researcher before you decide whether to join in.

I confirm that I understand that by ticking/initialling each box below I am consenting to this element of the study. I understand that it will be assumed that unticked/initialled boxes means that I DO NOT consent to that part of the study. I understand that by not giving consent for any one element that I may be deemed ineligible for the study.

	Tick Box
**1. I confirm that I have read and understood the information sheet for the study above. I have had an opportunity to consider the information and what will be expected of me. I have also had the opportunity to ask questions.**	
**2. I understand that my participation is voluntary and that I am free to withdraw at any time without giving a reason**	
**3. I understand that my data gathered in this study will be stored anonymously and securely. It will not be possible to identify me in any publications.**	
**4. I understand that the data will not be made available to any commercial organisations but is solely the responsibility of the researchers undertaking this study.**	
**5. I am aware of who I should contact if I wish to lodge a complaint.**	
**6. I voluntarily agree to take part in this study.**	
**7. I understand that information collected in this survey is stored on servers based in the USA.**	

For peer review only

For peer review only - http://bmjopen.bmj.com/site/about/guidelines.xhtml

Screening questions

First, we want to make sure you're a good fit for our study. Please answer the questions below so we can check.

1. Which of the following age groups are you in?

- 18-59 (exclude)
- 60-64
- 65-69
- 70-74
- 75+ (exclude)

2. Which of the following apply to you?

- I have been diagnosed with bowel cancer (exclude)
- I have parts of my bowel been removed (exclude)
- I have had a colonoscopy before (exclude)
- None of the above

3. How many times have you been invited to take a faecal occult blood test (FOBt)? This is a screening test for bowel cancer which is sent in the post to men and women aged between 60 and 74.

- Never (Q6 + exclude)
- 1
- 2
- 3 +

Image of FOBt test kit:

Redacted due to copyright restrictions

---NEXT PAGE---

4. How many times have you completed this test?

- 0 (Q7 + exclude)
- 1
- 2
- 3 +

5. Have you ever had an abnormal test result i.e. you needed further tests?

- No
- Yes (Q8 +exclude)
- Don't remember

For peer review only - http://bmjopen.bmj.com/site/about/guidelines.xhtml

6. ***All adults aged between 60 and 75 should be invited for FOBt screening? Do you know why you have not been invited? (tick all that apply)***
    - I'm a new resident in the UK
    - I am not registered with a GP
    - I have recently moved home
    - I do not have a fixed address

    - Other (please specify)
7. ***What was the reason for not completing the test? (tick all that apply)***
    - I though completing a stool test is distasteful
    - I felt uncomfortable keeping samples of my stool on a test kit in the house
    - I felt uncomfortable sending a stool sample by post
    - I felt uncomfortable about someone looking at my stool sample
    - It is difficult to get round to doing the stool test
    - It was difficult to overcome the embarrassment related to the stool test
    - It was difficult to overcome the disgust related to the stool test

    - Other (please specify)

8. ***Can you remember what follow-up test you had?***
    - Colonoscopy
    - Felixible sigmoidoscopy
    - CT colonography

    - Other (please specify)

---RANDOMIZATION---

For peer review only

Test 1- Colonoscopy

Imagine that instead of a normal result, your test was abnormal. This means that traces of blood were found in your stool. In addition to your test result, you receive an invitation for a follow-up test called colonoscopy. This test looks for polyps and cancer in the large bowel and is performed by a healthcare professional at a hospital. The following table describes this test in detail. Please read this carefully.

Colonoscopy

Preparation	A healthcare professional will determine your eligibility to have a colonoscopy based on your health. Before a colonoscopy, you have to restrict your diet and take a strong laxative.
Test procedure	Before having a colonoscopy, you will be offered a sedative. This is usually given as an injection into a vein in your arm. A long flexible tube with a tiny camera on the end is inserted in your anus in order to examine your bowel. The procedure takes around 30 minutes to complete.<br>Illustration of colonoscopy procedure:<br>Redacted due to copyright restrictions
After the test	If you have the sedative, you will need to arrange for someone to take you home as you may feel drowsy. You will probably feel like resting, so you may want to book the whole day away from your commitments.
What if something is found?	Most polyps (small growths) that are found during the procedure can be removed straight away.
Risks	The procedure can be painful which is why you are offered a sedative. In a small number of cases the test can damage the bowel and cause bleeding (which may require surgery). The test can also miss cancer (about 5 out of 100).
What if I'm diagnosed with cancer	The main treatment for bowel cancer is surgery. In some cases, chemotherapy or radiotherapy may be offered. If the cancer is in a polyp that has been removed during colonoscopy, regular check-ups may be all that is needed. If bowel cancer is detected at the earliest stage, there is over 90% chance of survival.

--NEXT PAGE: COMPREHENSION CHECK—

Please read the following statements and indicate whether they are true or false.

For peer review only - http://bmjopen.bmj.com/site/about/guidelines.xhtml

9. ***Please read the following statements and select the one which best describes colonoscopy.***
   a) **A long flexible tube with a tiny camera tube is inserted in your anus in order to examine your bowel. Polyps are removed during the procedure. The procedure takes about 30 minutes.**
   b) A long flexible tube with a tiny camera tube is inserted in your anus in order to examine your bowel. If polyps are found, you will need a further examination. The procedure takes about 30 minutes.
   c) A long flexible tube with a tiny camera tube is inserted in your anus in order to examine your bowel. Polyps are removed during the procedure. The procedure takes about 60 minutes.

*If answer is incorrect (correct answers in bold), present information again and ask question again until answered correctly.

--NEXT PAGE--

10. ***a) Considering all the information presented above, would you take up the offer of this test?***
   - Definitely not [Send to page 13]
   - Probably not [Send to page 13]
   - Probably yes [Send to page 19]
   - Definitely yes [Send to page 19]

11. ***a) Please read each statement and select how strongly you agree or disagree with it (Strongly agree/Slightly agree/Slightly disagree/Strongly disagree)?***

Emotional barriers
   - The preparation for the test (restricted diet and strong laxative) puts me off
   - The test looks like it would be uncomfortable
   - I would be embarrassed about taking the test
   - I would worry about the risks associated with the test
   - I would be afraid of getting an abnormal result
   - Doing the test would make me worry more about bowel cancer

Practical barriers
   - I would not have time to do the test
   - I have other problems to worry about
   - It would be difficult to arrange transport to the hospital
   - I have other health problems that are more important
   - None of the above

   - Other (free text)

--NEXT PAGE--

For peer review only

Test 2

Imagine that instead of a normal result, your test was abnormal. This means that traces of blood were found in your stool. In addition to your test result, you receive an invitation for a follow-up test called capsule endoscopy. This test looks for polyps and cancer in the large bowel and is performed by a healthcare professional at a hospital. The following table describes this test in detail. Please read this carefully.

--INFORMATION ABOUT TEST—

Capsule endoscopy

Preparation	A healthcare professional will determine your eligibility to have a colon capsule based on your health. Before taking the colon capsule, you have to restrict your diet and take a strong laxative.
Test procedure	You swallow a capsule which contains a small camera. This allows for pictures to be taken of the inside of your bowel which are transmitted wirelessly to a data recorder worn around your waist. The camera is disposable and will pass naturally in your stool.<br>Image of capsule: Redacted due to copyright restrictions<br>Illustration of colon capsule in the bowel: Redacted due to copyright restrictions
After the test	It takes about 8 hours for the capsule to pass through your body. However, after taking the capsule you will be able to carry on with your normal activities.
What if something is found?	If polyps (small growths) are found during the procedure, you will be invited for a further examination, most likely colonoscopy.
Risks	In a small number of cases, the capsule may become stuck and may require surgical removal. This may cause abdominal pain, nausea and vomiting. The test can miss cancer (around 5 in 100).
What if I'm diagnosed with cancer	The main treatment for bowel cancer is surgery. In some cases, chemotherapy or radiotherapy may be offered. If the cancer is in a polyp that has been removed during colonoscopy, regular check-ups may be all that is needed. If bowel cancer is detected at the earliest stage, there is over 90% chance of survival.

--NEXT PAGE--

Please read the following statements and indicate whether they are true or false.

12. Please read the following statements and select the one which best describes capsule endoscopy.

a) The camera used in capsule endoscopy is disposable and will pass naturally in your stool after 8 hours. If polyps are found, they can be removed during the procedure.

b) The camera used in capsule endoscopy is disposable and will pass naturally in your stool after 8 hours. If polyps are found during the procedure, you will be invited for a further examination, most likely colonoscopy.

c) The camera used in capsule endoscopy is disposable and will pass naturally in your stool after 3 hours. If polyps are found during the procedure, you will be invited for a further examination, most likely colonoscopy.

*If answer is incorrect (correct answers in bold), present information again and ask question again until answered correctly.

13. a) Considering all the information presented above, would you take up the offer of this test?

- Definitely not [Send to page 17]
- Probably not [Send to page 17]
- Probably yes [Send to page 19]
- Definitely yes [Send to page 19]

14. a) Please read each statement and select how strongly you agree or disagree with it (Strongly agree/Slightly agree/Slightly disagree/Strongly disagree)?

Emotional barriers

- The preparation for the test (restricted diet and strong laxative) puts me off
- The test looks like it would be uncomfortable
- I would be embarrassed about taking the test
- I would worry about the risks associated with the test
- I would be afraid of getting an abnormal result
- Doing the test would make me worry more about bowel cancer

Practical barriers

- I would not have time to do the test
- I have other problems to worry about
- It would be difficult to arrange transport to the hospital
- I have other health problems that are more important
- None of the above

- Other (free text)

--NEXT PAGE--

For peer review only

Test 3

Imagine that instead of a normal result, your test was abnormal. This means that traces of blood were found in your stool. In addition to your test result, you receive an invitation for a follow-up test called CT colonography. This test looks for polyps and cancer in the large bowel and is performed by a healthcare professional at a hospital. The following table describes this test in detail. Please read this carefully.

--INFORMATION ABOUT TEST—

CT colonography

Preparation	Before having the scan, you have to restrict your diet and take a laxative.
Test procedure	Before having a CT colonography, you may be given an injection of a medicine to relax the bowel. A radiographer then puts a small tube a few centimetres long into your back passage (rectum) to pump carbon dioxide or air inside. This opens the bowel, helping to get clear scans of the inside of your bowel. X-rays are used to make images of your bowel. You may be given an injection of a contrast medium (x-ray dye) to help collect more information from the scan. The procedure takes around 20 minutes to complete.<br><br>Image of CT scanner: Redacted due to copyright restrictions<br><br>Image of CT scans: Redacted due to copyright restrictions
After the test	You may wish to rest at home for the rest of the day, although most people are able to resume their normal daily activities.
What if something is found?	If polyps (small growths) are found during the procedure, you will be invited for a further examination, most likely colonoscopy.
Risks	The procedure may cause some discomfort and you may feel faint. In a very small number of cases the test can damage the bowel which may require surgery. There is also a very small risk that you will have a reaction to the injections. You will be exposed to a small amount of radiation. The test can miss cancer (around 5 in 100).
What if I'm diagnosed with cancer	The main treatment for bowel cancer is surgery. In some cases, chemotherapy or radiotherapy may be offered. If the cancer is in a polyp that has been removed during colonoscopy, regular check-ups may be all that is needed. If bowel cancer is detected at the earliest stage, there is over 90% chance of survival.

--NEXT PAGE--

Please read the following statements and indicate whether they are true or false.

For peer review only - http://bmjopen.bmj.com/site/about/guidelines.xhtml

15. Please read the following statements and select the one which best describes CT colonography.

a) X-rays are used to make images of your bowel. If polyps are found, they can be removed during the procedure. The procedure takes about 20 minutes to complete.
b) X-rays are used to make images of your bowel. If polyps are found during the procedure, you will be invited for a further examination, most likely colonoscopy. The procedure takes about 20 minutes to complete.
c) X-rays are used to make images of your bowel. If polyps are found during the procedure, you will be invited for a further examination, most likely colonoscopy. The procedure takes about 60 minutes to complete.

*If answer is incorrect (correct answers in bold), present information again and ask question again until answered correctly.

16. a) Considering all the information presented above, would you take up the offer of this test?

- Definitely not [Send to page 15]
- Probably not [Send to page 15]
- Probably yes [Send to page 19]
- Definitely yes [Send to page 19]

17. a) Please read each statement and select how strongly you agree or disagree with it (Strongly agree/Slightly agree/Slightly disagree/Strongly disagree)?

Emotional barriers

- The preparation for the test (restricted diet and strong laxative) puts me off
- The test looks like it would be uncomfortable
- I would be embarrassed about taking the test
- I would worry about the risks associated with the test
- I would be afraid of getting an abnormal result
- Doing the test would make me worry more about bowel cancer

Practical barriers

- I would not have time to do the test
- I have other problems to worry about
- It would be difficult to arrange transport to the hospital
- I have other health problems that are more important
- None of the above

- Other (free text)

--NEXT PAGE--

For peer review only

Colonoscopy decliners

You said that you probably or definitely wouldn't have a colonoscopy. The following table describes two other tests that you could be invited to instead. Please read this carefully.

	CT colonography	***Capsule endoscopy***
Preparation	Before having the scan, you have to restrict your diet and take a laxative.	A healthcare professional will determine your eligibility to have a colon capsule based on your health. Before taking the colon capsule, you have to restrict your diet and take a strong laxative.
Test procedure	Before having a CT colonography, you may be given an injection of a medicine to relax the bowel. A radiographer then puts a small tube a few centimetres long into your back passage (rectum) to pump carbon dioxide or air inside. This opens the bowel, helping to get clear scans of the inside of your bowel. X-rays are used to make images of your bowel. You may be given an injection of a contrast medium (x-ray dye) to help collect more information from the scan. The procedure takes around 20 minutes to complete.<br><br>Image of CT scanner: Redacted due to copyright restrictions<br><br>Image of CT scans: Redacted due to copyright restrictions	You swallow a capsule which contains a small camera. This allows for pictures to be taken of the inside of your bowel which are transmitted wirelessly to a data recorder worn around your waist. The camera is disposable and will pass naturally in your stool.<br><br>Image of capsule: Redacted due to copyright restrictions<br><br>Illustration of colon capsule in the bowel: Redacted due to copyright restrictions
After the test	You may wish to rest at home for the rest of the day, although most people are able to resume their normal daily activities.	It takes about 8 hours for the capsule to pass through your body. However, after taking the capsule you will be able to carry on with your normal activities.
What if something is found?	If polyps (small growths) are found during the procedure, you will be invited for a further examination, most likely colonoscopy.	If polyps (small growths) are found during the procedure, you will be invited for a further examination, most likely colonoscopy.
Risks	The procedure may cause some discomfort and you may feel faint. In a very small number of cases the test can damage the bowel which may require surgery. There is also a very small risk that you will have a reaction to the injections. You will be exposed to a small amount of radiation. The test can miss cancer (around 5 in 100).	In a small number of cases, the capsule may become stuck and may require surgical removal. This may cause abdominal pain, nausea and vomiting. The test can miss cancer (around 5 in 100).
What if I'm diagnosed with cancer	The main treatment for bowel cancer is surgery. In some cases, chemotherapy or radiotherapy may be offered. If the cancer is in a polyp that has been removed during colonoscopy, regular check-ups may be all that is needed. If bowel cancer is detected at the earliest stage, there is over 90% chance of survival.	

For peer review only - http://bmjopen.bmj.com/site/about/guidelines.xhtml

18. Considering all the information presented above, would you take up the offer of CT colonography?

- Definitely not
- Probably not
- Probably yes
- Probably yes

19. Considering all the information presented above, would you take up the offer of capsule endoscopy?

- Definitely not
- Probably not
- Probably yes
- Probably yes

20. Which of the two tests would you prefer to have? (choose one)

- CT colonography
- Capsule endoscopy

For peer review only

CT colonography decliners

You said that you probably or definitely wouldn't have a CT colonography. The following table describes two other tests that you could be invited to instead. Please read this carefully.

	**Colonoscopy**	**Capsule endoscopy**
Preparation	A healthcare professional will determine your eligibility to have a colonoscopy based on your health. Before a colonoscopy, you have to restrict your diet and take a strong laxative.	A healthcare professional will determine your eligibility to have a colon capsule based on your health. Before taking the colon capsule, you have to restrict your diet and take a strong laxative.
Test procedure	Before having a colonoscopy, you will be offered a sedative. This is usually given as an injection into a vein in your arm. A long flexible tube with a tiny camera on the end is inserted in your anus in order to examine your bowel. The procedure takes around 30 minutes to complete.	You swallow a capsule which contains a small camera. This allows for pictures to be taken of the inside of your bowel which are transmitted wirelessly to a data recorder worn around your waist. The camera is disposable and will pass naturally in your stool.
	Illustration of colonoscopy procedure: Redacted due to copyright restrictions	Image of capsule: Redacted due to copyright restrictions / Illustration of colon capsule in the bowel: Redacted due to copyright restrictions
After the test	If you have the sedative, you will need to arrange for someone to take you home as you may feel drowsy. You will probably feel like resting, so you may want to book the whole day away from your commitments.	It takes about 8 hours for the capsule to pass through your body. However, after taking the capsule you will be able to carry on with your normal activities.
What if something is found?	Most polyps (small growths) that are found during the procedure can be removed straight away.	If polyps (small growths) are found during the procedure, you will be invited for a further examination, most likely colonoscopy.
Risks	The procedure can be painful which is why you are offered a sedative. In a small number of cases the test can damage the bowel and cause bleeding (which may require surgery). The test can also miss cancer (about 5 out of 100).	In a small number of cases, the capsule may become stuck and may require surgical removal. This may cause abdominal pain, nausea and vomiting. The test can miss cancer (around 5 in 100).
What if I'm diagnosed with cancer	The main treatment for bowel cancer is surgery. In some cases, chemotherapy or radiotherapy may be offered. If the cancer is in a polyp that has been removed during colonoscopy, regular check-ups may be all that is needed. If bowel cancer is detected at the earliest stage, there is over 90% chance of survival.	

21. Considering all the information presented above, would you take up the offer of colonoscopy?

- Definitely not

For peer review only - http://bmjopen.bmj.com/site/about/guidelines.xhtml

- Probably not
- Probably yes
- Probably yes

22. Considering all the information presented above, would you take up the offer of capsule endoscopy?

- Definitely not
- Probably not
- Probably yes
- Probably yes

23. Which of the two tests would you prefer to have? (choose one)

- Colonoscopy
- Capsule endoscopy

For peer review only

Capsule endoscopy decliners

You said that you probably or definitely wouldn't have capsule endoscopy. The following table describes two other tests that you could be invited to instead. Please read this carefully.

	Colonoscopy	***CT colonography***
Preparation	A healthcare professional will determine your eligibility to have a colonoscopy based on your health. Before a colonoscopy, you have to restrict your diet and take a strong laxative.	Before having the scan, you have to restrict your diet and take a laxative.
Test procedure	Before having a colonoscopy, you will be offered a sedative. This is usually given as an injection into a vein in your arm. A long flexible tube with a tiny camera on the end is inserted in your anus in order to examine your bowel. The procedure takes around 30 minutes to complete.<br>Illustration of colonoscopy procedure:<br>Redacted due to copyright restrictions	Before having a CT colonography, you may be given an injection of a medicine to relax the bowel. A radiographer then puts a small tube a few centimetres long into your back passage (rectum) to pump carbon dioxide or air inside. This opens the bowel, helping to get clear scans of the inside of your bowel. X-rays are used to make images of your bowel. You may be given an injection of a contrast medium (x-ray dye) to help collect more information from the scan. The procedure takes around 20 minutes to complete.<br>Image of CT scanner:<br>Redacted due to copyright restrictions<br>Image of CT scans:<br>Redacted due to copyright restrictions
After the test	If you have the sedative, you will need to arrange for someone to take you home as you may feel drowsy. You will probably feel like resting, so you may want to book the whole day away from your commitments.	You may wish to rest at home for the rest of the day, although most people are able to resume their normal daily activities.
What if something is found?	Most polyps (small growths) that are found during the procedure can be removed straight away.	If polyps (small growths) are found during the procedure, you will be invited for a further examination, most likely colonoscopy.
Risks	The procedure can be painful which is why you are offered a sedative. In a small number of cases the test can damage the bowel and cause bleeding (which may require surgery). The test can also miss cancer (about 5 out of 100).	The procedure may cause some discomfort and you may feel faint. In a very small number of cases the test can damage the bowel which may require surgery. There is also a very small risk that you will have a reaction to the injections. You will be exposed to a small amount of radiation. The test can miss cancer (around 5 in 100).
What if I'm diagnosed with cancer	The main treatment for bowel cancer is surgery. In some cases, chemotherapy or radiotherapy may be offered. If the cancer is in a polyp that has been removed during colonoscopy, regular check-ups may be all that is needed. If bowel cancer is detected at the earliest stage, there is over 90% chance of survival.	

For peer review only - http://bmjopen.bmj.com/site/about/guidelines.xhtml

25. Considering all the information presented above, would you take up the offer of colonoscopy?

- Definitely not
- Probably not
- Probably yes
- Probably yes

26. Considering all the information presented above, would you take up the offer of CT colonography?

- Definitely not
- Probably not
- Probably yes
- Probably yes

27. Which of the two tests would you prefer to have? (choose one)

- Colonoscopy
- CT colonography

For peer review only

Socio- demographic questions

28. Are you…?

- Male
- Female

29. Are you currently…?

- Employed full-time
- Employed part-time
- Self-employed
- Unemployed
- Full-time homemaker
- Retired
- Student
- Disabled or too ill to work

30. Does your household have a car of van?

- No
- Yes

31. Which best describes your living arrangement?

- Rent privately or from council, local authority or housing association
- Own your home / have a mortgage

32. Which of the following describes your ethnic group?

- White British
- Other White background
- Black background
- Asian background
- Mixed background
- Any other

33. What is your highest educational qualification?

- No qualifications
- GCSE/O Level, vocational level 2 and equivalents
- A levels, vocational level 3 and equivalents
- Higher Education & professional/vocational equivalents

34. Which of the following numbers represents the biggest risk of getting a disease?

- 1/10
- 1/100
- 1/1000
- I don't know

For peer review only

Table 1. Intention to have the test for colonoscopy, CT colonography, and capsule endoscopy n (%).

N=953	Colonoscopy	Capsule endoscopy	CT colonography
Definitely not	4 (1.3)	6 (1.7)	2 (0.7)
Probably not	21 (6.9)	17 (4.9)	8 (2.7)
Yes, probably	114 (37.4)	123 (35.6)	115 (38.1)
Yes, definitely	166 (54.4)	200 (57.8)	177 (58.6)

Table 2.Emotional barriers to each test n(%)

N=953	Colonoscopy	Capsule endoscopy	CT colonography
**Off-putting**			
Strongly disagree	106 (34.8)	134 (38.7)	122 (40.4)
Slightly disagree	94 (30.8)	116 (33.5)	103 (34.1)
Slightly agree	83 (27.2)	84 (24.3)	67 (22.2)
Strongly agree	22 (7.2)	12 (3.5)	10 (3.3)
**Uncomfortable**			
Strongly disagree	14 (4.6)	87 (25.1)	27 (8.9)
Slightly disagree	32 (10.5)	127 (36.7)	51 (16.9)
Slightly agree	183 (60.0)	119 (34.4)	188 (62.3)
Strongly agree	76 (24.9)	13 (3.8)	36 (11.9)
**Embarrassing**			
Strongly disagree	115 (37.7)	201 (58.1)	135 (44.7)
Slightly disagree	75 (24.6)	89 (25.7)	77 (25.5)
Slightly agree	92 (30.2)	51 (14.7)	76 (25.2)
Strongly agree	23 (7.5)	5 (1.5)	14 (4.6)
**Worry about risks**			
Strongly disagree	82 (26.9)	110 (31.8)	103 (34.1)
Slightly disagree	95 (31.2)	121 (35.0)	95 (31.5)
Slightly agree	111 (36.4)	107 (30.9)	92 (30.5)
Strongly agree	17 (5.6)	8 (2.3)	12 (4.0)
**Afraid of results**			
Strongly disagree	64 (21.0)	87 (25.1)	68 (22.5)
Slightly disagree	54 (17.7)	73 (21.1)	55 (18.2)
Slightly agree	140 (45.9)	154 (44.5)	146 (48.3)
Strongly agree	47 (15.4)	32 (9.3)	33 (10.9)
**Worry about cancer**			
Strongly disagree	87 (28.5)	126 (36.4)	86 (28.5)
Slightly disagree	97 (31.8)	99 (28.6)	85 (28.2)
Slightly agree	94 (30.8)	108 (31.2)	112 (37.1)
Strongly agree	27 (8.9)	13 (3.8)	19 (6.3)

For peer review only

Table 3.Practical barriers to each test n(%)

N=953	Colonoscopy	Capsule endoscopy	CT colonography
**No time**			
Strongly disagree	226 (74.1)	285 (82.4)	246 (81.5)
Slightly disagree	61 (20.0)	55 (15.9)	48 (15.9)
Slightly agree	14 (4.6)	6 (1.7)	6 (2.0)
Strongly agree	4 (1.3)	0 (0.0)	2 (0.7)
**Other problems**			
Strongly disagree	140 (45.9)	182 (52.6)	144 (47.7)
Slightly disagree	88 (28.9)	94 (27.2)	94 (31.1)
Slightly agree	61 (20.0)	61 (17.6)	49 (16.2)
Strongly agree	16 (5.3)	9 (2.6)	15 (5.0)
**Difficulties with transport**			
Strongly disagree	188 (61.6)	239 (69.1)	210 (69.5)
Slightly disagree	67 (22.0)	63 (18.2)	58 (19.2)
Slightly agree	39 (12.8)	39 (11.3)	27 (8.9)
Strongly agree	11 (3.6)	5 (1.5)	7 (2.3)
**Health problems**			
Strongly disagree	183 (60.0)	218 (63.0)	209 (68.9)
Slightly disagree	85 (27.9)	96 (27.8)	71 (23.5)
Slightly agree	27 (8.9)	26 (7.5)	14 (4.6)
Strongly agree	10 (3.3)	6 (1.7)	9 (3.0)

For peer review only

```
Thursday September 5 11:12:06 2019   Page 1

                                                     ___  ____  ____  ____  ____(R)
                                                    /__    /   ____/   /   ____/
                                                   ___/   /   /___/   /   /___/
                                                     Statistics/Data Analysis

      name:  <unnamed>
       log:  S:\FPHS_BSH_CancerComm\QQ PROJECTS 2012-17\BOWEL SCREENING PROJECT\Alternatives to
  log type:  smcl
 opened on:   5 Sep 2019, 11:08:18

1 . use "S:\FPHS_BSH_CancerComm\QQ PROJECTS 2012-17\BOWEL SCREENING PROJECT\Alternatives to Colon

2 .
3 . //Intention and BAME interaction//
4 . logit int2 i.cond##i.ethnicity i.cage i.female i.idep i.paid i.num if comp==1, or

note: 1.cond#1.ethnicity != 0 predicts success perfectly
      1.cond#1.ethnicity dropped and 4 obs not used

note: 3.cond#1.ethnicity omitted because of collinearity
Iteration 0:   log likelihood = -218.29837
Iteration 1:   log likelihood = -215.64434
Iteration 2:   log likelihood = -206.51414
Iteration 3:   log likelihood = -199.21258
Iteration 4:   log likelihood = -197.68211
Iteration 5:   log likelihood = -197.37916
Iteration 6:   log likelihood = -197.37691
Iteration 7:   log likelihood = -197.37691

Logistic regression                             Number of obs     =        949
                                                LR chi2(11)       =      41.84
                                                Prob > chi2       =     0.0000
Log likelihood = -197.37691                     Pseudo R2         =     0.0958
```

int2	Odds Ratio	Std. Err.	z	P>|z|	[95% Conf.	Interval]
cond						
Capsule	1.440134	.4558544	1.15	0.249	.7744031	2.678175
CT colonography	3.164895	1.327974	2.75	0.006	1.390591	7.203097
ethnicity						
BAME	.0257632	.0289846	-3.25	0.001	.0028403	.2336873
cond#ethnicity						
Colonoscopy#BAME	1	(empty)				
Capsule#BAME	1.525905	2.263561	0.28	0.776	.0833368	27.93948
CT colonography#BAME	1	(omitted)				
cage						
65-69	.7060353	.2438926	-1.01	0.314	.3587465	1.389521
70-74	1.402867	.5943763	0.80	0.424	.6114676	3.218544
female						
Yes	1.06484	.3075379	0.22	0.828	.6045717	1.875517
idep						
1 marker	.4684608	.148785	-2.39	0.017	.2513786	.8730079
2-3 markers	.3456216	.1480694	-2.48	0.013	.1492566	.8003283
paid						
Yes	.7472369	.2478844	-0.88	0.380	.3900185	1.431632
num						
Correct	1.487324	.4351085	1.36	0.175	.8382839	2.638882
_cons	14.26229	6.135926	6.18	0.000	6.137408	33.14312

Note: **_cons** estimates baseline odds.

```
  Thursday September 5 11:12:08 2019   Page 2

5 .
6 . //Intention and deprivation interaction//
7 . logit int2 i.cond##i.idep  i.cage i.female i.ethnicity i.paid i.num if comp==1, or

Iteration 0:   log likelihood = -218.55008
Iteration 1:   log likelihood = -203.06951
Iteration 2:   log likelihood = -199.19849
Iteration 3:   log likelihood = -199.10343
Iteration 4:   log likelihood = -199.10307
Iteration 5:   log likelihood = -199.10307

Logistic regression                             Number of obs     =        953
                                                LR chi2(14)       =      38.89
                                                Prob > chi2       =     0.0004
Log likelihood = -199.10307                     Pseudo R2         =     0.0890
```

int2	Odds Ratio	Std. Err.	z	P>|z|	[95% Conf.	Interval]
cond						
Capsule	1.740958	.7370709	1.31	0.190	.7593019	3.99174
CT colonography	3.305797	1.748944	2.26	0.024	1.172046	9.324114
idep						
1 marker	.5428541	.2653282	-1.25	0.211	.208279	1.414884
2-3 markers	.8346543	.6618163	-0.23	0.820	.1764289	3.948604
cond#idep						
Capsule#1 marker	.7549797	.5310209	-0.40	0.689	.1902111	2.99664
Capsule#2-3 markers	.1872543	.1874193	-1.67	0.094	.0263318	1.33163
CT colonography#1 marker	.6383686	.5497961	-0.52	0.602	.1180246	3.452792
CT colonography#2-3 markers	.4718537	.6540561	-0.54	0.588	.0311833	7.139919
cage						
65-69	.6643002	.2293366	-1.18	0.236	.3376788	1.306848
70-74	1.385225	.5810532	0.78	0.437	.6087953	3.151879
female						
Yes	1.135806	.3270864	0.44	0.658	.6459178	1.997243
ethnicity						
BAME	.0868887	.0536264	-3.96	0.000	.0259188	.2912809
paid						
Yes	.8567131	.2863368	-0.46	0.644	.4449803	1.649415
num						
Correct	1.533339	.44838	1.46	0.144	.8644292	2.719864
_cons	12.76679	5.550371	5.86	0.000	5.44529	29.93245

```
Note: _cons estimates baseline odds.

 8 .
 9 . //Off-putting and gender interaction//
10 . logit datt1 i.cond##i.female i.cage i.ethnicity i.idep i.paid i.num if comp==1, or

Iteration 0:   log likelihood = -575.64795
Iteration 1:   log likelihood = -567.00146
Iteration 2:   log likelihood = -566.95168
Iteration 3:   log likelihood = -566.95168

Logistic regression                             Number of obs     =        954
                                                LR chi2(12)       =      17.39
                                                Prob > chi2       =     0.1354
Log likelihood = -566.95168                     Pseudo R2         =     0.0151
```

For peer review only

```
  Thursday September 5 11:12:08 2019   Page 3

                   datt1 | Odds Ratio   Std. Err.      z    P>|z|     [95% Conf. Interval]
-------------------------+----------------------------------------------------------------
                    cond |
                Capsule  |   .5994014   .1453158    -2.11   0.035     .3726978    .9640035
        CT colonography  |    .602084   .1528489    -2.00   0.046     .3660713    .9902584
                         |
                  female |
                    Yes  |   1.147654   .2795688     0.57   0.572     .7119644    1.849967
                         |
             cond#female |
            Capsule#Yes  |   1.561034   .5367613     1.30   0.195     .7956584    3.062653
    CT colonography#Yes  |   1.193689   .4305356     0.49   0.624     .5886849     2.42047
                         |
                    cage |
                  65-69  |   .9965692   .1806268    -0.02   0.985     .6985996     1.42163
                  70-74  |   1.047513   .2039252     0.24   0.812     .7152387    1.534149
                         |
               ethnicity |
                   BAME  |   2.271933   1.290318     1.44   0.148     .7463898     6.91553
                         |
                    idep |
               1 marker  |   1.061183   .1891052     0.33   0.739     .7483492    1.504792
           2-3 markers   |   1.403437   .3834878     1.24   0.215     .8214909    2.397634
                         |
                    paid |
                    Yes  |   .9865023   .1755985    -0.08   0.939     .6959585     1.39834
                         |
                     num |
                Correct  |   1.039599   .1512154     0.27   0.789     .7817247     1.38254
                   _cons |   .4533808   .1097497    -3.27   0.001     .2821064    .7286403
------------------------------------------------------------------------------------------
Note: _cons estimates baseline odds.

 11 .
 12 . //Embarrasing and gender interaction//
 13 . logit datt3 i.cond##i.female i.cage i.ethnicity i.idep i.paid i.num if comp==1, or

Iteration 0:   log likelihood = -559.79953
Iteration 1:   log likelihood = -526.78936
Iteration 2:   log likelihood = -526.08874
Iteration 3:   log likelihood = -526.08791
Iteration 4:   log likelihood = -526.08791

Logistic regression                             Number of obs     =        954
                                                LR chi2(12)       =      67.42
                                                Prob > chi2       =     0.0000
Log likelihood = -526.08791                     Pseudo R2         =     0.0602

                   datt3 | Odds Ratio   Std. Err.      z    P>|z|     [95% Conf. Interval]
-------------------------+----------------------------------------------------------------
                    cond |
                Capsule  |    .393843   .1049377    -3.50   0.000     .2336277    .6639295
        CT colonography  |   .6117832    .158551    -1.90   0.058     .3681276    1.016709
                         |
                  female |
                    Yes  |   1.802571   .4375261     2.43   0.015     1.120175    2.900674
                         |
             cond#female |
            Capsule#Yes  |   .6950697   .2652284    -0.95   0.340       .32902    1.468366
    CT colonography#Yes  |   1.382337   .4910231     0.91   0.362     .6890595    2.773136
                         |
                    cage |
                  65-69  |   .8078209   .1503881    -1.15   0.252     .5608556    1.163534
                  70-74  |    .889448   .1795823    -0.58   0.562     .5987705    1.321237
                         |
               ethnicity |
                   BAME  |   1.155396   .7401264     0.23   0.822     .3292044    4.055045
                         |
                    idep |
               1 marker  |   .9886713   .1860287    -0.06   0.952     .6837378    1.429599
```

For peer review only

```
  Thursday September 5 11:12:08 2019   Page 4

           2-3 markers |   1.281844   .3718801     0.86   0.392     .7259228    2.263498
                       |
                  paid |
                   Yes |   1.439032   .2589492     2.02   0.043     1.011346    2.047583
                       |
                   num |
               Correct |   1.112985   .1693064     0.70   0.482     .8260481    1.499593
                 _cons |    .420774   .1040708    -3.50   0.000     .2591312    .6832476
------------------------------------------------------------------------------------------
Note: _cons estimates baseline odds.

 14 .
 15 . //Worry about test risk and gender interaction//
 16 . logit datt4 i.cond##i.female i.cage i.ethnicity i.idep i.paid i.num if comp==1, or

Iteration 0:   log likelihood = -625.38047
Iteration 1:   log likelihood = -610.79631
Iteration 2:   log likelihood = -610.75012
Iteration 3:   log likelihood = -610.75012

Logistic regression                             Number of obs     =        954
                                                LR chi2(12)       =      29.26
                                                Prob > chi2       =     0.0036
Log likelihood = -610.75012                     Pseudo R2         =     0.0234

------------------------------------------------------------------------------------------
                 datt4 | Odds Ratio   Std. Err.      z    P>|z|     [95% Conf. Interval]
-----------------------+------------------------------------------------------------------
                  cond |
               Capsule |   .8625152   .1965071    -0.65   0.516     .5518706     1.34802
       CT colonography |   .6402238   .1565886    -1.82   0.068      .396407    1.034004
                       |
                female |
                   Yes |   1.574237    .372587     1.92   0.055     .9899417    2.503402
                       |
           cond#female |
           Capsule#Yes |    .626443   .2078761    -1.41   0.159     .3269057     1.20044
   CT colonography#Yes |   1.312932   .4499751     0.79   0.427     .6706774    2.570221
                       |
                  cage |
                 65-69 |   .8532505   .1461122    -0.93   0.354     .6099795    1.193542
                 70-74 |   .8931162   .1645699    -0.61   0.540     .6223884    1.281606
                       |
             ethnicity |
                  BAME |    1.94679    1.10931     1.17   0.242     .6372233    5.947667
                       |
                  idep |
              1 marker |   1.494679   .2483308     2.42   0.016     1.079261    2.069995
           2-3 markers |   1.271042   .3415635     0.89   0.372     .7506177     2.15229
                       |
                  paid |
                   Yes |   1.121261   .1875352     0.68   0.494     .8078669    1.556228
                       |
                   num |
               Correct |   1.065082   .1471823     0.46   0.648     .8123747    1.396398
                 _cons |    .532697   .1239152    -2.71   0.007     .3376564    .8403991
------------------------------------------------------------------------------------------
Note: _cons estimates baseline odds.

 17 .
```

```
  Thursday September 5 11:12:09 2019   Page 5

18 . //Worry about test risk and deprivation interaction//
19 . logit datt4 i.cond##i.idep i.cage i.female i.ethnicity i.paid i.num if comp==1, or

   Iteration 0:   log likelihood = -625.38047
   Iteration 1:   log likelihood = -609.00743
   Iteration 2:   log likelihood = -608.97148
   Iteration 3:   log likelihood = -608.97148

   Logistic regression                             Number of obs     =        954
                                                   LR chi2(14)       =      32.82
                                                   Prob > chi2       =     0.0031
   Log likelihood = -608.97148                     Pseudo R2         =     0.0262
```

datt4	Odds Ratio	Std. Err.	z	P>|z|	[95% Conf.	Interval]
cond						
Capsule	.8545529	.1688906	-0.80	0.426	.5801105	1.25883
CT colonography	.8738928	.1757055	-0.67	0.503	.5892709	1.295989
idep						
1 marker	2.773085	.8485567	3.33	0.001	1.522291	5.051596
2-3 markers	1.35632	.5985531	0.69	0.490	.5711117	3.221092
cond#idep						
Capsule#1 marker	.3593903	.1484572	-2.48	0.013	.159938	.8075719
Capsule#2-3 markers	1.287068	.7999347	0.41	0.685	.3806846	4.351488
CT colonography#1 marker	.4862063	.2070739	-1.69	0.090	.2110067	1.120327
CT colonography#2-3 markers	.5373902	.3794284	-0.88	0.379	.1346767	2.144307
cage						
65-69	.8475212	.1459977	-0.96	0.337	.6046707	1.187906
70-74	.8790054	.1626208	-0.70	0.486	.6116663	1.263189
female						
Yes	1.454996	.2032422	2.68	0.007	1.106525	1.91321
ethnicity						
BAME	2.084474	1.191957	1.28	0.199	.6796058	6.39346
paid						
Yes	1.107485	.1857581	0.61	0.543	.7971979	1.538543
num						
Correct	1.074747	.1491767	0.52	0.604	.8187623	1.410764
_cons	.4918955	.1081869	-3.23	0.001	.3196396	.7569813

```
   Note: _cons estimates baseline odds.

20 .
21 . //Afraid of results risk and age group interaction//
22 . logit datt5 i.cond##i.cage i.female i.ethnicity i.idep i.paid i.num if comp==1, or

   Iteration 0:   log likelihood = -649.10164
   Iteration 1:   log likelihood =   -626.968
   Iteration 2:   log likelihood = -626.80181
   Iteration 3:   log likelihood = -626.80112
   Iteration 4:   log likelihood = -626.80112

   Logistic regression                             Number of obs     =        954
                                                   LR chi2(14)       =      44.60
                                                   Prob > chi2       =     0.0000
   Log likelihood = -626.80112                     Pseudo R2         =     0.0344
```

For peer review only

```
Thursday September 5 11:12:09 2019   Page 6
```

datt5	Odds Ratio	Std. Err.	z	P>|z|	[95% Conf.	Interval]
cond						
Capsule	.9487913	.2971592	-0.17	0.867	.5135425	1.752932
CT colonography	.8040911	.2395326	-0.73	0.464	.4484756	1.441689
cage						
65-69	.6737809	.1962447	-1.36	0.175	.3807139	1.192446
70-74	.7619691	.2441478	-0.85	0.396	.4066287	1.427831
cond#cage						
Capsule#65-69	.8464529	.3448479	-0.41	0.682	.3809082	1.880985
Capsule#70-74	.618933	.2636364	-1.13	0.260	.2685788	1.426315
CT colonography#65-69	1.124773	.4530285	0.29	0.770	.5107682	2.476887
CT colonography#70-74	1.360613	.5960542	0.70	0.482	.5765568	3.210902
female						
Yes	1.579452	.217962	3.31	0.001	1.205152	2.070003
ethnicity						
BAME	3.903997	3.066424	1.73	0.083	.8373884	18.20087
idep						
1 marker	1.369841	.2341368	1.84	0.066	.9798976	1.91496
2-3 markers	.6881451	.1825012	-1.41	0.159	.4091994	1.157244
paid						
Yes	1.1609	.1946665	0.89	0.374	.835719	1.61261
num						
Correct	1.318004	.1794388	2.03	0.043	1.009323	1.721087
_cons	1.307052	.3353875	1.04	0.297	.7904531	2.161274

Note: **_cons** estimates baseline odds.

```
23 .
24 . //Afraid of results risk and gender interaction//
25 . logit datt5 i.cond##i.female i.cage i.ethnicity i.idep i.paid i.num if comp==1, or

Iteration 0:   log likelihood = -649.10164
Iteration 1:   log likelihood = -628.77429
Iteration 2:   log likelihood = -628.62076
Iteration 3:   log likelihood = -628.62007
Iteration 4:   log likelihood = -628.62007

Logistic regression                             Number of obs     =        954
                                                LR chi2(12)       =      40.96
                                                Prob > chi2       =     0.0000
Log likelihood = -628.62007                     Pseudo R2         =     0.0316
```

datt5	Odds Ratio	Std. Err.	z	P>|z|	[95% Conf.	Interval]
cond						
Capsule	.7984076	.1756849	-1.02	0.306	.5187078	1.228928
CT colonography	.9635103	.2206762	-0.16	0.871	.6150389	1.50942
female						
Yes	1.706078	.413164	2.21	0.027	1.061358	2.742432
cond#female						
Capsule#Yes	.8715473	.286892	-0.42	0.676	.4571896	1.661444
CT colonography#Yes	.8956024	.3066038	-0.32	0.747	.457838	1.751938
cage						
65-69	.6722562	.1145439	-2.33	0.020	.4813943	.9387906
70-74	.7060271	.1287507	-1.91	0.056	.4938519	1.00936
ethnicity						
BAME	3.943815	3.079102	1.76	0.079	.8537837	18.21735

For peer review only

Thursday September 5 11:12:10 2019 Page 7

idep						
1 marker	**1.355455**	**.2308021**	**1.79**	**0.074**	**.9708356**	**1.892452**
2-3 markers	**.6852833**	**.1808773**	**-1.43**	**0.152**	**.408507**	**1.149584**
paid						
Yes	**1.188009**	**.1987038**	**1.03**	**0.303**	**.8559524**	**1.648883**
num						
Correct	**1.321628**	**.1794319**	**2.05**	**0.040**	**1.01285**	**1.72454**
_cons	**1.28226**	**.2930399**	**1.09**	**0.277**	**.8193087**	**2.006802**

Note: **_cons** estimates baseline odds.

```
26 .
27 . //Worry about cancer and gender interaction//
28 . logit datt6 i.cond##i.female i.cage i.ethnicity i.idep i.paid i.num if comp==1, or

Iteration 0:   log likelihood = -638.84517
Iteration 1:   log likelihood = -629.17662
Iteration 2:   log likelihood = -629.16658
Iteration 3:   log likelihood = -629.16658

Logistic regression                             Number of obs     =        954
                                                LR chi2(12)       =      19.36
                                                Prob > chi2       =     0.0803
Log likelihood = -629.16658                     Pseudo R2         =     0.0152
```

datt6	Odds Ratio	Std. Err.	z	P>|z|	[95% Conf.	Interval]
cond						
Capsule	**.8870318**	**.1995902**	**-0.53**	**0.594**	**.5707042**	**1.378692**
CT colonography	**1.139612**	**.2635305**	**0.57**	**0.572**	**.7243027**	**1.793056**
female						
Yes	**1.116424**	**.2651117**	**0.46**	**0.643**	**.7009684**	**1.778115**
cond#female						
Capsule#Yes	**.8280574**	**.2740884**	**-0.57**	**0.569**	**.4328239**	**1.584198**
CT colonography#Yes	**1.021262**	**.3416495**	**0.06**	**0.950**	**.5301261**	**1.967412**
cage						
65-69	**.6718341**	**.1127177**	**-2.37**	**0.018**	**.4835605**	**.9334119**
70-74	**.7439943**	**.1335862**	**-1.65**	**0.100**	**.5232798**	**1.057804**
ethnicity						
BAME	**1.742306**	**.9845806**	**0.98**	**0.326**	**.5755841**	**5.274**
idep						
1 marker	**1.33407**	**.2194894**	**1.75**	**0.080**	**.9663482**	**1.84172**
2-3 markers	**.9924304**	**.269328**	**-0.03**	**0.978**	**.583041**	**1.689278**
paid						
Yes	**1.015197**	**.1667701**	**0.09**	**0.927**	**.7357331**	**1.400813**
num						
Correct	**1.215624**	**.1644366**	**1.44**	**0.149**	**.9325193**	**1.584677**
_cons	**.6736522**	**.153843**	**-1.73**	**0.084**	**.4305717**	**1.053964**

Note: **_cons** estimates baseline odds.

For peer review only

```
  Thursday September 5 11:12:10 2019   Page 8

29 .
30 . //No time about BAME interaction//
31 . logit datt7 i.cond##i.ethnicity i.cage i.female i.idep i.paid i.num if comp==1, or

   note: 1.cond#1.ethnicity != 0 predicts failure perfectly
         1.cond#1.ethnicity dropped and 4 obs not used

   note: 3.cond#1.ethnicity omitted because of collinearity
   Iteration 0:   log likelihood = -139.95813
   Iteration 1:   log likelihood = -131.43249
   Iteration 2:   log likelihood =    -126.68
   Iteration 3:   log likelihood = -123.90107
   Iteration 4:   log likelihood =  -123.2927
   Iteration 5:   log likelihood = -123.26786
   Iteration 6:   log likelihood = -123.26785

   Logistic regression                             Number of obs     =        950
                                                   LR chi2(11)       =      33.38
                                                   Prob > chi2       =     0.0005
   Log likelihood = -123.26785                     Pseudo R2         =     0.1193

   ------------------------------------------------------------------------------------
                  datt7 | Odds Ratio   Std. Err.      z    P>|z|     [95% Conf. Interval]
   ---------------------+--------------------------------------------------------------
                   cond |
                Capsule |    .225937   .1167816    -2.88   0.004     .0820391    .6222342
       CT colonography  |   .3317475   .1608767    -2.28   0.023     .1282414     .858197
                        |
              ethnicity |
                   BAME |   71.17544   79.73479     3.81   0.000     7.920736    639.5798
                        |
         cond#ethnicity |
       Colonoscopy#BAME |          1  (empty)
           Capsule#BAME |   .2360667   .3916317    -0.87   0.384     .0091391    6.097719
   CT colonography#BAME |          1  (omitted)
                        |
                   cage |
                  65-69 |   .9844457   .4644365    -0.03   0.973     .3904959    2.481801
                  70-74 |   1.270358   .6398335     0.48   0.635     .4733764    3.409147
                        |
                 female |
                    Yes |   .7283684    .280484    -0.82   0.410     .3424253    1.549303
                        |
                   idep |
               1 marker |   1.327865   .5964158     0.63   0.528     .5505965    3.202392
           2-3 markers  |   2.433038   1.419385     1.52   0.127     .7754885    7.633479
                        |
                   paid |
                    Yes |   3.022953   1.249684     2.68   0.007     1.344455    6.796991
                        |
                    num |
                Correct |   .6374225   .2473719    -1.16   0.246     .2979147    1.363839
                  _cons |   .0482849   .0262249    -5.58   0.000     .0166533    .1399981
   ------------------------------------------------------------------------------------
   Note: _cons estimates baseline odds.

32 .
33 . //No time about employment interaction//
34 . logit datt7 i.cond##i.paid i.ethnicity i.cage i.female i.idep i.num if comp==1, or

   Iteration 0:   log likelihood =  -140.0949
   Iteration 1:   log likelihood = -129.40018
   Iteration 2:   log likelihood = -126.37467
   Iteration 3:   log likelihood =  -126.3263
   Iteration 4:   log likelihood = -126.32608
   Iteration 5:   log likelihood = -126.32608

   Logistic regression                             Number of obs     =        954
                                                   LR chi2(12)       =      27.54
                                                   Prob > chi2       =     0.0065
   Log likelihood = -126.32608                     Pseudo R2         =     0.0983
```

For peer review only

Thursday September 5 11:12:10 2019 Page 9

datt7	Odds Ratio	Std. Err.	z	P>|z|	[95% Conf.	Interval]
cond						
Capsule	.4443503	.2622262	-1.37	0.169	.1397664	1.412695
CT colonography	.5453833	.3208585	-1.03	0.303	.1721571	1.727741
paid						
Yes	3.409665	1.811371	2.31	0.021	1.203689	9.65849
cond#paid						
Capsule#Yes	.2084142	.2547593	-1.28	0.200	.0189865	2.287761
CT colonography#Yes	.6946165	.6397408	-0.40	0.692	.1142325	4.223773
ethnicity						
BAME	8.265565	6.210024	2.81	0.005	1.895632	36.04052
cage						
65-69	1.03754	.4843493	0.08	0.937	.4155685	2.5904
70-74	1.229042	.6137913	0.41	0.680	.4618182	3.270861
female						
Yes	.7356454	.2819938	-0.80	0.423	.3470394	1.559403
idep						
1 marker	1.407705	.6223427	0.77	0.439	.591831	3.34831
2-3 markers	2.431126	1.412986	1.53	0.126	.7781867	7.595062
num						
Correct	.5895268	.2287447	-1.36	0.173	.2755658	1.261194
_cons	.0408934	.023251	-5.62	0.000	.0134178	.1246311

Note: **_cons** estimates baseline odds.

```
35 .
36 . //Difficulty with transport and deprivation interaction//
37 . logit datt9 i.cond##i.idep i.cage i.female i.ethnicity i.paid i.num if comp==1, or

Iteration 0:   log likelihood = -376.10599
Iteration 1:   log likelihood = -361.04686
Iteration 2:   log likelihood = -358.60263
Iteration 3:   log likelihood = -358.59978
Iteration 4:   log likelihood = -358.59978

Logistic regression                             Number of obs     =        954
                                                LR chi2(14)       =      35.01
                                                Prob > chi2       =     0.0015
Log likelihood = -358.59978                     Pseudo R2         =     0.0465
```

datt9	Odds Ratio	Std. Err.	z	P>|z|	[95% Conf.	Interval]
cond						
Capsule	.6689234	.1965032	-1.37	0.171	.3761206	1.189668
CT colonography	.5796323	.1791188	-1.76	0.078	.3163101	1.062165
idep						
1 marker	1.551238	.5872859	1.16	0.246	.738619	3.257889
2-3 markers	2.259239	1.1653	1.58	0.114	.8220878	6.208779
cond#idep						
Capsule#1 marker	1.046429	.5651208	0.08	0.933	.3630955	3.015773
Capsule#2-3 markers	2.351092	1.660775	1.21	0.226	.5888354	9.387398
CT colonography#1 marker	1.206273	.6866612	0.33	0.742	.3952813	3.681165
CT colonography#2-3 markers	1.584024	1.237581	0.59	0.556	.3425522	7.324815
cage						
65-69	.7956792	.189795	-0.96	0.338	.498539	1.269922
70-74	.6638411	.1776491	-1.53	0.126	.3928945	1.121637
female						
Yes	1.310302	.259888	1.36	0.173	.8882644	1.93286

Thursday September 5 11:12:11 2019 Page 10

ethnicity						
BAME	3.29872	2.040796	1.93	0.054	.9811572	11.09053
paid						
Yes	.8836746	.2128248	-0.51	0.608	.5511736	1.41676
num						
Correct	.7531362	.1499798	-1.42	0.155	.5097591	1.11271
_cons	.2011304	.0606306	-5.32	0.000	.1113996	.363138

Note: **_cons** estimates baseline odds.

```
38 .
39 . //Health problems and gender interaction//
40 . logit datt10 i.cond##i.female i.cage i.ethnicity i.idep i.paid i.num if comp==1, or

Iteration 0:   log likelihood = -302.59055
Iteration 1:   log likelihood = -293.95422
Iteration 2:   log likelihood = -289.70446
Iteration 3:   log likelihood = -289.54772
Iteration 4:   log likelihood = -289.54738
Iteration 5:   log likelihood = -289.54738

Logistic regression                             Number of obs     =        954
                                                LR chi2(12)       =      26.09
                                                Prob > chi2       =     0.0104
Log likelihood = -289.54738                     Pseudo R2         =     0.0431
```

datt10	Odds Ratio	Std. Err.	z	P>|z|	[95% Conf.	Interval]
cond						
Capsule	.8579749	.2803729	-0.47	0.639	.4521839	1.627924
CT colonography	.6854997	.245683	-1.05	0.292	.3395759	1.383814
female						
Yes	.7217563	.2606936	-0.90	0.367	.355584	1.465004
cond#female						
Capsule#Yes	.5703324	.3195649	-1.00	0.316	.1901903	1.710282
CT colonography#Yes	.6423506	.3828119	-0.74	0.458	.1997535	2.065617
cage						
65-69	.9063112	.2517804	-0.35	0.723	.5257834	1.56224
70-74	.8010558	.2442216	-0.73	0.467	.4407101	1.456037
ethnicity						
BAME	6.379531	3.809814	3.10	0.002	1.97905	20.56462
idep						
1 marker	1.348598	.3652431	1.10	0.269	.7931407	2.293057
2-3 markers	2.626326	.9337083	2.72	0.007	1.308369	5.271895
paid						
Yes	.9490002	.2590525	-0.19	0.848	.5557895	1.6204
num						
Correct	.9549643	.2155266	-0.20	0.838	.6135909	1.486262
_cons	.1505126	.0531107	-5.37	0.000	.0753726	.3005608

Note: **_cons** estimates baseline odds.

For peer review only

```
Thursday September 5 11:12:11 2019   Page 11

41 .
42 . //Health problems and BAME interaction//
43 . logit datt10 i.cond##i.ethnicity i.cage i.female i.idep i.paid i.num if comp==1, or

Iteration 0:   log likelihood = -302.59055
Iteration 1:   log likelihood = -294.25034
Iteration 2:   log likelihood = -292.74312
Iteration 3:   log likelihood = -290.23031
Iteration 4:   log likelihood = -289.55165
Iteration 5:   log likelihood = -289.50732
Iteration 6:   log likelihood = -289.50731

Logistic regression                             Number of obs     =        954
                                                LR chi2(12)       =      26.17
                                                Prob > chi2       =     0.0102
Log likelihood = -289.50731                     Pseudo R2         =     0.0432
```

datt10	Odds Ratio	Std. Err.	z	P>|z|	[95% Conf.	Interval]
cond						
Capsule	.6875368	.1819878	-1.42	0.157	.4092482	1.155061
CT colonography	.5434485	.158273	-2.09	0.036	.3070831	.9617471
ethnicity						
BAME	2.668504	3.201338	0.82	0.413	.2541573	28.01774
cond#ethnicity						
Capsule#BAME	2.369605	3.631279	0.56	0.573	.1175603	47.76299
CT colonography#BAME	5.661841	9.042489	1.09	0.278	.2474625	129.5406
cage						
65-69	.9066002	.2523885	-0.35	0.725	.5253512	1.564523
70-74	.7970324	.2438844	-0.74	0.458	.4375385	1.451896
female						
Yes	.5380308	.1284885	-2.60	0.009	.3369222	.859181
idep						
1 marker	1.332997	.3621348	1.06	0.290	.782679	2.270256
2-3 markers	2.5314	.8957882	2.62	0.009	1.26516	5.06496
paid						
Yes	.993958	.2734556	-0.02	0.982	.5796786	1.704311
num						
Correct	.962673	.2180531	-0.17	0.867	.6175538	1.500662
_cons	.1727472	.0568114	-5.34	0.000	.0906727	.3291136

```
Note: _cons estimates baseline odds.

44 .
45 . //Health problems and deprivation interaction//
46 . logit datt10 i.cond##i.dep i.ethnicity i.cage i.female i.paid i.num if comp==1, or

note: 3.cond#3.depr != 0 predicts failure perfectly
      3.cond#3.depr dropped and 1 obs not used

note: 1.cond#3.depr identifies no observations in the sample
note: 2.cond#3.depr omitted because of collinearity
Iteration 0:   log likelihood = -302.48909
Iteration 1:   log likelihood = -294.18407
Iteration 2:   log likelihood = -289.98635
Iteration 3:   log likelihood = -289.83164
Iteration 4:   log likelihood = -289.83128
Iteration 5:   log likelihood = -289.83128

Logistic regression                             Number of obs     =        953
                                                LR chi2(15)       =      25.32
                                                Prob > chi2       =     0.0459
Log likelihood = -289.83128                     Pseudo R2         =     0.0418
```

For peer review only

Thursday September 5 11:12:11 2019 Page 12

datt10	Odds Ratio	Std. Err.	z	P>|z|	[95% Conf.	Interval]
cond						
Capsule	.7088625	.2289895	-1.07	0.287	.3763497	1.335157
CT colonography	.5338427	.1891195	-1.77	0.076	.2666036	1.068958
depr						
1	1.375126	.612255	0.72	0.474	.5745892	3.290999
2	2.160367	1.200865	1.39	0.166	.7267371	6.422109
3	2.412483	2.730994	0.78	0.437	.2623504	22.18435
cond#depr						
Colonoscopy#3	1	(empty)				
Capsule#1	.8794388	.5557166	-0.20	0.839	.2548779	3.034444
Capsule#2	1.193254	1.049022	0.20	0.841	.2130191	6.684163
Capsule#3	1	(omitted)				
CT colonography#1	1.116122	.7644453	0.16	0.873	.2915502	4.272773
CT colonography#2	1.624588	1.464046	0.54	0.590	.2777523	9.502306
CT colonography#3	1	(empty)				
ethnicity						
BAME	6.434172	3.838078	3.12	0.002	1.998658	20.71319
cage						
65-69	.936774	.2627153	-0.23	0.816	.5406523	1.623124
70-74	.8234025	.2533449	-0.63	0.528	.4505199	1.504909
female						
Yes	.53614	.128135	-2.61	0.009	.3356178	.8564686
paid						
Yes	.9559571	.2622917	-0.16	0.870	.5583276	1.63677
num						
Correct	.9552938	.2165329	-0.20	0.840	.61263	1.489621
_cons	.1700755	.0577478	-5.22	0.000	.0874225	.3308722

Note: **_cons** estimates baseline odds.

```
47 .
48 . log close
      name:  <unnamed>
       log:  S:\FPHS_BSH_CancerComm\QQ PROJECTS 2012-17\BOWEL SCREENING PROJECT\Alternatives to
  log type:  smcl
 closed on:   5 Sep 2019, 11:08:22
```

For peer review only
